# Causal relationship between sarcopenia with osteoarthritis and the mediating role of obesity: a univariate, multivariate, two-step Mendelian randomization study

**DOI:** 10.1186/s12877-024-05098-8

**Published:** 2024-05-29

**Authors:** Zicheng Jin, Rui Wang, Linzi Jin, Lishuang Wan, Yuzhou Li

**Affiliations:** 1 College of Physical Education, Henan Normal University, Xinxiang, 453007 China; 2 College of Music and Dance, Henan Normal University, Xinxiang, 453007 China

**Keywords:** Mendelian randomization, Knee osteoarthritis, Hip osteoarthritis, Sarcopenia, Obesity, Circulating inflammatory proteins

## Abstract

**Background:**

Recent genetic evidence supports a causal role for sarcopenia in osteoarthritis, which may be mediated by the occurrence of obesity or changes in circulating inflammatory protein levels. Here, we leveraged publicly available genome-wide association study data to investigate the intrinsic causal relationship between sarcopenia, obesity, circulating inflammatory protein levels, and osteoarthritis.

**Methods:**

In this study, we used Mendelian randomization analyses to explore the causal relationship between sarcopenia phenotypes (Appendicular lean mass [ALM], Low hand-grip strength [LHG], and usual walking pace [UWP]) and osteoarthritis (Knee osteoarthritis [KOA], and Hip osteoarthritis [HOA]). Univariable Mendelian randomization (UVMR) analyses were performed using the inverse variance weighted (IVW) method, MR-Egger, weighted median method, simple mode, and weighted mode, with the IVW method being the primary analytical technique. Subsequently, the independent causal effects of sarcopenia phenotype on osteoarthritis were investigated using multivariate Mendelian randomization (MVMR) analysis. To further explore the mechanisms involved, obesity and circulating inflammatory proteins were introduced as the mediator variables, and a two-step Mendelian randomization analysis was used to explore the mediating effects of obesity and circulating inflammatory proteins between ALM and KOA as well as the mediating proportions.

**Results:**

UVMR analysis showed a causal relationship between ALM, LHG, UWP and KOA [(OR = 1.151, 95% CI: 1.087–1.218, *P* = 1.19 × 10^–6^, P_FDR_ = 7.14 × 10^–6^) (OR = 1.215, 95% CI: 1.004–1.470; *P* = 0.046, P_FDR_ = 0.055) (OR = 0.503, 95% CI: 0.292–0.867; *P* = 0.013, P_FDR_ = 0.027)], and a causal relationship between ALM, UWP and HOA [(OR = 1.181, 95% CI: 1.103–1.265, *P* = 2.05 × 10^–6^, P_FDR_ = 6.15 × 10^–6^) (OR = 0.438, 95% CI: 0.226–0.849, *P* = 0.014, P_FDR_ = 0.022)]. In the MVMR analyses adjusting for confounders (body mass index, insomnia, sedentary behavior, and bone density), causal relationships were observed between ALM, LHG, UWP and KOA [(ALM: OR = 1.323, 95%CI: 1.224- 1.431, *P* = 2.07 × 10^–12^), (LHG: OR = 1.161, 95%CI: 1.044- 1.292, *P* = 0.006), (UWP: OR = 0.511, 95%CI: 0.290- 0.899, *P* = 0.020)], and between ALM and HOA (ALM: OR = 1.245, 95%CI: 1.149- 1.348, *P* = 7.65 × 10^–8^). In a two-step MR analysis, obesity was identified to play a potential mediating role in ALM and KOA (proportion mediated: 5.9%).

**Conclusions:**

The results of this study suggest that decreased appendicular lean mass, grip strength, and walking speed increase the risk of KOA and decreased appendicular lean mass increases the risk of HOA in patients with sarcopenia in a European population. Obesity plays a mediator role in the occurrence of KOA due to appendicular lean body mass reduction.

**Supplementary Information:**

The online version contains supplementary material available at 10.1186/s12877-024-05098-8.

## Introduction

Osteoarthritis is a chronic degenerative joint disease, with knee osteoarthritis (KOA) and hip osteoarthritis (HOA) being prevalent types. Clinically, the main manifestations include limited movements, arthralgia, and joint deformity, etc., which severely impact the patients' quality of life and may even lead to disability as the disease worsens [1, 2]. The pathological mechanisms of these two diseases are mainly related to the imbalance of cartilage matrix repair, but the causes of the two diseases remain unknown, typically involving the interaction of multiple factors, including genetic, mechanical and chemical factors [3, 4]. Mechanically, as important weight-bearing joints, the decline in lower limb muscle strength may lead to decreased joint stability and changes in lower limb force lines, resulting in increased joint stress, increasing the risk of osteoarthritis [5, 6]. Chemically, the reduction of skeletal muscle may cause hormonal regulatory disorders, thereby promoting the occurrence and deterioration of osteoarthritis [7]. Sarcopenia is an age-related chronic degenerative disease characterized by a decrease in overall muscle mass and strength [8]. The decline of the skeletal muscle system in sarcopenia patients may trigger the above mechanical and chemical factors, thereby increasing the risk of KOA and HOA.

Skeletal muscle has metabolic functions, and its reduction can lead to metabolic disorder in the body, affecting energy metabolism and inflammation regulation [9, 10], potentially causing obesity and exacerbating inflammatory responses. Obesity and chronic inflammation are considered as important promotive factors for osteoarthritis [11, 12]. Elevated levels of inflammatory biomarkers, including cytokines and soluble receptors, are closely associated with the onset of osteoarthritis [13]. Additionally, obesity is closely related with the occurrence and deterioration of KOA [14]. Although observational studies show a strong association between sarcopenia and KOA [15], it is unclear whether circulating inflammatory proteins and obesity mediate between sarcopenia and osteoarthritis. Additionally, observational studies have certain limitations in causal inference, making it difficult to establish causality for this association [16]. Therefore, new models are needed to explore the potential risk factors for osteoarthritis, in order to develop more precise and scientific intervention strategies.

Mendelian randomization (MR) is a statistical strategy that employs genetic variation as instrumental variables. Since genetic variation is typically undisturbed by environmental and behavioral factors, this method effectively reduces bias in estimating causal relationships [17]. MR analysis includes both Univariate Mendelian randomization (UVMR) and Multivariable Mendelian randomization (MVMR). MVMR, as an extension of UVMR, can explore the independent causal relationships between exposure factors and outcome factors, and it can also address the issue of multiple exposure factors and the mutual influence of similar exposure factors [18, 19]. This study collected large-scale data from genome-wide association study (GWAS) for UVMR and MVMR analyses, to access the causality of sarcopenia and related phenotypes with KOA and HOA, providing theoretical basis for their prevention and occurrence mechanism studies. Simultaneously, a Two-step Mendelian randomization (Two-step MR) model was used to investigate the mediating effect of obesity and circulating inflammatory proteins between sarcopenia and osteoarthritis, and to calculate the proportion of obesity and circulating inflammatory proteins in this possible mechanism.

## Materials and methods

### Study design

Due to the lack of published GWAS data for sarcopenia, according to the diagnostic criteria for sarcopenia recommended by the European Working Group on Sarcopenia in Older People (EWGSOP), appendicular lean mass (ALM), low hand-grip strength (LHG), and usual walking pace (UWP) were used instead of sarcopenia for MR analysis in this study [20].

In this study, we used UVMR and MVMR analyses to infer causal relationships between myasthenia gravis phenotypes and KOA and HOA. To be a valuable tool for causal inference in MR studies, genetic variation must satisfy three fundamental criteria: Assumption 1, genetic variation as an instrumental variable must be genuinely associated with exposure (sarcopenia phenotypes); assumption 2, exposure-outcome confounders have no effect on genetic variation; and assumption 3, genetic variation affects outcome through exposure (sarcopenia phenotypes) only, independent of other pathways. Previous studies have shown that KOA and HOA can be affected by obesity [21], insomnia [22], sedentary behavior [23], and bone mineral density [24]. To exclude the potential influence of these factors, this study used MVMR analysis to determine the causal associations of sarcopenia phenotypes with KOA and HOA independent of body mass index (BMI), insomnia, TV watching time, and femoral-neck bone mineral density (FN-BMD).

**Fig. 1:**
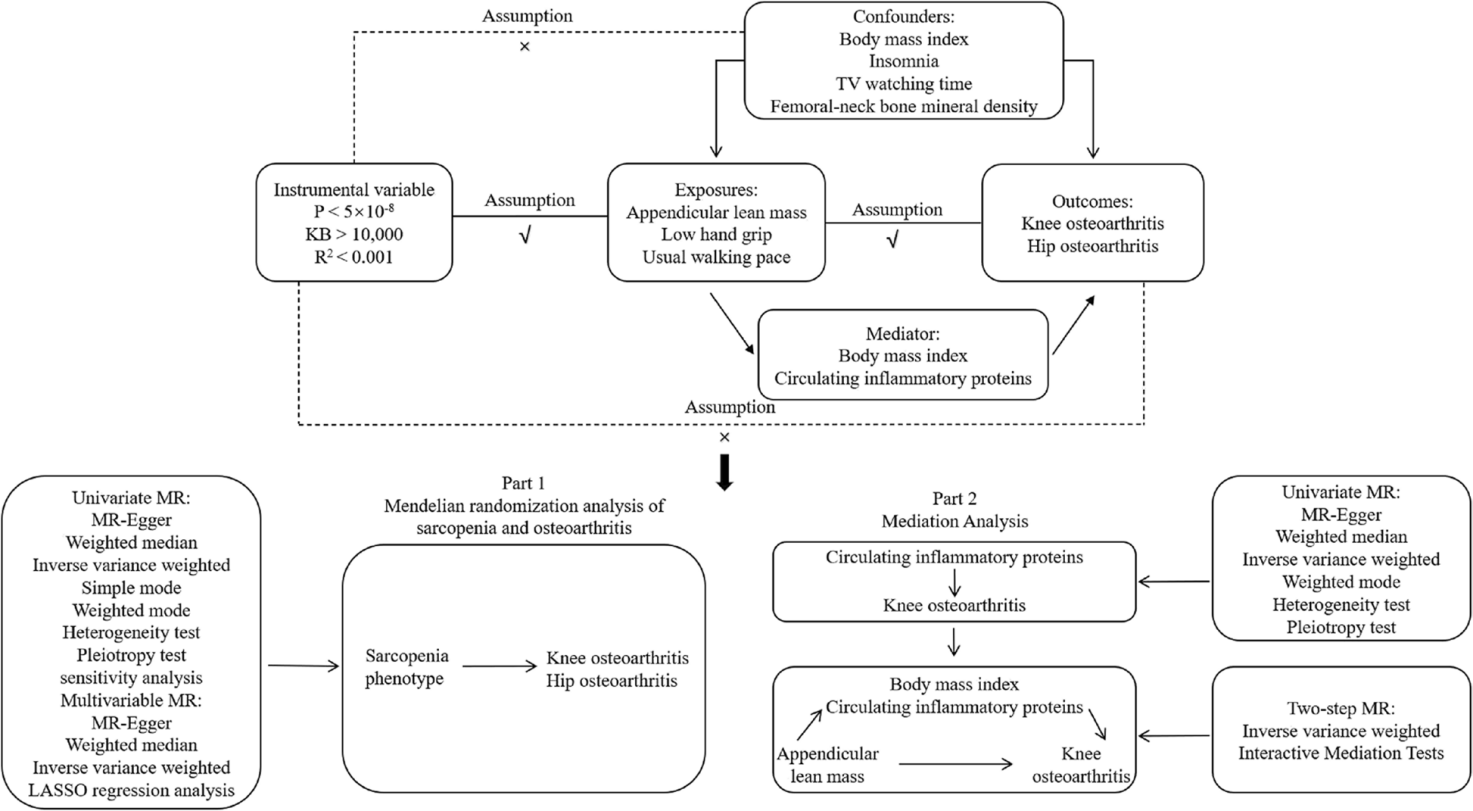
Mendelian randomization analysis flowchart

To further explore the mechanism between sarcopenia and osteoarthritis, the mediator variables obesity (BMI) and circulating inflammatory proteins were introduced to investigate their mediating effects between sarcopenia and osteoarthritis. The main characteristic of patients with sarcopenia is the decrease in muscle mass, therefore ALM was chosen as the exposure factor for the mediator analysis. Then, considering that KOA is the most common type of osteoarthritis, the study analyzed KOA as the outcome factor. In the mediation analysis, UVMR analysis was first used to explore the causal relationship between circulating inflammatory proteins and KOA, screening out inflammatory proteins that have a causal relationship with KOA. Subsequently, the mediation effects of obesity and the selected inflammatory proteins between ALM and KOA were investigated. Traditionally Two-Step Method was utilized for analyzing the mediation effects. we used the “product of coefficients approach” to evaluate the indirect impact of ALM on KOA through each potential mediator [25]. The indirect effect and proportion were obtained by the delta method [26]. In the first step, the causal relationship between the exposure factor (ALM) and mediator variables (BMI, circulating inflammatory proteins) was assessed. Gene variants strongly associated with the exposure factor and not directly related to the mediator and outcome variables were selected as instrumental variables. The Inverse Variance Weighted (IVW) method was employed as the primary analytical method. In the second step, the causal relationship between mediator variables (BMI, circulating inflammatory proteins) and the outcome variable (KOA) was evaluated. Gene variants strongly associated with the mediator variables and not directly related to the outcome variable were selected as instrumental variables. Finally, a comprehensive analysis was conducted by combining the results of the first and second steps to estimate the mediation effects of the mediator variables (BMI, circulating inflammatory proteins) between the exposure factor (ALM) and the outcome variable (KOA). A flowchart of the study design is shown in Fig. 1.

### Data source

All data used in this study are available in the public domain without additional ethical approval. The ALM data came from a GWAS study published by Pei YF et al. ALM was quantified in 450,243 participants of the UK Biobanking cohort using the Tanita BC 418ma body fat analyzer and its measurement accuracy was verified using DEXA method [27]; the LHG data was obtained from a GWAS meta-analysis study published by Garan G et al., which included 256,523 European descendants aged 60 years or older. Grip strength was measured using the Jamar J00105 hydraulic hand dynamometer, with maximum grip strength recorded in kilograms. Low grip strength was defined as < 30 kg for males and < 20 kg for females [28]; UWP data were sourced from the publicly available UK BioBank database, containing 459,915 European participants. Participants were assessed based on their responses to the following question: "How do you classify your regular walking speed? " (Slow Pace was considered below 3 mph, moderate pace corresponded to 3–4 mph, and speeds exceeding 4 mph were classified as fast) [29].

BMI data came from a study published by Sakaue S et al. which included a total of 359,983 samples [30]; Circulating inflammatory protein data were sourced from a study published by Zhao JH et al. involving 14,824 participants measured for 91 plasma proteins using the linkTarget platform for genome-wide protein quantitative trait locus (pQTL) analysis. Catlog numbers for the 91 circulating inflammatory proteins were GCST90274758 ~ GCST90274848 [31]; Television viewing time data were obtained from the UK BioBank database, including 437,887 European participants. Participants were assessed based on their responses to the following question: "How many hours do you spend watching television in a day? " [29]; the insomnia data were also resourced from the UK Biobank database, comprising 462,341 samples. Participants were assessed based on their responses to the following question: " Do you have difficulty falling asleep or waking up at night?". Response options included "Never/Rarely", "Sometimes", "Usually", or "Prefer not to answer". Participants who answered " Prefer not to answer " were defined as missing [29]; FN-BMD data were derived from a GWAS meta-analysis conducted by the Genetic Factors for Osteoporosis (GEFOS), involving 32,735 samples [32].

The KOA and HOA data were sourced from the largest-scale genome-wide meta-analysis to date. Inclusion and exclusion criteria for KOA and HOA were based on ICD codes. KOA data included 62,497 cases and 333,557 controls, while HOA data included 36,445 cases and 316,943 controls [33] (Table 1).

**Table 1:** Data information used in the MR analyses for this study

Exposure or outcome	Sample size	GWAS ID or PMID
Appendicular lean mass	450,243	ebi-a-GCST90000025
Low hand grip	48,596 cases and 207,927 controls	ebi-a-GCST90007526
Usual walking pace	459,915	ukb-b-4711
Body mass index	359,983	ebi-a-GCST90018947
Circulating inflammatory proteins	14,824	PMID: 37,679,551
Insomnia	462,341	ukb-b-3957
Time spent watching television	437,887	ukb-b-5192
Bone mineral density	56,284	ieu-a-980
Knee osteoarthritis	62,497 cases and 33,3557 controls	PMID: 34,822,786
Hip osteoarthritis	36,445 cases and 316,943 controls	PMID: 34,822,786

### Criteria for IV selection

The SNPs included in this analysis were selected based on the following criteria. To screen highly correlated SNPs, all selected instrumental variables reached genome-wide significance (significance threshold: *P* < 5 × 10^–8^). To avoid the impact of linkage disequilibrium, SNPs that did not meet the associated criteria (r^2^ = 0.001; 10,000 kb distance) were removed using R software. The F value, which represents a criterion for the strength of the relationship between instrumental variables and exposure, for all selected SNPs was greater than 10 and SNPs with F < 10 were excluded. In this study, the F statistic was calculated by the following formula: F = β exposure^2^/SE exposure^2^ [34]. The remaining SNPs were detected by the Phenoscanner database (http://www.phenoscanner.medschl.cam.ac.uk/), and SNPs associated with KOA, HOA confounders (e.g., BMI, percent body fat) were manually removed based on the results of the analysis.

### Causal effect estimation and sensitivity analyses

In this study, the random-effects inverse-variance weighting (IVW) method, MR‒Egger method, weighted median method, simple mode, and weighted mode were used for the UVMR analysis [35–37].

Pleiotropy was tested by MR‒Egger regression. MR‒Egger regression analysis was used to detect whether the selected SNP had horizontal pleiotropy, and its regression intercept was used to evaluate the magnitude of pleiotropy. The null hypothesis of this hypothesis test is that the intercept term is zero, that is, there is no pleiotropy, and the rejection of the null hypothesis indicates that there is pleiotropy between the instrumental variables and the outcomes [36]. Heterogeneity between each SNP estimate was assessed using Cochran’s Q test, and if the results of the Q test were statistically significant and proved that the analysis results were significantly heterogeneous, we focused on the results of the random-effects IVW method [38]. The leave-one-out test was used to gradually eliminate each SNP, and the effect size change of the remaining SNPs was calculated [39]. If the results are greatly changed after excluding a single SNP, this SNP has a great impact on the results and is a sensitive SNP, so it needs to be eliminated, and MR analysis should be performed again. In addition, MR-PRESSO analysis was used to detect and remove outliers, and MR analysis was repeated after removing outliers [40].

To explore the independent causal associations of the three sarcopenia phenotypes with KOA and HOA, MVMR analysis was further performed using the IVW, MR‒Egger and weighted median methods. As sarcopenia is often accompanied by a loss of muscle mass and a decrease in physical function, in the MVMR analysis of this study, the sarcopenia phenotype combined with confounding factors was analyzed separately for patients with KOA and those with HOA. IVW was used as the primary method for the MVMR analysis. In addition, LASSO regression analysis was used to ensure the robustness of the results.

Because the outcome was a dichotomous variable, the MR analysis results needed to be converted into odds ratios (ORs) to evaluate the causal relationships of the sarcopenia phenotypes with KOA and HOA, and *P* < 0.05 was used as the threshold for determining statistical significance. In addition, to improve statistical rigor, this study applied False Discovery Rate (FDR) correction to adjust for multiple comparisons, thereby reducing the risk of Type I errors. Adjusted *P*-value of < 0.05 indicates a significant causal relationship. If the unadjusted *p*-value is < 0.05 but the FDR-adjusted *p*-value is > 0.05, it suggests a suggestive association [41].

## Results

### Results of MR analysis of sarcopenia phenotype with KOA and HOA

#### Results of UVMR analysis of sarcopenia phenotype with KOA and HOA

According to the screening criteria, in the UVMR analysis of the sarcopenia phenotype with KOA, a total of 504 SNPs were included in the analysis of ALM with KOA for analysis, with an F-statistic range of 29.75–708.02; 4 SNPs were included in the analysis of LHG with KOA for analysis, with an F-statistic range of 29.55–39.69; 14 SNPs were included in the analysis of UWP with KOA for analysis The F-statistic range was 29.79–42.61. In the UVMR analyses of the sarcopenia phenotype with HOA, the analyses of ALM with HOA incorporated 502 SNPs for analysis, with an F-statistic range of 29.75–609.31; and the analyses of LHG with HOA incorporated 10 SNPs for analysis, with an F-statistic range of 29.55–50.80; The analysis of UWP versus HOA included 16 SNPs for analysis, with an F-statistic range of 29.79–42.61. In this study, the F-statistic for each instrumental variable was > 10, indicating that the instrumental variables used in this study had a low bias. (Supplementary Material 1).

In the UVMR IVW analysis of ALM and KOA revealed a causal relationship between those variables (OR = 1.151, 95% CI: 1.087–1.218, *P* = 1.19 × 10^–6^, P_FDR_ = 7.14 × 10^–6^), and the weighted median method was consistent with the IVW results. UVMR analysis of LHG and KOA revealed a causal relationship between the two variables (OR = 1.215, 95% CI: 1.004–1.470; *P* = 0.046, P_FDR_ = 0.055). UVMR analysis of UWP and KOA revealed a causal relationship between those variables (OR = 0.503, 95% CI: 0.292–0.867; *P* = 0.013, P_FDR_ = 0.027).

The UVMR IVW analysis revealed a causal relationship between ALM and HOA (OR = 1.181, 95% CI: 1.103–1.265, *P* = 2.05 × 10^–6^, P_FDR_ = 6.15 × 10^–6^). The results of the weighted median, simple mode, and weighted mode analyses were consistent with the IVW results. The UVMR IVW analysis revealed no causal relationship between LHG and HOA (OR = 1.206, 95% CI: 0.909–1.600, *P* = 0.194, P_FDR_ = 0.194) and a causal relationship between UWP and HOA (OR = 0.438, 95% CI: 0.226–0.849, *P* = 0.014, P_FDR_ = 0.022) (Table 2).

**Table 2:** Results of univariate mendelian randomization analyses on sarcopenia phenotypes and osteoarthritis in knee and hip joints

Outcome	Exposure	Method	SNP(n)	β	SE	OR (95%CI)	*P*- value
KOA	ALM	MR-Egger	503	0.142	0.075	1.152(0.995–1.335)	0.059
		Weighted median method	503	0.124	0.034	1.132(1.059- 1.210)	2.59 × 10^–4^
		Inverse variance weighting	503	0.140	0.029	1.151(1.087- 1.218)	1.19 × 10^–6^
		Simple model	503	-0.062	0.130	0.940(0.728- 1.214)	0.635
		Weighted model	503	0.176	0.115	1.193(0.953- 1.493)	0.125
	LHG	MR-Egger	4	-0.001	0.231	0.999(0.635- 1.573)	0.997
		Weighted median method	4	0.114	0.105	1.121(0.913- 1.376)	0.276
		Inverse variance weighting	4	0.195	0.097	1.215(1.004- 1.470)	0.046
		Simple model	4	0.067	0.146	1.070(0.803- 1.425)	0.676
		Weighted model	4	0.067	0.152	1.070(0.794- 1.442)	0.688
	UWP	MR-Egger	14	-1.172	1.253	0.310(0.027- 3.611)	0.368
		Weighted median method	14	-0.670	0.375	0.512(0.246- 1.067)	0.074
		Inverse variance weighting	14	-0.687	0.278	0.503(0.292- 0.867)	0.013
		Simple model	14	0.240	0.820	1.271(0.255- 6.349)	0.774
		Weighted model	14	0.265	0.736	1.303(0.308- 5.508)	0.725
HOA	ALM	MR-Egger	500	0.100	0.094	1.105(0.919- 1.327)	0.288
		Weighted median method	500	0.185	0.042	1.203(1.108- 1.307)	1.14 × 10^–5^
		Inverse variance weighting	500	0.166	0.035	1.181(1.103- 1.265)	2.05 × 10^–6^
		Simple model	500	0.346	0.146	1.414(1.062- 1.881)	0.018
		Weighted model	500	0.292	0.112	1.339(1.076- 1.667)	0.009
	LHG	MR-Egger	10	0.810	0.451	2.248(0.928- 5.442)	0.110
		Weighted median method	10	0.059	0.101	1.061(0.870- 1.294)	0.561
		Inverse variance weighting	10	0.187	0.144	1.206(0.909- 1.600)	0.194
		Simple model	10	0.078	0.171	1.082(0.774- 1.512)	0.657
		Weighted model	10	0.078	0.168	1.082(0.778- 1.503)	0.651
	UWP	MR-Egger	16	-2.496	1.367	0.082(0.006- 1.202)	0.089
		Weighted median method	16	-0.825	0.449	0.438(0.182- 1.056)	0.066
		Inverse variance weighting	16	-0.825	0.337	0.438(0.226- 0.849)	0.014
		Simple model	16	-0.769	0.812	0.463(0.094- 2.278)	0.359
		Weighted model	16	-0.719	0.813	0.487(0.099- 2.396)	0.390

*KOA* Knee osteoarthritis, *HOA *Hip osteoarthritis,  *ALM *Appendicular lean mass, *LHG* Low hand grip, *UWP *Usual walking pace

None of the MR‒Egger pleiotropy tests in the UVMR analysis detected potential horizontal pleiotropy, indicating that instrumental variables did not significantly affect outcomes through pathways other than exposure. According to the Cochran Q test, the analysis results of ALM with KOA, HOA and UWP with HOA showed significant heterogeneity, so the random-effects IVW was used as the main analysis method in this study. (Table 3) Leave-one-out cross-validation analysis revealed that no single-nucleotide polymorphisms affected the overall causal estimation. (Supplementary Material 2) Pleiotropy was found in the MR-PRESSO analysis of ALM and the two outcome factors, but the results still showed statistical significance after excluding abnormal SNPs.

**Table 3:** Results of MR‒Egger pleiotropy test and heterogeneity test of univariate Mendelian randomization analysis

Outcome	Exposure	Pleiotropy test	Heterogeneity test	
		*P*-value	MR Egger	IVW
KOA	ALM	0.981	2.15 × 10^–55^	3.30 × 10^–55^
	LHG	0.448	0.226	0.233
	UWP	0.698	0.228	0.280
HOA	ALM	0.442	5.84 × 10^–42^	6.11 × 10^–42^
	LHG	0.186	9.66 × 10^–6^	3.58 × 10^–7^
	UWP	0.228	0.247	0.208

*KOA* Knee osteoarthritis, *HOA* Hip osteoarthritis, *ALM* Appendicular lean mass, *LHG* Low hand grip, *UWP* Usual walking pace, *IVW* Inverse variance weighting

### Results of MVMR analysis of sarcopenia phenotype with KOA and HOA

After adjusting for confounding factors, MVMR analysis of KOA revealed a causal relationship between the three sarcopenia phenotypes and KOA (ALM: OR = 1.323, 95%CI: 1.224- 1.431, *P* = 2.07 × 10^–12^) (LHG: OR = 1.161, 95%CI: 1.044- 1.292, *P* = 0.006) (UWP: OR = 0.511, 95%CI: 0.290- 0.899, *P* = 0.020). Since the results of the UVMR analysis of the association between LHG and HOA showed that there was no causal relationship between them, only ALM and UWP combined with confounding factors were included in the MVMR analysis in this study. In the MVMR IVW analysis, there was a direct causal effect of ALM on HOA (ALM: OR = 1.245, 95%CI: 1.149- 1.348, *P* = 7.65 × 10^–8^), but there was no causal effect of UWP on HOA (UWP: OR = 0.825, 95%CI: 0.419- 1.626, *P* = 0.579). There was no causal relationship between UWP and HOA after adjustment for confounders or internal adjustment between sarcopenia phenotypes. In addition, the MR-LASSO results were consistent with the above results, indicating the robustness of the results. (Table 4).

**Table 4:** Results of multivariable mendelian randomization analyses on sarcopenia phenotypes and osteoarthritis in knee and hip joints

Outcome	Exposure	Method	OR (95%CI)	*P*-value
KOA	ALM	MR-Egger	1.273(1.130- 1.433)	6.69 × 10^–5^
		Weighted median method	1.327(1.219- 1.444)	6.13 × 10^–11^
		Inverse variance weighting	1.323(1.224- 1.431)	2.07 × 10^–12^
		MR-Lasso	1.325(1.250- 1.405)	2.90 × 10^–21^
	LHG	MR-Egger	1.159(1.042- 1.290)	0.007
		Weighted median method	1.182(1.061- 1.317)	0.002
		Inverse variance weighting	1.161(1.044- 1.292)	0.006
		MR-Lasso	1.194(1.100- 1.295)	2.11 × 10^–5^
	UWP	MR-Egger	0.497(0.281- 0.878)	0.016
		Weighted median method	0.534(0.303- 0.940)	0.030
		Inverse variance weighting	0.511(0.290- 0.899)	0.020
		MR-Lasso	0.527(0.350- 0.794)	0.002
HOA	ALM	MR-Egger	1.097(0.960- 1.253)	0.175
		Weighted median method	1.248(1.143- 1.364)	8.62 × 10^–7^
		Inverse variance weighting	1.245(1.149- 1.348)	7.65 × 10^–8^
		MR-Lasso	1.234(1.162- 1.311)	8.82 × 10^–12^
	UWP	MR-Egger	0.762(0.387- 1.502)	0.432
		Weighted median method	0.817(0.425- 1.571)	0.545
		Inverse variance weighting	0.825(0.419- 1.626)	0.579
		MR-Lasso	0.918(0.562- 1.499)	0.732

*KOA* Knee osteoarthritis, *HOA* Hip osteoarthritis, *ALM* Appendicular lean mass, *LHG* Low hand grip, *UWP* Usual walking pace

### Mediator analysis

#### Results of UVMR analysis of circulating inflammatory proteins with KOA

In the UVMR analysis of circulating inflammatory proteins with KOA, the IVW analysis results showed that C-C motif chemokine 23 levels (CCL23) (OR= 0.959, 95%CI: 0.923- 0.995, *P*= 0.028, P_FDR_= 0.399), Fibroblast growth factor 19 levels (FGF19) (OR= 1.079, 95%CI: 1.010- 1.153, *P*= 0.025, P_FDR_= 0.399), Latency-associated peptide transforming growth factor β 1 levels (LAP TGF-β1) (OR= 0.839, 95%CI: 0.761- 0.924, *P*= 3.89×10^-4^, P_FDR_= 0.023), and Leukemia inhibitory factor receptor levels(LIFR) (OR=0.933, 95% CI: 0.883-0.985, *P*=3.89×10-4, P_FDR_=0.023) were causally associated with KOA. (See Supplementary Material 3 for details of the results of the analysis of 91 circulating inflammatory proteins with KOA).

### Two-step MR analysis

According to the two-step mediation analysis method, the potential mediation effects of BMI, CCL23, FGF19, LAP-TGF-β1, and LIFR were assessed in the previously identified promotion of KOA risk by ALM. Testing the mediation effect of BMI in the relationship between ALM and KOA yielded a *p*-value of 0.049, with the proportion of mediated effect (IE_div_TE) at 0.059 and a confidence interval of the proportion of mediated effect ranging from 7.083×10-5 to 0.119, indicating the existence of a mediation effect. Testing the mediating effect of CCL23 in the relationship between ALM and KOA resulted in a *p*-value of 0.240, IE_div_TE of -0.009, and a confidence interval for the proportion of mediated effect ranging from -0.024 to 0.007, indicating no mediating effect. Testing the mediating effect of FGF19 in the relationship between ALM and KOA yielded a p-value of 0.257, IE_div_TE of -0.015, and a confidence interval for the proportion of mediated effect ranging from -0.043 to 0.013, indicating no mediating effect. Testing the mediating effect of LAP-TGF-β1 in the relationship between ALM and KOA yielded a *p*-value of 0.606, IE_div_TE of 0.013, and a confidence interval for the proportion of mediated effect ranging from -0.041 to 0.068, indicating no mediating effect. Testing the mediating effect of LIFR in the relationship between ALM and KOA resulted in a *p*-value of 0.128, IE_div_TE of -0.022, and a confidence interval for the proportion of mediated effect ranging from -0.052 to 0.008, indicating no mediating effect. (See Table 5 for analysis results of exposure with outcome, exposure with mediator, and mediator with outcome. See Table 6 for analysis results of total effect, direct effect, and mediated effect in the two-step MR analysis).

**Table 5:** The causal relationship between exposure and outcome, exposure and mediation, mediation and outcome

Exposure	Outcome	Method	β	SE	OR (95%CI)	*P*- value
ALM	KOA	IVW	0.184	0.025	1.202(1.145- 1.262)	1.18 × 10^–13^
ALM	BMI	IVW	0.018	0.009	1.018(1.000- 1.036)	0.048
BMI	KOA	IVW	0.610	0.038	1.841(1.710- 1.981)	1.88 × 10^–59^
ALM	KOA	IVW	0.184	0.025	1.202(1.145- 1.262)	1.18 × 10^–13^
ALM	CCL23	IVW	0.038	0.028	1.039(0.984- 1.097)	0.165
CCL23	KOA	IVW	-0.042	0.019	0.959(0.923- 0.995)	0.028
ALM	KOA	IVW	0.184	0.025	1.202(1.145- 1.262)	1.18 × 10^–13^
ALM	FGF19	IVW	-0.038	0.028	0.964(0.913- 1.018)	0.188
FGF19	KOA	IVW	0.076	0.034	1.079(1.010- 1.153)	0.025
ALM	KOA	IVW	0.184	0.025	1.202(1.145- 1.262)	1.18 × 10^–13^
ALM	LAP-TGF-β1	IVW	-0.014	0.028	0.988(0.934- 1.041)	0.602
LAP-TGF-β1	KOA	IVW	-0.176	0.050	0.839(0.761- 0.924)	3.89 × 10^–4^
ALM	KOA	IVW	0.184	0.025	1.202(1.145- 1.262)	1.18 × 10^–13^
ALM	LIFR	IVW	0.058	0.030	1.060(0.999- 1.126)	0.055
LIFR	KOA	IVW	-0.069	0.028	0.933(0.883- 0.985)	0.013

*KOA* Knee osteoarthritis, *ALM* Appendicular lean mass, *BMI* Body mass index, *IVW* Inverse variance weighting, *CCL23* C–C motif chemokine 23 levels, *FGF19* Fibroblast growth factor 19 level, *LAP-TGF-β1* Latency-associated peptide transforming growth factor β 1 levels, *LIFR* Leukemia inhibitory factor receptor levels

**Table 6:** The total effect, direct effect and intermediary effect mediated by BMI

Exposure	Mediator	Outcome	OR of TE (95% CI)	OR of DE (95% CI)	OR of IE (95% Cl)	Mediation effect proportion (95% Cl)
ALM	BMI	KOA	1.202(1.145- 1.262)	1.189(1.131- 1.249)	1.011(1.000- 1.022)	0.059(7.083 × 10–5- 0.119)

*KOA* Knee osteoarthritis, *ALM* Appendicular lean mass, *BMI* Body mass index, *IVW* Inverse variance weighting, *TE* total effect, *DE *Direct effect, *IE *Intermediary effect

## Discussion

Amidst the global aging population, sarcopenia, as a degenerative disease of old age, is gradually increasing in incidence. The relationship between sarcopenia and arthritis has drawn increasing attention. Previous MR studies on sarcopenia and osteoarthritis mainly employed UVMR analysis [42–44], and they found a positive causal relationship between ALM and KOA, as well as HOA, and a negative causal relationship between UWP and KOA, as well as HOA, consistent with the findings of this study. Furthermore, the causal relationship between LHG and KOA was also found in the UVMR analysis of the present study. Considering that UVMR analysis cannot fully eliminate the effect of horizontal pleiotropy, MVMR was able to efficiently remove the effect of horizontal pleiotropy to access the independent causal relationship of exposure factors on outcome factors. Based on UVMR, this study further used MVMR for analysis. By adjusting for BMI, insomnia, television viewing time and FN-BMD, it investigated the independent causal relationship of sarcopenia phenotype on KOA and HOA. The results of the adjusted MVMR analysis indicated that there was no causal relationship between UWP and HOA, which might be due to the confounding effect modification in the UVMR analysis and the internal effect modification among the sarcopenia phenotypes masking the relationship between UWP and HOA. The findings of this study suggest that ALM, LHG, and UWP have causal relationships with KOA, and ALM has a causal relationship with HOA. In addition, the results of two-step MR analysis indicate that BMI plays a mediating role in the causal relationship between ALM and KOA.

ALM refers to the mass of body appendage muscles and bones, and in sarcopenia, a decrease in ALM mainly manifests as a decrease in appendage muscle mass. The results of this study suggest that the reduction of ALM increases the risk of KOA and HOA. Previous observational studies and animal experiments support our conclusion [45–50]. In the aspect of muscle function, the study shows that a decrease in grip strength and walking speed will increase the risk of KOA. Andrews J S et al. found in a European population that for every 1 standard deviation decrease in grip strength, the risk of KOA increases by 1.2 times [15]. Similarly, decreased grip strength has been found to be strongly associated with the development of osteoarthritis in East Asian populations [51]. Studies on the grip strength of elderly people have also shown that the grip strength of elderly people is positively correlated with the strength of other muscles in the whole body [52]. Therefore, a decrease in grip strength can indirectly reflect the deterioration of lower-limb muscles. As one of the most important muscles for lower-limb movement, the quadriceps femoris is crucial to the influence of KOA [53], and a decrease in strength will lead to increased pressure on the knee joint and then accelerate the progression of cartilage tissue degradation and arthritis [54]. In addition, walking speed can reflect the balance ability and nervous system function of the elderly, and is widely used to evaluate the overall physical function of the elderly. The results of this study suggest that a decrease in walking speed leads to an increased risk of KOA, but does not affect the occurrence and development of HOA. Previous observational studies have similarly found a negative correlation between walking speed and KOA, while walking speed was not associated with the development of HOA [55]. In the study of gait characteristics of sarcopenia patients, it has been found that the time of the support phase in the gait cycle of patients is significantly increased, and the delay of the support phase will lead to the joint bearing longer gravity and ground reaction force, thus increasing the risk of joint soft tissue injury [56].

The results of this study suggest that obesity plays a mediator role between the decline in ALM and the onset of KOA. The reduction of skeletal muscle in elderly individuals may trigger obesity through multiple mechanisms, including chronic inflammation, hormonal metabolic disorders, and decreased basal metabolic rate [57]. Additionally, obesity has long been recognized as one of the risk factors for KOA, as confirmed in the study conducted by Wang et al., which also found a positive causal relationship between obesity and KOA [58], consistent with the findings of this study. Obesity increases load on knee joint cartilage tissue and changes in body composition may also lead to metabolic disorders, thereby inducing the occurrence arthritis [59]. For sarcopenic patients, it is important to reduce the incidence of KOA by lowering BMI. It should be emphasized that during the process of reducing BMI, adipose tissue should be reduced as much as possible in sarcopenic patients while preserving muscle mass. The results of this study provide some new insights into the pathogenesis of KOA involving circulating inflammatory proteins. Although circulating inflammatory proteins may not mediate the occurrence of osteoarthritis resulting from skeletal muscle loss, this study found that high levels of CCL23, LAP-TGF-β1, and LIFR might contribute to reduce the risk of KOA, while high levels of FGF19 might increase the risk of KOA. These findings provide new research directions and insights for future studies on the interaction between circulating inflammatory proteins and osteoarthritis.

This study provides potential evidence that the sarcopenia phenotype contributes to the onset or development of KOA and HOA. Compared with observational studies, MR analysis has the advantage of avoiding reverse causation and confounding factors. However, this study has several limitations. First, the GWAS data used in this study came from a European database and were limited to individuals of European ancestry; it is unknown whether these results apply to other ethnic groups. Second, due to the current lack of GWAS data on the phenotype of sarcopenia itself, this study chose ALM, LHG and UWP to replace sarcopenia in exploring its causal relationships with KOA and HOA.

In conclusion, this study suggests that in a European population, the loss of appendicular lean body mass in patients with sarcopenia has a pathogenic effect on KOA and HOA, and a decrease in grip strength and walking speed in patients with sarcopenia may increase the risk of KOA. Meanwhile, obesity plays a mediator role in the occurrence of KOA due to appendicular lean body mass reduction.

### Supplementary Information

Supplementary Material 1.

Supplementary Material 2.

Supplementary Material 3.

## Data Availability

Data used in the present study are all publicly available. The Usual walking pace, insomnia and TV watching time data can be found at http://www.nealelab.is/uk-biobank/; Appendicular lean mass, Low hand grip and femoral-neck bone mineral density data can be found at https://gwas.mrcieu.ac.uk/. Knee osteoarthritis and Hip osteoarthritis data can be found at https://msk.hugeamp.org/downloads.html. All the data generated by the MR analysis is in the included Supplementary Material 1.
