# Supplementary material for: Causal relationship between sarcopenia with osteoarthritis and the mediating role of obesity: a univariate, multivariate, two-step Mendelian randomization study
Source: BMC Geriatr. 2024 May 29;24:469. doi: 10.1186/s12877-024-05098-8 (PMC11138082; doi:10.1186/s12877-024-05098-8)
Supplement: Supplementary file 1 — Supplementary Material 1. [file 12877_2024_5098_MOESM1_ESM.docx]

**Supplementary Material 1**

**(1) Supplementary Table 1** Genome-wide significant SNPs for the Association between Appendicular lean mass as exposure and Knee osteoarthritis as outcome 2

**(2) Supplementary Table 2** Genome-wide significant SNPs for the Association between Low hand grip as exposure and Knee osteoarthritis as outcome 20

**(3) Supplementary Table 3** Genome-wide significant SNPs for the Association between Usual walking pace as exposure and Knee osteoarthritis as outcome 21

**(4) Supplementary Table 4** Genome-wide significant SNPs for the Association between Appendicular lean mass as exposure and Hip osteoarthritis as outcome 22

**(5) Supplementary Table 5** Genome-wide significant SNPs for the Association between Low hand grip as exposure and Hip osteoarthritis as outcome 40

**(6) Supplementary Table 6** Genome-wide significant SNPs for the Association between Usual walking pace as exposure and Hip osteoarthritis as outcome 41

**Supplementary Table 1** Genome-wide significant SNPs for the Association between Appendicular lean mass as exposure and Knee osteoarthritis as outcome

| SNP | alt | ref | beta. exposure | beta. outcome | se. exposure | se. outcome | pval. exposure | pval. outcome | F |
| --- | --- | --- | --- | --- | --- | --- | --- | --- | --- |
| rs10008637 | T | C | 0.013 | -0.0033 | 0.0019 | 0.007130344 | 7.80E-12 | 0.6435 | 46.81440443 |
| rs10019221 | T | G | -0.0124 | -0.0127 | 0.0019 | 0.007559355 | 6.74E-11 | 0.09295 | 42.59279778 |
| rs10040039 | A | G | 0.0163 | 0.0216 | 0.0019 | 0.007257463 | 9.57E-18 | 0.002918 | 73.59833795 |
| rs10041978 | A | G | -0.0172 | -0.0064 | 0.0019 | 0.007364057 | 1.40E-19 | 0.3848 | 81.9501385 |
| rs1004982 | T | C | 0.0117 | -0.0256 | 0.002 | 0.007334754 | 4.92E-09 | 0.0004826 | 34.2225 |
| rs10058744 | A | G | 0.0146 | 0.0246 | 0.0019 | 0.007048017 | 1.54E-14 | 0.0004824 | 59.04709141 |
| rs10099846 | T | C | 0.0116 | -0.0038 | 0.002 | 0.007531996 | 6.63E-09 | 0.6139 | 33.64 |
| rs10112506 | A | G | 0.012 | 0.0161 | 0.0019 | 0.007312225 | 2.69E-10 | 0.02768 | 39.88919668 |
| rs10119967 | A | C | -0.0353 | -0.0018 | 0.0023 | 0.00880952 | 3.66E-53 | 0.8381 | 235.5557656 |
| rs10128781 | T | C | -0.0172 | -0.0133 | 0.0021 | 0.007805873 | 2.60E-16 | 0.08841 | 67.08390023 |
| rs1014526 | T | C | 0.0138 | 0.0197 | 0.002 | 0.007221824 | 5.20E-12 | 0.006375 | 47.61 |
| rs10176654 | T | C | 0.0128 | 0.0161 | 0.0023 | 0.008681662 | 2.62E-08 | 0.06367 | 30.97164461 |
| rs10202701 | T | C | 0.0227 | -0.0123 | 0.0019 | 0.007052078 | 6.70E-33 | 0.08113 | 142.7396122 |
| rs10209278 | T | C | 0.0141 | -0.0064 | 0.002 | 0.007453032 | 1.79E-12 | 0.3905 | 49.7025 |
| rs10222594 | A | G | 0.0115 | -0.0047 | 0.002 | 0.007954221 | 8.92E-09 | 0.5546 | 33.0625 |
| rs10241451 | T | C | -0.0145 | 0.0012 | 0.0023 | 0.008644887 | 2.89E-10 | 0.8896 | 39.74480151 |
| rs10283100 | A | G | -0.0575 | -0.0199 | 0.0041 | 0.015472854 | 1.11E-44 | 0.1984 | 196.6835217 |
| rs1035583 | A | G | 0.0148 | 0.01 | 0.0019 | 0.007224437 | 6.73E-15 | 0.1663 | 60.67590028 |
| rs1040977 | T | C | -0.024 | -0.0257 | 0.0025 | 0.009620975 | 7.99E-22 | 0.007557 | 92.16 |
| rs10421750 | T | G | -0.0145 | 0.0129 | 0.0021 | 0.011503053 | 5.03E-12 | 0.2621 | 47.67573696 |
| rs10491967 | A | G | 0.0471 | 0.0097 | 0.0032 | 0.011535163 | 4.89E-49 | 0.4004 | 216.6416016 |
| rs10514518 | T | C | 0.0171 | -0.0012 | 0.002 | 0.007591473 | 1.23E-17 | 0.8744 | 73.1025 |
| rs1051952 | A | C | -0.0133 | 0.0221 | 0.0019 | 0.007768769 | 2.56E-12 | 0.004445 | 49 |
| rs1056747 | A | G | 0.0155 | 0.0143 | 0.0019 | 0.007142037 | 3.41E-16 | 0.04526 | 66.55124654 |
| rs1063582 | T | G | 0.0185 | 0.0171 | 0.0022 | 0.008365388 | 4.13E-17 | 0.04094 | 70.71280992 |
| rs10736029 | T | G | -0.0193 | 0.0062 | 0.0035 | 0.015906451 | 3.50E-08 | 0.6967 | 30.40734694 |
| rs10748128 | T | G | 0.0255 | 0.0274 | 0.002 | 0.007367789 | 3.12E-37 | 0.0002001 | 162.5625 |
| rs10749157 | T | C | -0.0113 | -0.0084 | 0.002 | 0.007730316 | 1.60E-08 | 0.2772 | 31.9225 |
| rs10764692 | A | C | 0.0111 | 0.0035 | 0.002 | 0.007314645 | 2.86E-08 | 0.6323 | 30.8025 |
| rs10776560 | T | C | -0.0157 | -0.0054 | 0.0019 | 0.007070124 | 1.42E-16 | 0.445 | 68.27977839 |
| rs10779958 | A | C | -0.0157 | -0.0218 | 0.0026 | 0.009979679 | 1.56E-09 | 0.02893 | 36.46301775 |
| rs10796828 | T | G | -0.0154 | -0.0019 | 0.002 | 0.007720243 | 1.36E-14 | 0.8056 | 59.29 |
| rs10810474 | T | C | 0.0143 | -0.0062 | 0.0019 | 0.007198833 | 5.22E-14 | 0.3891 | 56.64542936 |
| rs10815304 | T | C | -0.0151 | 0.0091 | 0.0023 | 0.0088074 | 5.20E-11 | 0.3015 | 43.1020794 |
| rs10822117 | A | G | 0.0176 | -0.0163 | 0.0022 | 0.008373119 | 1.24E-15 | 0.05157 | 64 |
| rs10827415 | T | G | 0.0142 | -0.0209 | 0.0021 | 0.007952834 | 1.36E-11 | 0.008589 | 45.72335601 |
| rs10832963 | T | G | 0.0203 | 0.0015 | 0.0022 | 0.007942255 | 2.78E-20 | 0.8502 | 85.14256198 |
| rs10840399 | T | C | -0.0209 | 0.0219 | 0.0031 | 0.011571793 | 1.56E-11 | 0.05842 | 45.45369407 |
| rs10849576 | T | G | 0.0117 | 0.0115 | 0.0019 | 0.007173346 | 7.37E-10 | 0.1089 | 37.91966759 |
| rs10880272 | T | G | 0.0138 | 0.0053 | 0.0019 | 0.007207028 | 3.78E-13 | 0.4621 | 52.7534626 |
| rs10883555 | T | C | 0.0254 | 0.0056 | 0.0019 | 0.007408238 | 9.25E-41 | 0.4497 | 178.7146814 |
| rs10917335 | A | G | 0.0198 | 0.0218 | 0.002 | 0.011320472 | 4.16E-23 | 0.05414 | 98.01 |
| rs10922476 | A | G | -0.016 | -0.0145 | 0.0019 | 0.007135006 | 3.73E-17 | 0.04213 | 70.91412742 |
| rs1093086 | T | C | -0.0129 | -0.0093 | 0.0022 | 0.008199428 | 4.53E-09 | 0.2567 | 34.3822314 |
| rs10995566 | T | C | -0.0123 | 0.0079 | 0.002 | 0.007685622 | 7.75E-10 | 0.304 | 37.8225 |
| rs11021305 | T | C | -0.0152 | 0.0098 | 0.0019 | 0.007309314 | 1.24E-15 | 0.18 | 64 |
| rs11042717 | T | C | 0.029 | -0.0248 | 0.0019 | 0.007101133 | 1.35E-52 | 0.0004787 | 232.9639889 |
| rs11132166 | T | C | 0.0292 | -0.0039 | 0.005 | 0.019099256 | 5.22E-09 | 0.8382 | 34.1056 |
| rs11158820 | A | G | 0.0235 | -0.0063 | 0.0021 | 0.007780449 | 4.54E-29 | 0.4181 | 125.2267574 |
| rs111622870 | T | C | 0.0282 | 0.0487 | 0.0044 | 0.017473281 | 1.46E-10 | 0.005318 | 41.07644628 |
| rs11175919 | A | G | 0.0349 | -0.0118 | 0.0059 | 0.021539034 | 3.31E-09 | 0.5838 | 34.99023269 |
| rs11187838 | A | G | 0.0394 | 0.0034 | 0.0019 | 0.007025082 | 1.61E-95 | 0.6284 | 430.0166205 |
| rs111901094 | T | G | -0.0253 | -0.0033 | 0.0025 | 0.009783931 | 4.50E-24 | 0.7359 | 102.4144 |
| rs11191208 | A | G | 0.0147 | 0.009 | 0.0024 | 0.009195335 | 9.07E-10 | 0.3277 | 37.515625 |
| rs11198591 | A | G | 0.0148 | 0.0059 | 0.002 | 0.007562606 | 1.36E-13 | 0.4353 | 54.76 |
| rs11210229 | A | G | -0.0125 | -0.0055 | 0.0019 | 0.007194729 | 4.74E-11 | 0.4446 | 43.28254848 |
| rs112369231 | T | C | -0.0181 | -0.0068 | 0.0021 | 0.010843732 | 6.75E-18 | 0.5306 | 74.28798186 |
| rs112373502 | T | C | -0.0179 | -0.0281 | 0.0028 | 0.01007903 | 1.63E-10 | 0.005304 | 40.86862245 |
| rs112537273 | T | C | 0.0212 | -0.0012 | 0.0022 | 0.008482466 | 5.61E-22 | 0.8875 | 92.85950413 |
| rs112873218 | T | C | 0.0216 | 0.0033 | 0.0031 | 0.011502847 | 3.22E-12 | 0.7742 | 48.54942768 |
| rs113107560 | T | G | 0.019 | 0.0295 | 0.0019 | 0.011055433 | 1.52E-23 | 7.62E-03 | 100 |
| rs113289555 | T | G | -0.0206 | -0.0207 | 0.0023 | 0.012468966 | 3.35E-19 | 0.09689 | 80.21928166 |
| rs113671109 | T | C | 0.015 | 0.0061 | 0.0023 | 0.008542891 | 6.95E-11 | 0.4752 | 42.53308129 |
| rs113827862 | T | C | 0.0235 | -0.0023 | 0.004 | 0.014897064 | 4.23E-09 | 0.8773 | 34.515625 |
| rs113852999 | A | G | 0.0246 | 0.0065 | 0.0025 | 0.009439986 | 7.57E-23 | 0.4911 | 96.8256 |
| rs114018835 | T | C | 0.0303 | 0.0032 | 0.0051 | 0.019955496 | 2.83E-09 | 0.8726 | 35.29757785 |
| rs114192718 | T | C | -0.0215 | -0.0209 | 0.0036 | 0.014029558 | 2.34E-09 | 0.1363 | 35.66743827 |
| rs114299654 | T | C | 0.0304 | 0.001 | 0.0051 | 0.021556716 | 2.51E-09 | 0.963 | 35.53094963 |
| rs115233595 | T | C | 0.0264 | -0.0117 | 0.0039 | 0.014335159 | 1.29E-11 | 0.4144 | 45.82248521 |
| rs115912456 | A | G | -0.0577 | 0.0272 | 0.0047 | 0.017650086 | 1.21E-34 | 0.1233 | 150.7148031 |
| rs116008080 | A | G | -0.0415 | 0.0152 | 0.0063 | 0.023368349 | 4.48E-11 | 0.5154 | 43.3925422 |
| rs11605297 | A | G | 0.0146 | -0.0005 | 0.0022 | 0.00778649 | 3.22E-11 | 0.9488 | 44.04132231 |
| rs11633371 | T | G | 0.0216 | 0.0112 | 0.0019 | 0.00703351 | 6.01E-30 | 0.1113 | 129.2409972 |
| rs116339650 | A | G | 0.0175 | -0.0076 | 0.0029 | 0.010836625 | 1.59E-09 | 0.4831 | 36.41498216 |
| rs116493405 | A | G | 0.0287 | -0.0017 | 0.0042 | 0.015191674 | 8.30E-12 | 0.9109 | 46.69444444 |
| rs11651280 | T | G | 0.0267 | -0.0125 | 0.004 | 0.014928178 | 2.47E-11 | 0.4024 | 44.555625 |
| rs11672848 | T | C | -0.0171 | 0.0118 | 0.0019 | 0.008979275 | 2.26E-19 | 0.1888 | 81 |
| rs11684531 | A | G | 0.0172 | 0.0061 | 0.0028 | 0.013385766 | 8.11E-10 | 0.6486 | 37.73469388 |
| rs11689546 | A | G | 0.0238 | 0.009 | 0.0019 | 0.009556267 | 5.36E-36 | 0.3463 | 156.9085873 |
| rs117068593 | T | C | 0.0403 | -0.0189 | 0.0024 | 0.009675345 | 2.81E-63 | 0.05077 | 281.9600694 |
| rs117203652 | A | G | -0.0346 | -0.0159 | 0.0055 | 0.022874793 | 3.16E-10 | 0.487 | 39.57553719 |
| rs11720869 | A | G | 0.0141 | -0.0049 | 0.002 | 0.007492145 | 1.79E-12 | 0.5131 | 49.7025 |
| rs11721522 | A | G | -0.0106 | 0.0023 | 0.0019 | 0.007239158 | 2.42E-08 | 0.7507 | 31.12465374 |
| rs1177765 | T | C | 0.0232 | 0.0074 | 0.0019 | 0.006999327 | 2.73E-34 | 0.2904 | 149.0969529 |
| rs11777835 | T | C | 0.0152 | -0.0002 | 0.0019 | 0.006967467 | 1.24E-15 | 0.9771 | 64 |
| rs117818446 | A | G | 0.0423 | 0.0392 | 0.0068 | 0.026548621 | 4.95E-10 | 0.1398 | 38.69571799 |
| rs11927331 | A | C | -0.0147 | -0.0001 | 0.002 | 0.009170905 | 1.98E-13 | 0.9913 | 54.0225 |
| rs11991823 | A | G | -0.0161 | 0.0023 | 0.002 | 0.010165342 | 8.28E-16 | 0.821 | 64.8025 |
| rs12037677 | T | C | 0.0178 | -0.0074 | 0.0021 | 0.009763588 | 2.33E-17 | 0.4485 | 71.84580499 |
| rs12055045 | T | C | 0.0213 | 0.0009 | 0.0023 | 0.008795644 | 2.03E-20 | 0.9185 | 85.7637051 |
| rs1211575 | A | G | -0.0237 | -0.019 | 0.0021 | 0.008487334 | 1.54E-29 | 0.02518 | 127.3673469 |
| rs1216743 | A | G | -0.0152 | -0.0015 | 0.0021 | 0.007942255 | 4.55E-13 | 0.8502 | 52.39002268 |
| rs12212816 | T | C | 0.0162 | 0.0111 | 0.0019 | 0.00706009 | 1.51E-17 | 0.1159 | 72.69806094 |
| rs12299065 | A | G | -0.024 | 0.0022 | 0.0028 | 0.010308351 | 1.02E-17 | 0.831 | 73.46938776 |
| rs12325539 | T | C | -0.0275 | -0.0288 | 0.0019 | 0.007208256 | 1.78E-47 | 0.0000646 | 209.4875346 |
| rs12340775 | A | G | -0.0287 | 0.0416 | 0.0043 | 0.016634427 | 2.48E-11 | 0.01239 | 44.54786371 |
| rs12344515 | T | C | -0.0163 | -0.0179 | 0.0022 | 0.008233916 | 1.27E-13 | 0.02971 | 54.8946281 |
| rs12351226 | T | C | 0.0218 | -0.0135 | 0.0025 | 0.009709265 | 2.78E-18 | 0.1644 | 76.0384 |
| rs12371664 | A | G | 0.0168 | 0.0247 | 0.0021 | 0.00805413 | 1.24E-15 | 0.002164 | 64 |
| rs12423821 | T | C | -0.0161 | 0.006 | 0.0027 | 0.016008009 | 2.48E-09 | 0.7078 | 35.5569273 |
| rs12463908 | A | C | 0.0165 | 0.0124 | 0.0024 | 0.009233655 | 6.20E-12 | 1.79E-01 | 47.265625 |
| rs12474969 | A | G | 0.0167 | 0.0014 | 0.002 | 0.008391532 | 6.83E-17 | 0.8675 | 69.7225 |
| rs12483401 | T | C | 0.0387 | -0.0156 | 0.0067 | 0.026012604 | 7.64E-09 | 0.5487 | 33.36355536 |
| rs12509014 | T | C | -0.0263 | -0.0094 | 0.0023 | 0.00857881 | 2.80E-30 | 0.2732 | 130.7542533 |
| rs12512942 | A | G | -0.0162 | 0.0039 | 0.002 | 0.007316407 | 5.50E-16 | 0.594 | 65.61 |
| rs12533452 | T | C | 0.0237 | 0.0149 | 0.0026 | 0.009438018 | 7.84E-20 | 0.1144 | 83.09023669 |
| rs12595051 | A | G | 0.0179 | -0.0249 | 0.0021 | 0.007814682 | 1.54E-17 | 0.001441 | 72.6553288 |
| rs12612857 | A | G | -0.0124 | -0.0103 | 0.0022 | 0.008126653 | 1.74E-08 | 0.205 | 31.76859504 |
| rs12622189 | A | G | 0.0272 | 0.0147 | 0.0021 | 0.007954476 | 2.28E-38 | 0.0646 | 167.7641723 |
| rs12655296 | T | C | -0.011 | -0.0039 | 0.002 | 0.007398487 | 3.80E-08 | 0.5981 | 30.25 |
| rs12662115 | A | G | 0.0152 | -0.007 | 0.002 | 0.00784765 | 2.96E-14 | 0.3724 | 57.76 |
| rs12663031 | T | C | -0.0195 | -0.0158 | 0.0019 | 0.008926337 | 1.03E-24 | 0.07672 | 105.33241 |
| rs12679359 | T | G | -0.0263 | -0.0209 | 0.0028 | 0.010318636 | 5.84E-21 | 0.04282 | 88.22576531 |
| rs12700901 | A | C | -0.0184 | -0.0052 | 0.0019 | 0.00715066 | 3.52E-22 | 0.4671 | 93.78393352 |
| rs12702693 | T | C | 0.0173 | -0.0063 | 0.0019 | 0.007045184 | 8.61E-20 | 0.3712 | 82.90581717 |
| rs12713004 | A | G | -0.0367 | -0.0001 | 0.0021 | 0.01108158 | 2.18E-68 | 0.9928 | 305.4172336 |
| rs12714414 | T | C | 0.0353 | 0.0523 | 0.0027 | 0.011634834 | 4.63E-39 | 6.95E-06 | 170.9314129 |
| rs12761076 | A | G | -0.026 | 0.0018 | 0.0021 | 0.008154632 | 3.31E-35 | 0.8253 | 153.2879819 |
| rs12894822 | A | G | -0.0135 | -0.0061 | 0.0022 | 0.008048363 | 8.44E-10 | 0.4485 | 37.65495868 |
| rs12907384 | T | C | 0.0269 | 0.0117 | 0.0019 | 0.007054639 | 1.67E-45 | 0.09722 | 200.4459834 |
| rs1290786 | T | C | -0.0143 | -0.0008 | 0.0019 | 0.007093062 | 5.22E-14 | 0.9102 | 56.64542936 |
| rs12997625 | T | C | -0.017 | 0.0049 | 0.0019 | 0.007052703 | 3.64E-19 | 0.4872 | 80.05540166 |
| rs13109280 | A | G | -0.0131 | -0.001 | 0.002 | 0.007512128 | 5.75E-11 | 0.8941 | 42.9025 |
| rs13112742 | A | G | -0.0151 | 0.0124 | 0.0025 | 0.009622048 | 1.54E-09 | 0.1975 | 36.4816 |
| rs13127468 | A | C | -0.0123 | 0.0023 | 0.0019 | 0.007461727 | 9.56E-11 | 0.7579 | 41.90858726 |
| rs13170063 | A | G | -0.0152 | 0.0189 | 0.0019 | 0.00978796 | 1.24E-15 | 0.05349 | 64 |
| rs1317349 | T | G | -0.0258 | -0.0047 | 0.0021 | 0.008051627 | 1.08E-34 | 0.5594 | 150.9387755 |
| rs13193017 | A | G | 0.0164 | -0.0114 | 0.0027 | 0.010378086 | 1.25E-09 | 0.272 | 36.89437586 |
| rs13209574 | T | G | -0.0292 | 0.0221 | 0.0032 | 0.011722302 | 7.17E-20 | 0.05939 | 83.265625 |
| rs13209685 | T | G | 0.0277 | 0.0137 | 0.0026 | 0.009563682 | 1.67E-26 | 0.152 | 113.5044379 |
| rs1325596 | A | G | 0.0287 | 0.0093 | 0.0019 | 0.007158648 | 1.50E-51 | 0.1939 | 228.1689751 |
| rs13316 | A | C | 0.0115 | -0.0001 | 0.0019 | 0.006878073 | 1.42E-09 | 0.9884 | 36.63434903 |
| rs13321258 | A | G | 0.0133 | -0.0012 | 0.0022 | 0.008497673 | 1.49E-09 | 0.8877 | 36.54752066 |
| rs1341215 | A | G | 0.0229 | 0.0063 | 0.0027 | 0.01014742 | 2.22E-17 | 0.5347 | 71.93552812 |
| rs1355603 | T | C | -0.0466 | -0.0148 | 0.0025 | 0.009462463 | 1.52E-77 | 0.1178 | 347.4496 |
| rs139921635 | T | G | 0.0385 | -0.0224 | 0.0062 | 0.025286535 | 5.31E-10 | 0.3757 | 38.56009365 |
| rs1405227 | A | G | 0.0129 | -0.0105 | 0.002 | 0.007641175 | 1.12E-10 | 0.1694 | 41.6025 |
| rs141277904 | T | C | 0.0384 | -0.0263 | 0.0069 | 0.028440357 | 2.62E-08 | 0.3551 | 30.97164461 |
| rs1430157 | T | C | 0.0182 | 0.0127 | 0.002 | 0.007642479 | 9.03E-20 | 0.09656 | 82.81 |
| rs143076454 | A | G | -0.0499 | 0.0108 | 0.007 | 0.026794649 | 1.01E-12 | 0.6869 | 50.81653061 |
| rs1436164 | T | C | -0.0139 | 0.0044 | 0.0019 | 0.007111441 | 2.56E-13 | 0.5361 | 53.52077562 |
| rs144109601 | A | C | -0.0278 | -0.0165 | 0.0048 | 0.017684154 | 6.97E-09 | 0.3508 | 33.54340278 |
| rs1444628 | T | C | 0.024 | 0.0038 | 0.002 | 0.007620012 | 3.55E-33 | 0.618 | 144 |
| rs1447691 | A | G | 0.0181 | 0.0117 | 0.002 | 0.007458041 | 1.43E-19 | 0.1167 | 81.9025 |
| rs145147649 | A | G | -0.0362 | -0.0002 | 0.0045 | 0.015799272 | 8.66E-16 | 9.90E-01 | 64.71308642 |
| rs147110934 | T | G | -0.0722 | -0.0253 | 0.0062 | 0.025273325 | 2.43E-31 | 0.3168 | 135.6097815 |
| rs1473441 | A | G | 0.0198 | 0.0108 | 0.0021 | 0.007850281 | 4.16E-21 | 0.1689 | 88.89795918 |
| rs1487441 | A | G | 0.0136 | 0.005 | 0.0019 | 0.007051794 | 8.19E-13 | 0.4783 | 51.23545706 |
| rs149697773 | A | G | 0.0262 | -0.0191 | 0.0047 | 0.019301937 | 2.48E-08 | 0.3224 | 31.07469443 |
| rs1557341 | A | C | -0.0153 | 0.0061 | 0.002 | 0.007538308 | 2.01E-14 | 0.4184 | 58.5225 |
| rs165849 | A | G | 0.0157 | 0.0203 | 0.0021 | 0.010393385 | 7.65E-14 | 0.0508 | 55.89342404 |
| rs1662842 | A | G | -0.0214 | -0.0224 | 0.002 | 0.007584997 | 1.02E-26 | 0.003145 | 114.49 |
| rs16844417 | A | G | 0.0226 | 0.0138 | 0.0029 | 0.010972403 | 6.54E-15 | 0.2085 | 60.73246136 |
| rs17036160 | T | C | -0.0375 | 0.0067 | 0.0029 | 0.010606739 | 3.01E-38 | 0.5276 | 167.2116528 |
| rs17197114 | T | C | -0.0177 | -0.0187 | 0.0025 | 0.009762913 | 1.44E-12 | 0.05544 | 50.1264 |
| rs17205463 | T | C | -0.0263 | -0.0063 | 0.0019 | 0.007080639 | 1.42E-43 | 0.3736 | 191.6038781 |
| rs17428810 | T | C | 0.0155 | 0.0001 | 0.002 | 0.007978637 | 9.19E-15 | 0.99 | 60.0625 |
| rs17478946 | A | G | 0.0192 | 0.0082 | 0.0021 | 0.007908475 | 6.08E-20 | 0.2998 | 83.59183673 |
| rs17496249 | A | G | -0.0123 | -0.0115 | 0.0019 | 0.007142901 | 9.56E-11 | 0.1074 | 41.90858726 |
| rs17681189 | A | C | -0.0131 | 0.0016 | 0.0019 | 0.007063514 | 5.40E-12 | 0.8208 | 47.53739612 |
| rs17713523 | A | G | 0.0114 | 0.0126 | 0.0019 | 0.00720576 | 1.97E-09 | 0.08036 | 36 |
| rs17773965 | T | C | -0.0163 | 0.0052 | 0.0027 | 0.010045328 | 1.57E-09 | 0.6047 | 36.44581619 |
| rs1805165 | A | C | -0.0184 | -0.0137 | 0.0021 | 0.00781694 | 1.92E-18 | 0.07967 | 76.77097506 |
| rs1809179 | T | C | -0.0155 | 0.0013 | 0.0026 | 0.009637587 | 2.50E-09 | 0.8927 | 35.53994083 |
| rs181766 | T | C | -0.0221 | -0.0003 | 0.002 | 0.007976965 | 2.19E-28 | 0.97 | 122.1025 |
| rs1899040 | T | C | 0.0152 | -0.0049 | 0.0023 | 0.008743603 | 3.88E-11 | 0.5752 | 43.67485822 |
| rs190801170 | A | G | 0.0286 | 0.0199 | 0.0036 | 0.017600405 | 1.95E-15 | 0.2582 | 63.11419753 |
| rs1977337 | T | C | -0.0186 | -0.008 | 0.0026 | 0.009991143 | 8.44E-13 | 0.4233 | 51.17751479 |
| rs200439 | A | C | 0.0128 | 0.0032 | 0.0023 | 0.008760326 | 2.62E-08 | 0.7149 | 30.97164461 |
| rs2007022 | A | C | 0.0179 | 0.0186 | 0.0022 | 0.008562717 | 4.07E-16 | 0.02984 | 66.20041322 |
| rs200739311 | T | C | 0.0128 | 0.0339 | 0.002 | 0.011080698 | 1.55E-10 | 0.002218 | 40.96 |
| rs200776140 | A | G | -0.015 | 0.0268 | 0.0027 | 0.014574474 | 2.77E-08 | 0.06594 | 30.86419753 |
| rs201570119 | T | C | -0.0194 | 0.0061 | 0.0023 | 0.012094248 | 3.32E-17 | 0.614 | 71.14555766 |
| rs201764844 | T | C | -0.0196 | -0.0051 | 0.0021 | 0.011549683 | 1.03E-20 | 0.6588 | 87.11111111 |
| rs2019203 | A | C | 0.0189 | -0.009 | 0.0019 | 0.007022737 | 2.59E-23 | 0.2 | 98.9501385 |
| rs2052478 | T | C | -0.0195 | -0.0122 | 0.0022 | 0.008510326 | 7.74E-19 | 0.1517 | 78.56404959 |
| rs2070598 | A | G | 0.0204 | 0.0085 | 0.0019 | 0.007066029 | 6.83E-27 | 0.229 | 115.2797784 |
| rs2071518 | T | C | -0.0236 | -0.0032 | 0.0021 | 0.008022964 | 2.65E-29 | 0.69 | 126.2947846 |
| rs2098695 | A | G | 0.0255 | 0.0003 | 0.002 | 0.00822379 | 3.12E-37 | 0.9709 | 162.5625 |
| rs2112617 | A | G | -0.0167 | 0.0047 | 0.0019 | 0.007033724 | 1.50E-18 | 0.504 | 77.25484765 |
| rs2125125 | T | C | -0.0158 | -0.0087 | 0.0023 | 0.008626415 | 6.44E-12 | 0.3132 | 47.19092628 |
| rs212526 | T | C | -0.0214 | -0.0113 | 0.0019 | 0.007218816 | 1.99E-29 | 0.1175 | 126.8587258 |
| rs2126942 | T | C | 0.0127 | 0.0013 | 0.0019 | 0.007386687 | 2.32E-11 | 0.8603 | 44.67867036 |
| rs2138374 | T | C | 0.0149 | 0.0004 | 0.002 | 0.006801064 | 9.33E-14 | 0.9531 | 55.5025 |
| rs2140046 | T | C | 0.0192 | -0.004 | 0.0019 | 0.007309138 | 5.24E-24 | 0.5842 | 102.1163435 |
| rs2142644 | A | C | -0.0181 | 0.0023 | 0.002 | 0.010073724 | 1.43E-19 | 0.8194 | 81.9025 |
| rs2165772 | A | G | 0.016 | -0.0068 | 0.002 | 0.007442663 | 1.24E-15 | 0.3609 | 64 |
| rs2208404 | A | G | 0.0111 | 0.0054 | 0.0019 | 0.00795772 | 5.15E-09 | 0.4974 | 34.13019391 |
| rs2209098 | T | C | -0.024 | -0.0125 | 0.002 | 0.00756536 | 3.55E-33 | 0.09848 | 144 |
| rs2212926 | A | C | -0.022 | -0.0088 | 0.0023 | 0.009173211 | 1.12E-21 | 0.3374 | 91.49338374 |
| rs2252031 | T | C | 0.0176 | 0.0032 | 0.0026 | 0.009834915 | 1.29E-11 | 0.7449 | 45.82248521 |
| rs2280463 | A | G | 0.0147 | 0.0128 | 0.0021 | 0.007825341 | 2.56E-12 | 0.1019 | 49 |
| rs2287821 | T | C | -0.0153 | -0.0003 | 0.0019 | 0.006996843 | 8.10E-16 | 0.9658 | 64.84487535 |
| rs2289976 | A | G | 0.0144 | -0.0002 | 0.002 | 0.008099528 | 6.02E-13 | 0.9803 | 51.84 |
| rs2296316 | T | C | 0.0192 | -0.0106 | 0.0019 | 0.007129993 | 5.24E-24 | 0.1371 | 102.1163435 |
| rs2298333 | T | C | -0.0267 | -0.0009 | 0.0019 | 0.007369497 | 7.42E-45 | 0.9028 | 197.4764543 |
| rs2303423 | T | C | -0.0168 | -0.0061 | 0.003 | 0.01116425 | 2.14E-08 | 0.5848 | 31.36 |
| rs2304655 | T | C | -0.0113 | -0.0066 | 0.0019 | 0.007169844 | 2.72E-09 | 0.3573 | 35.37119114 |
| rs2305141 | A | G | -0.0183 | -0.0035 | 0.0019 | 0.007206515 | 5.88E-22 | 0.6272 | 92.76731302 |
| rs2324154 | A | C | 0.015 | 0.0064 | 0.0019 | 0.007006318 | 2.91E-15 | 0.361 | 62.32686981 |
| rs2347808 | A | G | -0.0125 | -0.0055 | 0.0019 | 0.007089965 | 4.74E-11 | 0.4379 | 43.28254848 |
| rs2348496 | A | G | 0.0136 | 0.0012 | 0.0019 | 0.010858136 | 8.19E-13 | 0.912 | 51.23545706 |
| rs2436772 | A | G | 0.0222 | -0.0093 | 0.0023 | 0.008794628 | 4.81E-22 | 0.2903 | 93.16446125 |
| rs2487 | T | C | -0.014 | -0.0062 | 0.0019 | 0.007189733 | 1.73E-13 | 0.3885 | 54.29362881 |
| rs2506697 | A | G | 0.0141 | 0.0066 | 0.002 | 0.007878729 | 1.79E-12 | 0.4022 | 49.7025 |
| rs2531991 | A | G | 0.0189 | 8.90E-03 | 0.0022 | 0.008158114 | 8.63E-18 | 0.2753 | 73.80371901 |
| rs2539251 | T | G | -0.0162 | -0.0461 | 0.0027 | 0.010571505 | 1.97E-09 | 0.000013 | 36 |
| rs2549677 | A | G | 0.0392 | 0.0084 | 0.0032 | 0.015996354 | 1.68E-34 | 0.5995 | 150.0625 |
| rs2569888 | A | G | 0.0133 | -0.018 | 0.0022 | 0.008418756 | 1.49E-09 | 0.03251 | 36.54752066 |
| rs2578565 | T | C | -0.0141 | -0.0094 | 0.002 | 0.007383837 | 1.79E-12 | 0.203 | 49.7025 |
| rs2596144 | T | C | 0.0223 | -0.004 | 0.0028 | 0.010073246 | 1.66E-15 | 0.6913 | 63.42984694 |
| rs2607234 | A | G | 0.0302 | -0.0178 | 0.0043 | 0.015736463 | 2.17E-12 | 0.258 | 49.32612223 |
| rs2609334 | T | C | 0.0171 | -0.0041 | 0.0022 | 0.008366529 | 7.68E-15 | 0.6241 | 60.41528926 |
| rs261999 | T | C | 0.0175 | -0.0062 | 0.0019 | 0.007223172 | 3.25E-20 | 0.3907 | 84.83379501 |
| rs2627702 | T | C | 0.0191 | 0.008 | 0.0019 | 0.007069597 | 8.94E-24 | 0.2578 | 101.0554017 |
| rs2651472 | T | G | 0.0108 | 0.0017 | 0.0019 | 0.007139721 | 1.31E-08 | 0.8118 | 32.31024931 |
| rs2663126 | A | G | -0.0139 | 0.005 | 0.0021 | 0.007811137 | 3.62E-11 | 0.5221 | 43.81179138 |
| rs2678898 | T | C | 0.0129 | -0.0047 | 0.0019 | 0.009702668 | 1.13E-11 | 0.6281 | 46.09695291 |
| rs2721940 | A | C | 0.0165 | 0.0147 | 0.0019 | 0.007250613 | 3.81E-18 | 0.04262 | 75.41551247 |
| rs2788213 | A | G | 0.0123 | 0.0056 | 0.0021 | 0.00768345 | 4.71E-09 | 4.66E-01 | 34.30612245 |
| rs2807339 | T | C | -0.0162 | 0.0049 | 0.0022 | 0.008195214 | 1.79E-13 | 0.5499 | 54.2231405 |
| rs2823990 | A | G | -0.0127 | -0.0079 | 0.002 | 0.007859123 | 2.15E-10 | 0.3148 | 40.3225 |
| rs28468602 | T | G | -0.0112 | 0.0087 | 0.0019 | 0.007405914 | 3.75E-09 | 0.2401 | 34.74792244 |
| rs28529055 | T | G | -0.0147 | 0.0042 | 0.0019 | 0.009656321 | 1.02E-14 | 0.6636 | 59.85872576 |
| rs2854152 | A | G | -0.0482 | -0.0015 | 0.002 | 0.007815503 | 2.50E-128 | 0.8478 | 580.81 |
| rs28701981 | T | C | -0.0395 | 0.0082 | 0.002 | 0.007477404 | 8.02E-87 | 0.2728 | 390.0625 |
| rs28736838 | T | C | -0.0117 | -0.0077 | 0.002 | 0.007598669 | 4.92E-09 | 0.3109 | 34.2225 |
| rs28757154 | A | G | -0.0193 | 0.0143 | 0.0027 | 0.01253366 | 8.80E-13 | 0.2539 | 51.09602195 |
| rs28787734 | T | C | 0.0385 | -0.0104 | 0.0041 | 0.023039342 | 5.99E-21 | 0.6517 | 88.17668055 |
| rs28817902 | A | G | -0.023 | 0.0082 | 0.0029 | 0.01166015 | 2.17E-15 | 4.82E-01 | 62.90130797 |
| rs2900208 | A | C | 0.0255 | 0.0176 | 0.002 | 0.007390148 | 3.12E-37 | 0.01724 | 162.5625 |
| rs291970 | T | C | 0.024 | 0.0187 | 0.0022 | 0.008807604 | 1.04E-27 | 0.03374 | 119.0082645 |
| rs2925155 | T | C | -0.015 | -0.0195 | 0.0022 | 0.008154648 | 9.22E-12 | 0.01679 | 46.48760331 |
| rs2965074 | T | G | 0.0125 | 0.0022 | 0.0019 | 0.007186315 | 4.74E-11 | 0.7595 | 43.28254848 |
| rs2974337 | T | C | 0.0117 | 0.0032 | 0.0019 | 0.007065128 | 7.37E-10 | 0.6506 | 37.91966759 |
| rs2993531 | A | C | -0.0176 | -0.0126 | 0.0019 | 0.007144493 | 1.98E-20 | 0.0778 | 85.80609418 |
| rs2994329 | T | C | 0.0155 | -0.0057 | 0.0023 | 0.008618981 | 1.59E-11 | 0.5084 | 45.41587902 |
| rs3003137 | T | G | -0.0114 | 0.008 | 0.0019 | 0.007725169 | 1.97E-09 | 0.3004 | 36 |
| rs301805 | T | G | 0.0146 | 0.0172 | 0.0019 | 0.007138113 | 1.54E-14 | 0.01597 | 59.04709141 |
| rs3103223 | T | C | -0.0126 | -0.0059 | 0.0022 | 0.008109607 | 1.02E-08 | 0.4669 | 32.80165289 |
| rs3103268 | T | C | 0.0291 | 0.0369 | 0.0031 | 0.012185382 | 6.17E-21 | 2.46E-03 | 88.11758585 |
| rs3115084 | T | C | 0.024 | 0.023 | 0.0031 | 0.015214256 | 9.79E-15 | 0.1306 | 59.93756504 |
| rs3116602 | T | G | 0.0612 | 0.0324 | 0.0023 | 0.008628358 | 5.38E-156 | 0.0001733 | 708.0226843 |
| rs31196 | A | C | -0.0107 | 0.0046 | 0.0019 | 0.007050166 | 1.79E-08 | 0.5141 | 31.71468144 |
| rs332116 | T | C | -0.0206 | 0.006 | 0.0021 | 0.009771618 | 1.02E-22 | 0.5392 | 96.22675737 |
| rs33973388 | T | G | 0.0249 | -0.0378 | 0.0019 | 0.00749666 | 3.07E-39 | 0.00000046 | 171.7479224 |
| rs34287 | A | G | 0.0187 | -0.0019 | 0.002 | 0.007534226 | 8.76E-21 | 0.8009 | 87.4225 |
| rs34345560 | A | G | 0.0219 | 0.0031 | 0.0024 | 0.008951182 | 7.17E-20 | 0.7291 | 83.265625 |
| rs34522021 | T | C | 0.0126 | -0.0073 | 0.0019 | 0.007154962 | 3.32E-11 | 0.3076 | 43.97783934 |
| rs34786000 | T | G | -0.015 | -0.0034 | 0.0019 | 0.007355282 | 2.91E-15 | 6.44E-01 | 62.32686981 |
| rs35073631 | T | C | -0.0112 | -0.0096 | 0.0019 | 0.00960376 | 3.75E-09 | 0.3175 | 34.74792244 |
| rs35268848 | A | C | 0.0737 | 0.0277 | 0.0101 | 0.038996002 | 2.94E-13 | 0.4775 | 53.24664249 |
| rs35453327 | A | C | 0.0283 | 0.0015 | 0.0036 | 0.013761071 | 3.81E-15 | 0.9132 | 61.7970679 |
| rs35464459 | T | C | 0.0342 | -0.0122 | 0.0029 | 0.010903959 | 4.24E-32 | 0.2632 | 139.0772889 |
| rs35624335 | T | C | -0.0134 | -0.0062 | 0.0021 | 0.007847629 | 1.76E-10 | 0.4295 | 40.71655329 |
| rs35696197 | T | G | 0.0127 | 0.0123 | 0.0021 | 0.008218684 | 1.47E-09 | 0.1345 | 36.57369615 |
| rs35732917 | T | C | -0.0204 | -0.0028 | 0.0021 | 0.007974532 | 2.62E-22 | 0.7255 | 94.36734694 |
| rs35748083 | T | C | -0.0197 | 0.0091 | 0.0019 | 0.007168766 | 3.45E-25 | 0.2043 | 107.5041551 |
| rs35756741 | T | C | -0.0378 | -0.0064 | 0.0033 | 0.012271666 | 2.23E-30 | 0.602 | 131.2066116 |
| rs35816944 | A | G | -0.1088 | -0.0718 | 0.0117 | 0.048676426 | 1.42E-20 | 0.1402 | 86.47410329 |
| rs35874463 | A | G | -0.0263 | -0.0485 | 0.0041 | 0.017709041 | 1.41E-10 | 0.006168 | 41.14753123 |
| rs35892992 | T | C | -0.0182 | 0.0044 | 0.0023 | 0.008704064 | 2.51E-15 | 0.6132 | 62.61625709 |
| rs35963161 | A | G | -0.0157 | 0.0178 | 0.0019 | 0.008380879 | 1.42E-16 | 0.03368 | 68.27977839 |
| rs36048468 | T | C | 0.0254 | -0.0001 | 0.0023 | 0.015343825 | 2.36E-28 | 9.95E-01 | 121.9584121 |
| rs36226649 | T | C | -0.0485 | -0.0233 | 0.0038 | 0.014393778 | 2.63E-37 | 0.1055 | 162.8981994 |
| rs3625 | A | G | 0.0148 | -0.0106 | 0.0019 | 0.007035469 | 6.73E-15 | 0.1319 | 60.67590028 |
| rs372532055 | A | C | 0.0175 | 0.0334 | 0.002 | 0.011894033 | 2.13E-18 | 0.004983 | 76.5625 |
| rs3769885 | A | G | -0.0111 | -0.0119 | 0.0019 | 0.007028169 | 5.15E-09 | 0.09042 | 34.13019391 |
| rs377599 | T | C | 0.0217 | 0.0058 | 0.0019 | 0.00727806 | 3.28E-30 | 0.4255 | 130.4404432 |
| rs3792819 | A | G | -0.021 | -0.0237 | 0.0034 | 0.012684341 | 6.56E-10 | 0.0617 | 38.14878893 |
| rs3814333 | T | C | 0.0182 | -0.0209 | 0.002 | 0.007586481 | 9.03E-20 | 0.005871 | 82.81 |
| rs3830008 | A | C | 0.0154 | 0.0013 | 0.0023 | 0.008856359 | 2.15E-11 | 0.8833 | 44.83175803 |
| rs3844 | A | C | 0.0132 | -0.0036 | 0.0022 | 0.008150167 | 1.97E-09 | 0.6587 | 36 |
| rs3853252 | A | G | 0.0241 | -0.0121 | 0.0019 | 0.007108049 | 7.23E-37 | 0.0887 | 160.8891967 |
| rs395980 | T | G | 0.0184 | 0.0224 | 0.0021 | 0.007959098 | 1.92E-18 | 0.004887 | 76.77097506 |
| rs4121583 | T | C | 0.0118 | -0.0046 | 0.002 | 0.008832417 | 3.64E-09 | 0.6025 | 34.81 |
| rs41298373 | A | G | 0.0185 | 0.0117 | 0.0031 | 0.012216414 | 2.41E-09 | 0.3382 | 35.61394381 |
| rs4252548 | T | C | -0.0753 | 0.009 | 0.0065 | 0.022719105 | 4.93E-31 | 6.92E-01 | 134.2033136 |
| rs4274112 | A | G | 0.0217 | -0.0071 | 0.002 | 0.007393458 | 1.99E-27 | 0.3369 | 117.7225 |
| rs4287835 | T | C | -0.0147 | 0.0071 | 0.0019 | 0.007079336 | 1.02E-14 | 0.3159 | 59.85872576 |
| rs4383083 | A | G | 0.0111 | -0.0137 | 0.002 | 0.007827319 | 2.86E-08 | 0.08007 | 30.8025 |
| rs4444637 | A | G | -0.0179 | 0.0226 | 0.0027 | 0.010141543 | 3.37E-11 | 0.02585 | 43.95198903 |
| rs447352 | T | C | -0.0181 | 0.0227 | 0.0029 | 0.016012687 | 4.34E-10 | 0.1563 | 38.9548157 |
| rs4504126 | A | C | -0.046 | 0.0291 | 0.0058 | 0.022409691 | 2.17E-15 | 0.1941 | 62.90130797 |
| rs45474992 | T | C | -0.0617 | 0.0118 | 0.0051 | 0.019755215 | 1.08E-33 | 0.5503 | 146.3625529 |
| rs4554207 | T | C | 0.0114 | 0.0211 | 0.0019 | 0.007361014 | 1.97E-09 | 0.004151 | 36 |
| rs4615815 | A | G | 0.0246 | -0.0066 | 0.0019 | 0.007249385 | 2.43E-38 | 0.3626 | 167.634349 |
| rs4622329 | A | G | 0.0149 | 0.0048 | 0.002 | 0.007384752 | 9.33E-14 | 0.5157 | 55.5025 |
| rs4652902 | A | C | 0.0129 | 0.0311 | 0.0022 | 0.008320892 | 4.53E-09 | 0.0001858 | 34.3822314 |
| rs465983 | A | G | 0.0153 | 0.0124 | 0.0022 | 0.008193939 | 3.54E-12 | 0.1302 | 48.36570248 |
| rs4682483 | A | G | -0.0165 | -0.0162 | 0.0026 | 0.009509399 | 2.21E-10 | 0.08846 | 40.27366864 |
| rs4733775 | A | G | 0.0143 | 0.0203 | 0.002 | 0.007321177 | 8.68E-13 | 0.005558 | 51.1225 |
| rs4752829 | A | G | 0.0262 | 0.0293 | 0.0021 | 0.008146576 | 1.01E-35 | 0.0003224 | 155.6553288 |
| rs4754296 | T | G | 0.0167 | 0.0175 | 0.0027 | 0.010170433 | 6.20E-10 | 0.08531 | 38.25651578 |
| rs4763327 | T | C | 0.0123 | -0.0007 | 0.0021 | 0.007386547 | 4.71E-09 | 0.9245 | 34.30612245 |
| rs4776624 | T | C | -0.015 | 0.0028 | 0.0019 | 0.007138957 | 2.91E-15 | 0.6949 | 62.32686981 |
| rs4799799 | A | C | -0.0113 | -0.0107 | 0.002 | 0.008030093 | 1.60E-08 | 0.1827 | 31.9225 |
| rs4815952 | T | C | 0.0161 | 0.0056 | 0.0019 | 0.007462458 | 2.38E-17 | 0.453 | 71.8033241 |
| rs4847378 | T | G | 0.0136 | 0.0019 | 0.0019 | 0.007204764 | 8.19E-13 | 0.792 | 51.23545706 |
| rs4852257 | T | G | 0.0231 | 0.0104 | 0.0019 | 0.007233436 | 5.21E-34 | 0.1505 | 147.8144044 |
| rs4909912 | T | C | -0.0277 | -0.0047 | 0.0019 | 0.007045284 | 3.82E-48 | 0.5047 | 212.5457064 |
| rs4940874 | A | G | -0.0148 | 0.0049 | 0.0024 | 0.009093675 | 6.97E-10 | 0.59 | 38.02777778 |
| rs496783 | A | G | 0.0124 | 0.0126 | 0.0019 | 0.007472256 | 6.74E-11 | 0.09175 | 42.59279778 |
| rs4976262 | T | C | 0.0245 | -0.0223 | 0.002 | 0.007523184 | 1.68E-34 | 0.003035 | 150.0625 |
| rs4979576 | T | C | 0.0317 | -0.0001 | 0.0029 | 0.010929773 | 8.19E-28 | 0.9927 | 119.4875149 |
| rs4985445 | A | G | 0.0175 | -0.0381 | 0.0019 | 0.007106731 | 3.25E-20 | 8.27E-08 | 84.83379501 |
| rs501811 | T | C | 0.0199 | -0.0069 | 0.003 | 0.010740624 | 3.28E-11 | 5.21E-01 | 44.00111111 |
| rs548466113 | T | C | -0.036 | 0.0028 | 0.0021 | 0.011778673 | 7.11E-66 | 0.8121 | 293.877551 |
| rs551473284 | T | C | 0.017 | 0.0059 | 0.002 | 0.010914288 | 1.90E-17 | 0.5888 | 72.25 |
| rs55633823 | T | C | 0.0146 | -0.0223 | 0.0022 | 0.00911552 | 3.22E-11 | 0.01443 | 44.04132231 |
| rs55745410 | A | G | 0.0157 | -0.0086 | 0.002 | 0.007458639 | 4.16E-15 | 0.2489 | 61.6225 |
| rs55758152 | A | G | 0.0145 | 0.004 | 0.002 | 0.009024677 | 4.17E-13 | 0.6576 | 52.5625 |
| rs56034226 | T | C | 0.0226 | 0.0197 | 0.0027 | 0.012516315 | 5.74E-17 | 0.1155 | 70.06310014 |
| rs56207248 | T | C | -0.0273 | -0.0003 | 0.0038 | 0.016282437 | 6.76E-13 | 0.9853 | 51.61288089 |
| rs56207600 | A | G | 0.0192 | -0.0066 | 0.003 | 0.011449064 | 1.55E-10 | 0.5643 | 40.96 |
| rs56208656 | A | G | -0.0284 | -0.008 | 0.0034 | 0.013022404 | 6.66E-17 | 0.539 | 69.7716263 |
| rs56309431 | T | G | 0.0167 | -0.0039 | 0.0029 | 0.010831437 | 8.48E-09 | 0.7188 | 33.16171225 |
| rs56363908 | A | G | 0.0382 | -0.0284 | 0.0047 | 0.018251529 | 4.38E-16 | 0.1197 | 66.05885016 |
| rs563738408 | A | G | -0.0587 | -0.0942 | 0.0053 | 0.030400268 | 1.65E-28 | 0.001944 | 122.6660733 |
| rs57287582 | T | G | 0.0159 | 0 | 0.0025 | NA | 2.02E-10 | 0.9979 | 40.4496 |
| rs57307236 | A | G | -0.0155 | -0.0485 | 0.0019 | 0.010678888 | 3.41E-16 | 0.00000558 | 66.55124654 |
| rs5742915 | T | C | -0.0248 | 0.0106 | 0.0019 | 0.00710094 | 6.14E-39 | 0.1355 | 170.3711911 |
| rs5763821 | A | C | -0.0191 | -0.0295 | 0.002 | 0.009737347 | 1.30E-21 | 0.002449 | 91.2025 |
| rs57904377 | T | C | 0.0177 | -0.0146 | 0.0024 | 0.009715433 | 1.64E-13 | 0.1329 | 54.390625 |
| rs58738817 | A | G | -0.0257 | 7.00E-04 | 0.0022 | 0.008363836 | 1.58E-31 | 0.9333 | 136.464876 |
| rs59000092 | A | G | 0.0196 | -0.0096 | 0.0021 | 0.007977028 | 1.03E-20 | 0.2288 | 87.11111111 |
| rs591668 | A | G | -0.0174 | 0.0059 | 0.0019 | 0.0072677 | 5.29E-20 | 0.4169 | 83.86703601 |
| rs59725651 | A | G | 0.0167 | -0.0034 | 0.002 | 0.008415481 | 6.83E-17 | 0.6862 | 69.7225 |
| rs59753424 | A | G | 0.02 | -0.0084 | 0.0022 | 0.01104158 | 9.82E-20 | 0.4468 | 82.6446281 |
| rs59950280 | A | G | -0.0254 | -0.0105 | 0.002 | 0.007861994 | 5.91E-37 | 0.1817 | 161.29 |
| rs59951000 | T | C | -0.04 | -0.035 | 0.0047 | 0.017214887 | 1.73E-17 | 0.04204 | 72.43096424 |
| rs6000890 | T | C | 0.0135 | -0.016 | 0.002 | 0.007288664 | 1.48E-11 | 0.02815 | 45.5625 |
| rs6028716 | A | G | -0.021 | -0.0137 | 0.0022 | 0.008123344 | 1.36E-21 | 0.0917 | 91.11570248 |
| rs60328144 | A | C | 0.0144 | 0.0044 | 0.0019 | 0.00739973 | 3.48E-14 | 0.5521 | 57.44044321 |
| rs603486 | T | C | -0.0127 | -0.0069 | 0.002 | 0.007309782 | 2.15E-10 | 0.3452 | 40.3225 |
| rs60408354 | A | G | 0.0259 | 0.0135 | 0.0036 | 0.01398834 | 6.27E-13 | 0.3345 | 51.76003086 |
| rs6054390 | A | G | -0.0188 | -0.0172 | 0.002 | 0.007392967 | 5.46E-21 | 0.01999 | 88.36 |
| rs60804050 | A | G | -0.0217 | 0.0145 | 0.0021 | 0.00808523 | 4.98E-25 | 0.07291 | 106.7777778 |
| rs6082354 | A | C | 0.024 | 0.0013 | 0.002 | 0.009503901 | 3.55E-33 | 0.8912 | 144 |
| rs612577 | T | C | 0.0152 | -0.0116 | 0.0026 | 0.009931364 | 5.03E-09 | 0.2428 | 34.17751479 |
| rs61729527 | T | C | -0.0346 | 0.0266 | 0.0043 | 0.016014764 | 8.52E-16 | 0.09672 | 64.74634938 |
| rs61732778 | A | G | 0.023 | -0.0056 | 0.0037 | 0.013179164 | 5.09E-10 | 0.6709 | 38.64134405 |
| rs61838776 | A | C | 0.0311 | -0.0215 | 0.0025 | 0.009856432 | 1.59E-35 | 0.02916 | 154.7536 |
| rs61940146 | A | G | -0.0112 | -0.0042 | 0.002 | 0.007418686 | 2.14E-08 | 0.5713 | 31.36 |
| rs61944841 | A | G | 0.0253 | 0.0372 | 0.002 | 0.00920188 | 1.12E-36 | 0.0000529 | 160.0225 |
| rs62033029 | A | G | -0.0141 | 0.01 | 0.0023 | 0.008835139 | 8.76E-10 | 0.2577 | 37.58223062 |
| rs62048221 | T | C | -0.0242 | -0.0089 | 0.0022 | 0.008391969 | 3.82E-28 | 0.2889 | 121 |
| rs62103240 | A | G | 0.0212 | -0.0174 | 0.0037 | 0.017139108 | 1.01E-08 | 0.31 | 32.82980278 |
| rs62143873 | A | G | -0.0115 | 0.0014 | 0.0019 | 0.007478438 | 1.42E-09 | 0.8515 | 36.63434903 |
| rs62177315 | T | C | 0.0177 | -0.0176 | 0.0032 | 0.01208513 | 3.18E-08 | 0.1453 | 30.59472656 |
| rs62305043 | T | C | -0.0269 | 0.0097 | 0.004 | 0.022287385 | 1.76E-11 | 0.6634 | 45.225625 |
| rs62372061 | T | C | 0.0394 | 0.02 | 0.0032 | 0.013411709 | 7.76E-35 | 0.1359 | 151.5976563 |
| rs62449290 | A | G | 0.0213 | -0.0171 | 0.0026 | 0.013614172 | 2.56E-16 | 0.2091 | 67.11390533 |
| rs62466110 | T | C | 0.0371 | 0.0097 | 0.0041 | 0.017259064 | 1.45E-19 | 0.5741 | 81.88042832 |
| rs62501195 | A | C | 0.0198 | 0.0158 | 0.0025 | 0.009576242 | 2.38E-15 | 0.09896 | 62.7264 |
| rs631312 | A | G | -0.0126 | -0.0034 | 0.0021 | 0.007794796 | 1.97E-09 | 0.6627 | 36 |
| rs6450136 | A | C | -0.0201 | 0.003 | 0.002 | 0.007586013 | 9.19E-24 | 0.6925 | 101.0025 |
| rs6450961 | T | C | 0.0118 | 0.0024 | 0.002 | 0.007538265 | 3.64E-09 | 0.7502 | 34.81 |
| rs6461948 | T | C | 0.0112 | 0.0059 | 0.002 | 0.007610582 | 2.14E-08 | 0.4382 | 31.36 |
| rs6469845 | T | C | 0.0135 | 0.0036 | 0.0022 | 0.008334889 | 8.44E-10 | 0.6658 | 37.65495868 |
| rs6501381 | T | C | 0.0341 | 0.0004 | 0.003 | 0.010707369 | 6.13E-30 | 0.9702 | 129.2011111 |
| rs6505216 | T | G | -0.0498 | -0.0607 | 0.0023 | 0.011590085 | 5.80E-104 | 0.000000163 | 468.8166352 |
| rs6543146 | T | G | -0.0154 | -0.004 | 0.0019 | 0.007078287 | 5.26E-16 | 0.572 | 65.69529086 |
| rs6544743 | T | G | 0.0217 | -0.0198 | 0.0024 | 0.008836189 | 1.54E-19 | 0.02504 | 81.75173611 |
| rs6567160 | T | C | -0.0488 | -0.0223 | 0.0022 | 0.008249402 | 5.15E-109 | 0.006867 | 492.0330579 |
| rs66613683 | T | C | -0.015 | -0.0108 | 0.0025 | 0.009430144 | 1.97E-09 | 0.2521 | 36 |
| rs6693481 | T | C | 0.0143 | 0.0034 | 0.002 | 0.007548333 | 8.68E-13 | 0.6524 | 51.1225 |
| rs670129 | T | C | -0.0118 | 0.0046 | 0.0019 | 0.007486038 | 5.28E-10 | 0.5389 | 38.57063712 |
| rs670318 | T | C | -0.0413 | -0.004 | 0.0044 | 0.017057866 | 6.21E-21 | 0.8146 | 88.10382231 |
| rs6738207 | A | G | 0.0127 | 0.0009 | 0.0019 | 0.006832297 | 2.32E-11 | 0.8952 | 44.67867036 |
| rs6739394 | T | C | -0.0144 | 0.0026 | 0.0019 | 0.007172985 | 3.48E-14 | 0.717 | 57.44044321 |
| rs6789000 | T | G | 0.0121 | 0.0178 | 0.002 | 0.011030787 | 1.45E-09 | 0.1066 | 36.6025 |
| rs68083605 | A | G | 0.0189 | 0.0048 | 0.0019 | 0.007268371 | 2.59E-23 | 0.509 | 98.9501385 |
| rs680882 | T | G | -0.0133 | 0.0023 | 0.0022 | 0.008146853 | 1.49E-09 | 0.7777 | 36.54752066 |
| rs6844176 | T | C | -0.0129 | -0.0032 | 0.0019 | 0.007530951 | 1.13E-11 | 0.6709 | 46.09695291 |
| rs6852065 | T | C | 0.0131 | 0.0092 | 0.0019 | 0.007552975 | 5.40E-12 | 0.2232 | 47.53739612 |
| rs6854705 | T | C | 0.0173 | 0.009 | 0.0024 | 0.009197237 | 5.66E-13 | 0.3278 | 51.96006944 |
| rs6899155 | T | C | 0.0282 | -0.0087 | 0.0019 | 0.007079359 | 7.83E-50 | 0.2191 | 220.2880886 |
| rs6910414 | A | G | 0.0142 | 0.0082 | 0.0024 | 0.00924699 | 3.29E-09 | 0.3752 | 35.00694444 |
| rs6923230 | T | C | 0.0127 | 0.006 | 0.0019 | 0.007028657 | 2.32E-11 | 0.3933 | 44.67867036 |
| rs6931421 | T | G | 0.0279 | -0.0128 | 0.002 | 0.00750274 | 3.15E-44 | 0.088 | 194.6025 |
| rs6943386 | T | C | -0.0106 | -0.0047 | 0.0019 | 0.007244702 | 2.42E-08 | 0.5165 | 31.12465374 |
| rs6960741 | T | C | -0.0185 | -0.0032 | 0.0029 | 0.011366255 | 1.78E-10 | 0.7783 | 40.69560048 |
| rs6962887 | T | G | 0.0127 | -0.0066 | 0.0021 | 0.008034745 | 1.47E-09 | 0.4114 | 36.57369615 |
| rs6963134 | A | G | -0.0137 | -0.0128 | 0.002 | 0.00743156 | 7.38E-12 | 0.085 | 46.9225 |
| rs6975015 | A | G | 0.0211 | 0.0018 | 0.0029 | 0.011119317 | 3.44E-13 | 0.8714 | 52.93816885 |
| rs6977416 | A | G | 0.0457 | -0.0373 | 0.002 | 0.007438956 | 1.46E-115 | 0.000000533 | 522.1225 |
| rs700677 | A | C | 0.0173 | 0.0193 | 0.002 | 0.007727969 | 5.15E-18 | 0.01251 | 74.8225 |
| rs7014590 | T | C | 0.0228 | -0.0061 | 0.0022 | 0.00802182 | 3.63E-25 | 0.447 | 107.4049587 |
| rs704832 | A | C | -0.012 | -0.0078 | 0.0022 | 0.008222233 | 4.91E-08 | 0.3428 | 29.75206612 |
| rs705953 | A | G | 0.019 | 0.0088 | 0.002 | 0.007454446 | 2.10E-21 | 0.2378 | 90.25 |
| rs7078507 | A | G | 0.02 | 0.013 | 0.0019 | 0.00726676 | 6.53E-26 | 0.07362 | 110.8033241 |
| rs7095087 | A | G | 0.0117 | -0.0051 | 0.002 | 0.007379494 | 4.92E-09 | 0.4895 | 34.2225 |
| rs7095472 | A | G | -0.0267 | -0.0159 | 0.0019 | 0.007067708 | 7.42E-45 | 0.02447 | 197.4764543 |
| rs7107356 | A | G | -0.0133 | -0.0019 | 0.0019 | 0.007204764 | 2.56E-12 | 0.792 | 49 |
| rs71384617 | T | C | 0.0133 | 0.0198 | 0.0021 | 0.011147776 | 2.40E-10 | 0.07571 | 40.11111111 |
| rs71519447 | A | G | -0.0716 | -0.0408 | 0.003 | 0.011326838 | 6.80E-126 | 0.0003157 | 569.6177778 |
| rs7164187 | A | G | 0.0151 | 0.0058 | 0.0019 | 0.007203113 | 1.91E-15 | 0.4207 | 63.16066482 |
| rs7171129 | T | C | 0.012 | 0.0087 | 0.002 | 0.007667208 | 1.97E-09 | 0.2565 | 36 |
| rs7185244 | T | C | 0.0148 | -0.0037 | 0.0023 | 0.008517549 | 1.24E-10 | 0.664 | 41.40642722 |
| rs718603 | T | C | 0.0131 | -0.0015 | 0.0021 | 0.007657545 | 4.43E-10 | 0.8447 | 38.9138322 |
| rs7225068 | A | G | -0.0141 | -0.007 | 0.0019 | 0.00753359 | 1.16E-13 | 0.3528 | 55.07202216 |
| rs7228151 | T | C | 0.0185 | -0.0161 | 0.0023 | 0.008553858 | 8.73E-16 | 0.05981 | 64.69754253 |
| rs7229520 | A | G | -0.0224 | -0.0028 | 0.002 | 0.00749999 | 4.08E-29 | 0.7089 | 125.44 |
| rs723149 | A | G | 0.0276 | 0.0092 | 0.0019 | 0.007465114 | 8.25E-48 | 0.2178 | 211.0138504 |
| rs7259285 | A | G | -0.0132 | 0.0126 | 0.0019 | 0.00827187 | 3.72E-12 | 0.1277 | 48.26592798 |
| rs72801818 | T | C | 0.0313 | -0.0022 | 0.0021 | 0.007724812 | 3.07E-50 | 0.7758 | 222.1519274 |
| rs72809820 | T | C | -0.0111 | 0.0135 | 0.002 | 0.007506577 | 2.86E-08 | 0.07211 | 30.8025 |
| rs72829852 | T | C | 0.0309 | -0.0021 | 0.0039 | 0.014281773 | 2.32E-15 | 0.8831 | 62.77514793 |
| rs72841270 | T | G | -0.0294 | -0.0084 | 0.0028 | 0.010351677 | 8.64E-26 | 0.4171 | 110.25 |
| rs7286917 | A | G | -0.0171 | 0.0083 | 0.0023 | 0.010471617 | 1.05E-13 | 0.428 | 55.27599244 |
| rs72908840 | T | G | 0.037 | 0.0152 | 0.0043 | 0.016186401 | 7.65E-18 | 0.3477 | 74.04002163 |
| rs7301341 | T | C | 0.0255 | -0.0181 | 0.002 | 0.007784197 | 3.12E-37 | 0.02006 | 162.5625 |
| rs73040028 | T | C | 0.0168 | -0.0197 | 0.0022 | 0.00837849 | 2.23E-14 | 0.01871 | 58.31404959 |
| rs73125634 | T | G | -0.0195 | -0.0114 | 0.0021 | 0.008370085 | 1.61E-20 | 0.1732 | 86.2244898 |
| rs73158215 | A | G | 0.0163 | -0.0078 | 0.0023 | 0.008664491 | 1.37E-12 | 0.368 | 50.22495274 |
| rs73186333 | A | C | -0.0373 | -0.0253 | 0.0063 | 0.022456402 | 3.21E-09 | 0.2599 | 35.05391786 |
| rs7320878 | A | G | -0.015 | 0.0008 | 0.0019 | 0.008695885 | 2.91E-15 | 0.9267 | 62.32686981 |
| rs7328187 | T | G | -0.0116 | -0.0105 | 0.0019 | 0.007102259 | 1.03E-09 | 0.1393 | 37.27423823 |
| rs73382475 | T | C | 0.0254 | -0.0418 | 0.0033 | 0.012651491 | 1.39E-14 | 0.0009533 | 59.24334252 |
| rs73384223 | T | C | 0.0205 | 0.0132 | 0.0024 | 0.009075109 | 1.32E-17 | 0.1458 | 72.96006944 |
| rs73490624 | T | C | -0.0184 | -0.0093 | 0.0024 | 0.00888989 | 1.77E-14 | 0.2955 | 58.77777778 |
| rs7359097 | T | C | 0.0121 | 0.0117 | 0.0019 | 0.007196619 | 1.91E-10 | 0.104 | 40.5567867 |
| rs7367519 | T | C | -0.0164 | 0.0008 | 0.002 | 0.00762126 | 2.40E-16 | 0.9164 | 67.24 |
| rs74048171 | A | C | -0.0121 | 0.0122 | 0.0022 | 0.008114187 | 3.80E-08 | 0.1327 | 30.25 |
| rs7418410 | T | C | 0.0155 | -0.0215 | 0.0019 | 0.007134135 | 3.41E-16 | 0.002581 | 66.55124654 |
| rs7428883 | A | G | -0.0277 | -0.0035 | 0.0023 | 0.008775117 | 2.10E-33 | 0.69 | 145.0453686 |
| rs7448554 | A | C | -0.0132 | -0.0221 | 0.002 | 0.009891016 | 4.11E-11 | 0.02546 | 43.56 |
| rs7485647 | A | G | -0.0261 | -0.0174 | 0.0026 | 0.009542515 | 1.03E-23 | 0.06824 | 100.7707101 |
| rs75022676 | A | G | -0.0163 | -0.0017 | 0.0023 | 0.008887125 | 1.37E-12 | 0.8483 | 50.22495274 |
| rs75100513 | T | C | -0.0199 | -0.0006 | 0.0034 | 0.012053731 | 4.83E-09 | 0.9603 | 34.25692042 |
| rs75172776 | A | G | -0.0232 | -0.0202 | 0.0038 | 0.015475693 | 1.03E-09 | 0.1918 | 37.27423823 |
| rs7543136 | T | C | -0.021 | -0.0002 | 0.0021 | 0.010035619 | 1.52E-23 | 0.9841 | 100 |
| rs75702986 | A | G | -0.0163 | 0.0015 | 0.0025 | 0.009504603 | 7.03E-11 | 0.8746 | 42.5104 |
| rs757042 | T | C | 0.014 | -0.0027 | 0.0021 | 0.007817214 | 2.62E-11 | 0.7298 | 44.44444444 |
| rs7574162 | T | C | 0.0153 | 0.013 | 0.0022 | 0.008254957 | 3.54E-12 | 0.1153 | 48.36570248 |
| rs7582516 | T | C | 0.0254 | -0.0079 | 0.002 | 0.007674504 | 5.91E-37 | 0.3033 | 161.29 |
| rs7598430 | T | C | -0.016 | -0.0212 | 0.0019 | 0.007047674 | 3.73E-17 | 0.002629 | 70.91412742 |
| rs7633464 | A | G | 0.0175 | -0.0057 | 0.0019 | 0.007043992 | 3.25E-20 | 0.4184 | 84.83379501 |
| rs76364830 | A | G | -0.0471 | 0.0008 | 0.0039 | 0.016572983 | 1.40E-33 | 0.9615 | 145.852071 |
| rs7646501 | A | G | 0.017 | -0.0022 | 0.0021 | 0.007972968 | 5.72E-16 | 0.7826 | 65.53287982 |
| rs76488803 | A | G | -0.024 | -0.001 | 0.0034 | 0.013374862 | 1.68E-12 | 0.9404 | 49.82698962 |
| rs76520574 | T | C | -0.0449 | 0.0114 | 0.0049 | 0.01977058 | 5.04E-20 | 0.5642 | 83.96543107 |
| rs7666804 | T | C | 0.0167 | -0.005 | 0.0019 | 0.00974248 | 1.50E-18 | 0.6078 | 77.25484765 |
| rs76693355 | T | C | 0.0268 | -0.0093 | 0.003 | 0.010929028 | 4.13E-19 | 0.3948 | 79.80444444 |
| rs7692387 | A | G | 0.0169 | -0.0025 | 0.0024 | 0.008786249 | 1.90E-12 | 0.776 | 49.58506944 |
| rs7701233 | T | C | 0.0179 | 0.0145 | 0.0019 | 0.008285486 | 4.47E-21 | 0.08011 | 88.75623269 |
| rs7730092 | T | G | 0.0131 | 0.03 | 0.0024 | 0.008923259 | 4.81E-08 | 0.0007738 | 29.79340278 |
| rs7731023 | A | G | -0.0166 | -0.0015 | 0.0019 | 0.007401522 | 2.40E-18 | 0.8394 | 76.33240997 |
| rs77542162 | A | G | -0.0576 | -0.0236 | 0.0064 | 0.026424612 | 2.26E-19 | 0.3718 | 81 |
| rs7761910 | T | C | -0.016 | 0.0031 | 0.002 | 0.007828148 | 1.24E-15 | 0.6921 | 64 |
| rs7768382 | T | C | 0.0201 | -0.0052 | 0.0019 | 0.007352235 | 3.73E-26 | 0.4794 | 111.9141274 |
| rs7781964 | A | G | 0.0261 | 0.0098 | 0.0024 | 0.008904777 | 1.52E-27 | 0.2711 | 118.265625 |
| rs778384 | A | G | 0.0283 | -0.0102 | 0.0022 | 0.008521184 | 7.21E-38 | 0.2313 | 165.4731405 |
| rs78030362 | A | G | -0.0216 | -0.0035 | 0.0036 | 0.015372885 | 1.97E-09 | 0.8199 | 36 |
| rs7816345 | T | C | 0.0255 | 0.0242 | 0.0025 | 0.009161322 | 1.98E-24 | 0.008253 | 104.04 |
| rs78444298 | A | G | -0.0465 | 0.0444 | 0.0068 | 0.026929583 | 8.02E-12 | 0.0992 | 46.76146194 |
| rs78457529 | T | C | -0.0904 | -0.07 | 0.0088 | 0.031281986 | 9.35E-25 | 0.02524 | 105.5289256 |
| rs7858712 | A | G | -0.0347 | 0.0073 | 0.0034 | 0.012566455 | 1.87E-24 | 0.5613 | 104.1600346 |
| rs7893378 | A | G | 0.0175 | -0.0079 | 0.0031 | 0.013039066 | 1.65E-08 | 0.5446 | 31.86784599 |
| rs7910211 | T | C | -0.0175 | -0.0146 | 0.0026 | 0.009466363 | 1.69E-11 | 0.123 | 45.30325444 |
| rs7941305 | T | C | 0.0129 | 0.0351 | 0.0021 | 0.012562342 | 8.11E-10 | 0.005205 | 37.73469388 |
| rs79680939 | A | G | 0.029 | 0.0049 | 0.0046 | 0.019420409 | 2.89E-10 | 0.8008 | 39.74480151 |
| rs798528 | A | C | 0.0357 | 0.0159 | 0.002 | 0.007701216 | 2.89E-71 | 0.03896 | 318.6225 |
| rs8017006 | A | G | -0.0122 | -0.0193 | 0.002 | 0.010421722 | 1.06E-09 | 0.06404 | 37.21 |
| rs8018486 | A | G | 0.0138 | -0.0136 | 0.0024 | 0.008848563 | 8.92E-09 | 0.1243 | 33.0625 |
| rs8019890 | A | C | 0.025 | -0.0188 | 0.0019 | 0.007450925 | 1.53E-39 | 0.01163 | 173.1301939 |
| rs80280630 | T | C | -0.0168 | 0.0183 | 0.003 | 0.011540408 | 2.14E-08 | 0.1128 | 31.36 |
| rs8042545 | A | G | 0.0287 | -0.0019 | 0.0022 | 0.008084719 | 6.75E-39 | 0.8142 | 170.1838843 |
| rs8054549 | A | C | -0.0251 | -0.0166 | 0.0019 | 0.007154552 | 7.63E-40 | 0.02033 | 174.5180055 |
| rs8077636 | T | G | -0.0192 | 0.0038 | 0.0019 | 0.008632649 | 5.24E-24 | 0.6598 | 102.1163435 |
| rs8084413 | A | G | -0.0127 | 0.0122 | 0.0019 | 0.007454172 | 2.32E-11 | 0.1017 | 44.67867036 |
| rs8099461 | A | G | 0.0246 | -0.0016 | 0.0041 | 0.016215609 | 1.97E-09 | 0.9214 | 36 |
| rs8107967 | A | G | 0.0174 | 0.0132 | 0.0019 | 0.007400286 | 5.29E-20 | 0.07447 | 83.86703601 |
| rs8176632 | T | C | -0.0158 | -0.0111 | 0.0028 | 0.010104979 | 1.67E-08 | 0.272 | 31.84183673 |
| rs8180765 | A | G | 0.0151 | 0.0117 | 0.0023 | 0.008778563 | 5.20E-11 | 0.1826 | 43.1020794 |
| rs876122 | A | G | -0.0162 | -0.0443 | 0.0029 | 0.011454054 | 2.32E-08 | 0.0001099 | 31.20570749 |
| rs8904 | A | G | -0.0157 | 0.0119 | 0.002 | 0.00731329 | 4.16E-15 | 0.1037 | 61.6225 |
| rs894736 | A | G | 0.0174 | -0.0037 | 0.002 | 0.007321389 | 3.32E-18 | 0.6133 | 75.69 |
| rs921142 | T | C | 0.0111 | 0.0061 | 0.0019 | 0.007283403 | 5.15E-09 | 0.4023 | 34.13019391 |
| rs9344126 | T | C | 0.0185 | -0.0151 | 0.0019 | 0.00705955 | 2.10E-22 | 0.03244 | 94.80609418 |
| rs9376478 | A | G | -0.0186 | -0.0088 | 0.0022 | 0.008467847 | 2.80E-17 | 0.2987 | 71.47933884 |
| rs945508 | T | C | 0.0128 | -0.0178 | 0.0019 | 0.007051215 | 1.62E-11 | 0.01159 | 45.38504155 |
| rs9479012 | A | G | -0.0263 | -0.0172 | 0.0032 | 0.012323423 | 2.06E-16 | 0.1628 | 67.54785156 |
| rs9492799 | A | G | 0.0182 | 0.0026 | 0.0026 | 0.009338848 | 2.56E-12 | 0.7807 | 49 |
| rs9579402 | T | C | -0.0203 | -0.0132 | 0.0035 | 0.013034369 | 6.63E-09 | 0.3112 | 33.64 |
| rs9590328 | A | G | -0.0153 | -0.0323 | 0.0027 | 0.010163032 | 1.46E-08 | 0.001482 | 32.11111111 |
| rs9610447 | T | C | 0.0152 | -0.0061 | 0.0022 | 0.008287475 | 4.88E-12 | 0.4617 | 47.73553719 |
| rs963317 | A | G | 0.0136 | -0.0021 | 0.002 | 0.007303369 | 1.05E-11 | 0.7737 | 46.24 |
| rs9636364 | A | G | 0.011 | 0.0188 | 0.0019 | 0.007059622 | 7.06E-09 | 0.007744 | 33.51800554 |
| rs9659061 | A | G | -0.0198 | 0.0021 | 0.0019 | 0.007479889 | 1.99E-25 | 0.7789 | 108.598338 |
| rs9696116 | T | C | 0.0188 | 0.0114 | 0.002 | 0.00763686 | 5.46E-21 | 0.1355 | 88.36 |
| rs9784904 | T | C | 0.0217 | -0.0093 | 0.0026 | 0.009694416 | 7.05E-17 | 0.3374 | 69.65828402 |
| rs9807032 | T | C | 0.024 | 0.0339 | 0.0024 | 0.009072008 | 1.52E-23 | 0.0001864 | 100 |
| rs9809116 | A | G | 0.016 | 0.0015 | 0.0019 | 0.007458009 | 3.73E-17 | 0.8406 | 70.91412742 |
| rs9817452 | T | G | 0.0165 | -0.0032 | 0.0019 | 0.007235539 | 3.81E-18 | 0.6583 | 75.41551247 |
| rs9828525 | T | C | 0.0121 | 0.0137 | 0.0019 | 0.007159243 | 1.91E-10 | 0.05567 | 40.5567867 |
| rs9832919 | A | G | 0.0179 | -0.0123 | 0.002 | 0.007352792 | 3.55E-19 | 0.09436 | 80.1025 |
| rs9838614 | T | G | 0.0185 | -0.008 | 0.0019 | 0.007246481 | 2.10E-22 | 0.2696 | 94.80609418 |
| rs9853018 | T | C | 0.0469 | 0.0057 | 0.0019 | 0.00705005 | 1.58E-134 | 0.4188 | 609.3102493 |
| rs9894577 | A | G | -0.031 | 0.0124 | 0.002 | 0.007583013 | 3.47E-54 | 0.102 | 240.25 |
| rs9905385 | A | G | 0.0339 | 0.034 | 0.002 | 0.007493388 | 1.92E-64 | 0.0000057 | 287.3025 |
| rs9910161 | A | G | -0.0162 | 0.0216 | 0.0021 | 0.007804747 | 1.22E-14 | 0.005648 | 59.51020408 |
| rs9957318 | A | G | -0.0187 | -0.0008 | 0.002 | 0.00777051 | 8.76E-21 | 0.918 | 87.4225 |

**Supplementary Table 2** Genome-wide significant SNPs for the Association between Low hand grip as exposure and Knee osteoarthritis as outcome

| SNP | alt | ref | beta. exposure | beta. outcome | se. exposure | se. outcome | pval. exposure | pval. outcome | F |
| --- | --- | --- | --- | --- | --- | --- | --- | --- | --- |
| rs11236213 | A | G | -0.0504 | -0.0229 | 0.008 | 0.007740565 | 2.98E-10 | 0.003092 | 39.69 |
| rs12140813 | T | C | 0.0511 | 0.0026 | 0.0094 | 0.009129323 | 5.44E-08 | 0.7758 | 29.55194658 |
| rs143459567 | T | C | 0.1185 | 0.0195 | 0.0189 | 0.019511668 | 3.61E-10 | 0.3176 | 39.31090955 |
| rs79723785 | T | C | -0.1674 | -0.0098 | 0.0293 | 0.026594258 | 1.11E-08 | 0.7125 | 32.64191779 |

**Supplementary Table 3** Genome-wide significant SNPs for the Association between Usual walking pace as exposure and Knee osteoarthritis as outcome

| SNP | alt | ref | beta. exposure | beta. outcome | se. exposure | se. outcome | pval. exposure | se. outcome | F |
| --- | --- | --- | --- | --- | --- | --- | --- | --- | --- |
| rs10750025 | T | C | -0.0083554 | 0.0198 | 0.00136528 | 0.007683126 | 9.36E-10 | 0.007683126 | 37.45338156 |
| rs10862220 | G | T | 0.00840767 | 0.003 | 0.00135051 | 0.007483321 | 4.80E-10 | 0.007483321 | 38.75749557 |
| rs11077815 | C | T | -0.00713336 | 0.0057 | 0.00130691 | 0.007257148 | 4.81E-08 | 0.007257148 | 29.79181224 |
| rs11152989 | T | C | -0.00751226 | -0.0024 | 0.00136699 | 0.007700734 | 3.90E-08 | 0.007700734 | 30.20025054 |
| rs11682482 | G | T | 0.00801894 | 0.0028 | 0.00135595 | 0.007524359 | 3.34E-09 | 0.007524359 | 34.97410239 |
| rs11848096 | C | T | -0.00750831 | -0.0044 | 0.00130696 | 0.00726407 | 9.20E-09 | 0.00726407 | 33.00348477 |
| rs144333966 | G | A | 0.0304614 | -0.0488 | 0.00544615 | 0.028348268 | 2.23E-08 | 0.028348268 | 31.28387355 |
| rs2037735 | T | C | -0.0112677 | 0.0072 | 0.00194308 | 0.010848726 | 6.68E-09 | 0.010848726 | 33.62708237 |
| rs2170670 | A | G | -0.00708769 | 0.0132 | 0.0012972 | 0.007214961 | 4.66E-08 | 0.007214961 | 29.85352018 |
| rs2602731 | G | A | -0.00766675 | 0.0111 | 0.00136907 | 0.007725977 | 2.14E-08 | 0.007725977 | 31.35965641 |
| rs2645979 | A | G | 0.00861798 | -0.0091 | 0.00132024 | 0.007313522 | 6.68E-11 | 0.007313522 | 42.6093776 |
| rs4109292 | A | G | 0.00735336 | -0.0144 | 0.00126787 | 0.007353162 | 6.64E-09 | 0.007353162 | 33.63738338 |
| rs6763292 | G | A | 0.00958833 | 0.0055 | 0.00153076 | 0.008637869 | 3.76E-10 | 0.008637869 | 39.23482636 |
| rs7789719 | C | T | 0.00854199 | -0.0025 | 0.00153719 | 0.008381774 | 2.75E-08 | 0.008381774 | 30.87898498 |

**Supplementary Table 4** Genome-wide significant SNPs for the Association between Appendicular lean mass as exposure and Hip osteoarthritis as outcome

| SNP | alt | ref | beta. exposure | beta. outcome | se. exposure | se. outcome | pval. exposure | pval. outcome | F |
| --- | --- | --- | --- | --- | --- | --- | --- | --- | --- |
| rs10008637 | T | C | 0.013 | 0.0028 | 0.0019 | 0.009060659 | 7.80E-12 | 0.7573 | 46.81440443 |
| rs10019221 | T | G | -0.0124 | -0.01 | 0.0019 | 0.009374564 | 6.74E-11 | 0.2861 | 42.59279778 |
| rs10040039 | A | G | 0.0163 | 0.0039 | 0.0019 | 0.00922 | 9.57E-18 | 0.6723 | 73.59833795 |
| rs10041978 | A | G | -0.0172 | -0.0281 | 0.0019 | 0.009140724 | 1.40E-19 | 0.002111 | 81.9501385 |
| rs1004982 | T | C | 0.0117 | -0.0017 | 0.002 | 0.009276824 | 4.92E-09 | 0.8546 | 34.2225 |
| rs10058744 | A | G | 0.0146 | 0.0082 | 0.0019 | 0.00905576 | 1.54E-14 | 0.3652 | 59.04709141 |
| rs10099846 | T | C | 0.0116 | 0.0016 | 0.002 | 0.009768874 | 6.63E-09 | 0.8699 | 33.64 |
| rs10112506 | A | G | 0.012 | 0.0193 | 0.0019 | 0.009342754 | 2.69E-10 | 0.03885 | 39.88919668 |
| rs10119967 | A | C | -0.0353 | -0.0166 | 0.0023 | 0.011100422 | 3.66E-53 | 0.1348 | 235.5557656 |
| rs10128781 | T | C | -0.0172 | -0.0159 | 0.0021 | 0.009959923 | 2.60E-16 | 0.1104 | 67.08390023 |
| rs1014526 | T | C | 0.0138 | 0.0116 | 0.002 | 0.009257665 | 5.20E-12 | 0.2102 | 47.61 |
| rs10176654 | T | C | 0.0128 | -0.0133 | 0.0023 | 0.010926053 | 2.62E-08 | 0.2235 | 30.97164461 |
| rs10202701 | T | C | 0.0227 | -0.0002 | 0.0019 | 0.008914163 | 6.70E-33 | 0.9821 | 142.7396122 |
| rs10209278 | T | C | 0.0141 | -0.0225 | 0.002 | 0.009617474 | 1.79E-12 | 0.01931 | 49.7025 |
| rs10222594 | A | G | 0.0115 | -0.0114 | 0.002 | 0.009895393 | 8.92E-09 | 0.2493 | 33.0625 |
| rs10241451 | T | C | -0.0145 | -0.0036 | 0.0023 | 0.011368585 | 2.89E-10 | 0.7515 | 39.74480151 |
| rs10283100 | A | G | -0.0575 | -0.036 | 0.0041 | 0.01961451 | 1.11E-44 | 0.06645 | 196.6835217 |
| rs1035583 | A | G | 0.0148 | 0.0097 | 0.0019 | 0.009270331 | 6.73E-15 | 0.2954 | 60.67590028 |
| rs1040977 | T | C | -0.024 | -0.0109 | 0.0025 | 0.012358878 | 7.99E-22 | 0.3778 | 92.16 |
| rs10421750 | T | G | -0.0145 | 0.0124 | 0.0021 | 0.014282879 | 5.03E-12 | 0.3853 | 47.67573696 |
| rs10491967 | A | G | 0.0471 | 0.0066 | 0.0032 | 0.015107201 | 4.89E-49 | 0.6622 | 216.6416016 |
| rs10514518 | T | C | 0.0171 | -0.0021 | 0.002 | 0.009642729 | 1.23E-17 | 0.8276 | 73.1025 |
| rs1051952 | A | C | -0.0133 | 0.0122 | 0.0019 | 0.009792094 | 2.56E-12 | 0.2128 | 49 |
| rs1056747 | A | G | 0.0155 | -0.002 | 0.0019 | 0.008941021 | 3.41E-16 | 0.823 | 66.55124654 |
| rs1063582 | T | G | 0.0185 | 0.0001 | 0.0022 | 0.007598681 | 4.13E-17 | 0.9895 | 70.71280992 |
| rs10736029 | T | G | -0.0193 | 0.0116 | 0.0035 | 0.019503474 | 3.50E-08 | 0.552 | 30.40734694 |
| rs10748128 | T | G | 0.0255 | 0.0315 | 0.002 | 0.009375371 | 3.12E-37 | 0.0007798 | 162.5625 |
| rs10749157 | T | C | -0.0113 | -0.0007 | 0.002 | 0.010146845 | 1.60E-08 | 0.945 | 31.9225 |
| rs10764692 | A | C | 0.0111 | 0.0202 | 0.002 | 0.009440629 | 2.86E-08 | 0.03238 | 30.8025 |
| rs10776560 | T | C | -0.0157 | -0.0153 | 0.0019 | 0.009004476 | 1.42E-16 | 0.08929 | 68.27977839 |
| rs10779958 | A | C | -0.0157 | -0.0126 | 0.0026 | 0.012825658 | 1.56E-09 | 0.3259 | 36.46301775 |
| rs10796828 | T | G | -0.0154 | -0.004 | 0.002 | 0.00985081 | 1.36E-14 | 0.6847 | 59.29 |
| rs10810474 | T | C | 0.0143 | -0.0064 | 0.0019 | 0.009123494 | 5.22E-14 | 0.483 | 56.64542936 |
| rs10815304 | T | C | -0.0151 | 0.0154 | 0.0023 | 0.011320111 | 5.20E-11 | 0.1737 | 43.1020794 |
| rs10822117 | A | G | 0.0176 | -0.0103 | 0.0022 | 0.010648326 | 1.24E-15 | 0.3334 | 64 |
| rs10827415 | T | G | 0.0142 | -0.0105 | 0.0021 | 0.010204511 | 1.36E-11 | 0.3035 | 45.72335601 |
| rs10832963 | T | G | 0.0203 | 0.0216 | 0.0022 | 0.010186625 | 2.78E-20 | 0.03397 | 85.14256198 |
| rs10840399 | T | C | -0.0209 | 0.0007 | 0.0031 | 0.015952572 | 1.56E-11 | 0.965 | 45.45369407 |
| rs10849576 | T | G | 0.0117 | 0.0159 | 0.0019 | 0.009188364 | 7.37E-10 | 0.08355 | 37.91966759 |
| rs10880272 | T | G | 0.0138 | 0.0305 | 0.0019 | 0.009264585 | 3.78E-13 | 0.0009944 | 52.7534626 |
| rs10883555 | T | C | 0.0254 | 0.0069 | 0.0019 | 0.009164361 | 9.25E-41 | 0.4515 | 178.7146814 |
| rs10917335 | A | G | 0.0198 | -0.0248 | 0.002 | 0.013851189 | 4.16E-23 | 0.07338 | 98.01 |
| rs10922476 | A | G | -0.016 | -0.007 | 0.0019 | 0.009035421 | 3.73E-17 | 0.4385 | 70.91412742 |
| rs1093086 | T | C | -0.0129 | 0.0042 | 0.0022 | 0.010494425 | 4.53E-09 | 0.689 | 34.3822314 |
| rs10995566 | T | C | -0.0123 | -0.0381 | 0.002 | 0.009736864 | 7.75E-10 | 0.0000912 | 37.8225 |
| rs11021305 | T | C | -0.0152 | -0.0255 | 0.0019 | 0.009350704 | 1.24E-15 | 0.00639 | 64 |
| rs11042717 | T | C | 0.029 | 0.0068 | 0.0019 | 0.009065569 | 1.35E-52 | 0.4532 | 232.9639889 |
| rs11132166 | T | C | 0.0292 | -0.0226 | 0.005 | 0.024216905 | 5.22E-09 | 0.3507 | 34.1056 |
| rs11158820 | A | G | 0.0235 | 0.0031 | 0.0021 | 0.009950983 | 4.54E-29 | 0.7554 | 125.2267574 |
| rs111622870 | T | C | 0.0282 | 0.0427 | 0.0044 | 0.022296508 | 1.46E-10 | 0.05548 | 41.07644628 |
| rs11175919 | A | G | 0.0349 | 0.024 | 0.0059 | 0.027702532 | 3.31E-09 | 0.3863 | 34.99023269 |
| rs11187838 | A | G | 0.0394 | -0.0104 | 0.0019 | 0.009073223 | 1.61E-95 | 0.2517 | 430.0166205 |
| rs111901094 | T | G | -0.0253 | -0.0014 | 0.0025 | 0.012682296 | 4.50E-24 | 0.9121 | 102.4144 |
| rs11191208 | A | G | 0.0147 | 0.0062 | 0.0024 | 0.011527979 | 9.07E-10 | 0.5907 | 37.515625 |
| rs11198591 | A | G | 0.0148 | 0.0004 | 0.002 | 0.010033626 | 1.36E-13 | 0.9682 | 54.76 |
| rs11210229 | A | G | -0.0125 | -0.008 | 0.0019 | 0.009193461 | 4.74E-11 | 0.3842 | 43.28254848 |
| rs112369231 | T | C | -0.0181 | -0.0176 | 0.0021 | 0.013302029 | 6.75E-18 | 0.1858 | 74.28798186 |
| rs112373502 | T | C | -0.0179 | -0.0259 | 0.0028 | 0.012847833 | 1.63E-10 | 0.04381 | 40.86862245 |
| rs112537273 | T | C | 0.0212 | -0.014 | 0.0022 | 0.01076679 | 5.61E-22 | 0.1935 | 92.85950413 |
| rs112873218 | T | C | 0.0216 | -0.0119 | 0.0031 | 0.014810658 | 3.22E-12 | 0.4217 | 48.54942768 |
| rs113107560 | T | G | 0.019 | 0.0166 | 0.0019 | 0.013749197 | 1.52E-23 | 2.27E-01 | 100 |
| rs113289555 | T | G | -0.0206 | -0.0258 | 0.0023 | 0.015240853 | 3.35E-19 | 0.09049 | 80.21928166 |
| rs113671109 | T | C | 0.015 | 0.0076 | 0.0023 | 0.010883834 | 6.95E-11 | 0.485 | 42.53308129 |
| rs113827862 | T | C | 0.0235 | 0.0177 | 0.004 | 0.018508043 | 4.23E-09 | 0.3389 | 34.515625 |
| rs113852999 | A | G | 0.0246 | 0.0091 | 0.0025 | 0.012097023 | 7.57E-23 | 0.4519 | 96.8256 |
| rs114018835 | T | C | 0.0303 | -0.0279 | 0.0051 | 0.024438337 | 2.83E-09 | 0.2536 | 35.29757785 |
| rs114192718 | T | C | -0.0215 | -0.0067 | 0.0036 | 0.017566458 | 2.34E-09 | 0.7029 | 35.66743827 |
| rs114299654 | T | C | 0.0304 | -0.0624 | 0.0051 | 0.026407642 | 2.51E-09 | 0.01813 | 35.53094963 |
| rs115233595 | T | C | 0.0264 | 0.002 | 0.0039 | 0.018433377 | 1.29E-11 | 0.9136 | 45.82248521 |
| rs115912456 | A | G | -0.0577 | 0.0391 | 0.0047 | 0.022412418 | 1.21E-34 | 0.08106 | 150.7148031 |
| rs116008080 | A | G | -0.0415 | -0.0412 | 0.0063 | 0.029301294 | 4.48E-11 | 0.1597 | 43.3925422 |
| rs11605297 | A | G | 0.0146 | 0.0009 | 0.0022 | 0.010547436 | 3.22E-11 | 0.932 | 44.04132231 |
| rs11633371 | T | G | 0.0216 | 0.0018 | 0.0019 | 0.009201056 | 6.01E-30 | 0.8449 | 129.2409972 |
| rs116339650 | A | G | 0.0175 | -0.0116 | 0.0029 | 0.013779998 | 1.59E-09 | 0.3999 | 36.41498216 |
| rs116493405 | A | G | 0.0287 | -0.012 | 0.0042 | 0.01972278 | 8.30E-12 | 0.5429 | 46.69444444 |
| rs11651280 | T | G | 0.0267 | -0.0017 | 0.004 | 0.01845351 | 2.47E-11 | 0.9266 | 44.555625 |
| rs11672848 | T | C | -0.0171 | 0.0086 | 0.0019 | 0.010733532 | 2.26E-19 | 0.423 | 81 |
| rs11684531 | A | G | 0.0172 | 0.0077 | 0.0028 | 0.016082772 | 8.11E-10 | 0.6321 | 37.73469388 |
| rs11689546 | A | G | 0.0238 | -0.0023 | 0.0019 | 0.011299068 | 5.36E-36 | 0.8387 | 156.9085873 |
| rs117068593 | T | C | 0.0403 | 0.003 | 0.0024 | 0.012565483 | 2.81E-63 | 0.8113 | 281.9600694 |
| rs117203652 | A | G | -0.0346 | -0.0264 | 0.0055 | 0.028997542 | 3.16E-10 | 0.3626 | 39.57553719 |
| rs11720869 | A | G | 0.0141 | 0.012 | 0.002 | 0.0095601 | 1.79E-12 | 0.2094 | 49.7025 |
| rs11721522 | A | G | -0.0106 | -0.0121 | 0.0019 | 0.009186744 | 2.42E-08 | 0.1878 | 31.12465374 |
| rs1177765 | T | C | 0.0232 | 0.0032 | 0.0019 | 0.009110293 | 2.73E-34 | 0.7254 | 149.0969529 |
| rs11777835 | T | C | 0.0152 | -0.0367 | 0.0019 | 0.009179675 | 1.24E-15 | 0.0000639 | 64 |
| rs117818446 | A | G | 0.0423 | 0.055 | 0.0068 | 0.034093623 | 4.95E-10 | 0.1067 | 38.69571799 |
| rs11927331 | A | C | -0.0147 | -0.0154 | 0.002 | 0.009540764 | 1.98E-13 | 0.1065 | 54.0225 |
| rs11991823 | A | G | -0.0161 | -0.0094 | 0.002 | 0.012716859 | 8.28E-16 | 0.4598 | 64.8025 |
| rs12037677 | T | C | 0.0178 | 0.0069 | 0.0021 | 0.011689281 | 2.33E-17 | 0.555 | 71.84580499 |
| rs12055045 | T | C | 0.0213 | 0.0157 | 0.0023 | 0.010941112 | 2.03E-20 | 0.1513 | 85.7637051 |
| rs1211575 | A | G | -0.0237 | -0.0122 | 0.0021 | 0.010677297 | 1.54E-29 | 0.2532 | 127.3673469 |
| rs1216743 | A | G | -0.0152 | -0.0281 | 0.0021 | 0.010010797 | 4.55E-13 | 0.005001 | 52.39002268 |
| rs12212816 | T | C | 0.0162 | 0.0563 | 0.0019 | 0.008949693 | 1.51E-17 | 3.16E-10 | 72.69806094 |
| rs12299065 | A | G | -0.024 | -0.0056 | 0.0028 | 0.013209008 | 1.02E-17 | 0.6716 | 73.46938776 |
| rs12325539 | T | C | -0.0275 | -0.0225 | 0.0019 | 0.009166069 | 1.78E-47 | 0.0141 | 209.4875346 |
| rs12340775 | A | G | -0.0287 | 0.0256 | 0.0043 | 0.020790551 | 2.48E-11 | 0.2182 | 44.54786371 |
| rs12344515 | T | C | -0.0163 | -0.0236 | 0.0022 | 0.010530572 | 1.27E-13 | 0.02502 | 54.8946281 |
| rs12351226 | T | C | 0.0218 | 0.0134 | 0.0025 | 0.012308611 | 2.78E-18 | 0.2763 | 76.0384 |
| rs12371664 | A | G | 0.0168 | 0.0047 | 0.0021 | 0.010396028 | 1.24E-15 | 0.6512 | 64 |
| rs12463908 | A | C | 0.0165 | 0.0017 | 0.0024 | 0.012147251 | 6.20E-12 | 0.8887 | 47.265625 |
| rs12474969 | A | G | 0.0167 | 0.0099 | 0.002 | 0.010694567 | 6.83E-17 | 3.55E-01 | 69.7225 |
| rs12483401 | T | C | 0.0387 | 0.0561 | 0.0067 | 0.032381522 | 7.64E-09 | 0.08319 | 33.36355536 |
| rs12509014 | T | C | -0.0263 | -0.0037 | 0.0023 | 0.011083141 | 2.80E-30 | 0.7385 | 130.7542533 |
| rs12512942 | A | G | -0.0162 | -0.0188 | 0.002 | 0.009267966 | 5.50E-16 | 0.04251 | 65.61 |
| rs12533452 | T | C | 0.0237 | -0.0164 | 0.0026 | 0.012038374 | 7.84E-20 | 0.1731 | 83.09023669 |
| rs12595051 | A | G | 0.0179 | 0.0101 | 0.0021 | 0.009995965 | 1.54E-17 | 0.3123 | 72.6553288 |
| rs12612857 | A | G | -0.0124 | -0.0112 | 0.0022 | 0.010464697 | 1.74E-08 | 0.2845 | 31.76859504 |
| rs12622189 | A | G | 0.0272 | 0.0106 | 0.0021 | 0.010180925 | 2.28E-38 | 0.2978 | 167.7641723 |
| rs12655296 | T | C | -0.011 | -0.0001 | 0.002 | 0.013997856 | 3.80E-08 | 0.9943 | 30.25 |
| rs12662115 | A | G | 0.0152 | -0.0119 | 0.002 | 0.009790729 | 2.96E-14 | 0.2242 | 57.76 |
| rs12663031 | T | C | -0.0195 | -0.0185 | 0.0019 | 0.010723345 | 1.03E-24 | 0.08449 | 105.33241 |
| rs12679359 | T | G | -0.0263 | -0.0726 | 0.0028 | 0.013390298 | 5.84E-21 | 0.000000059 | 88.22576531 |
| rs12700901 | A | C | -0.0184 | -0.013 | 0.0019 | 0.009161403 | 3.52E-22 | 0.1559 | 93.78393352 |
| rs12702693 | T | C | 0.0173 | 0.0176 | 0.0019 | 0.009069098 | 8.61E-20 | 0.0523 | 82.90581717 |
| rs12713004 | A | G | -0.0367 | 0.0162 | 0.0021 | 0.010221776 | 2.18E-68 | 0.113 | 305.4172336 |
| rs12714414 | T | C | 0.0353 | 0.0471 | 0.0027 | 0.013743462 | 4.63E-39 | 0.0006101 | 170.9314129 |
| rs12761076 | A | G | -0.026 | 0.0041 | 0.0021 | 0.010006456 | 3.31E-35 | 6.82E-01 | 153.2879819 |
| rs12894822 | A | G | -0.0135 | -0.0086 | 0.0022 | 0.010224881 | 8.44E-10 | 0.4003 | 37.65495868 |
| rs12907384 | T | C | 0.0269 | -0.0004 | 0.0019 | 0.008243634 | 1.67E-45 | 0.9613 | 200.4459834 |
| rs1290786 | T | C | -0.0143 | -0.0061 | 0.0019 | 0.009154619 | 5.22E-14 | 0.5052 | 56.64542936 |
| rs12997625 | T | C | -0.017 | -0.0039 | 0.0019 | 0.009064098 | 3.64E-19 | 0.667 | 80.05540166 |
| rs13109280 | A | G | -0.0131 | -0.0031 | 0.002 | 0.009867603 | 5.75E-11 | 0.7534 | 42.9025 |
| rs13112742 | A | G | -0.0151 | -0.0024 | 0.0025 | 0.012180565 | 1.54E-09 | 0.8438 | 36.4816 |
| rs13127468 | A | C | -0.0123 | 0.0015 | 0.0019 | 0.009383854 | 9.56E-11 | 0.873 | 41.90858726 |
| rs13170063 | A | G | -0.0152 | -0.0139 | 0.0019 | 0.012106262 | 1.24E-15 | 0.2509 | 64 |
| rs1317349 | T | G | -0.0258 | -0.0058 | 0.0021 | 0.010087182 | 1.08E-34 | 0.5653 | 150.9387755 |
| rs13193017 | A | G | 0.0164 | 0.024 | 0.0027 | 0.013008734 | 1.25E-09 | 0.06505 | 36.89437586 |
| rs13209574 | T | G | -0.0292 | 0.0221 | 0.0032 | 0.014990133 | 7.17E-20 | 0.1404 | 83.265625 |
| rs13209685 | T | G | 0.0277 | 0.0316 | 0.0026 | 0.012004913 | 1.67E-26 | 0.008482 | 113.5044379 |
| rs1325596 | A | G | 0.0287 | 0.011 | 0.0019 | 0.009105034 | 1.50E-51 | 0.227 | 228.1689751 |
| rs13316 | A | C | 0.0115 | 0.0009 | 0.0019 | 0.008972387 | 1.42E-09 | 0.9201 | 36.63434903 |
| rs13321258 | A | G | 0.0133 | 0.0168 | 0.0022 | 0.010729473 | 1.49E-09 | 0.1174 | 36.54752066 |
| rs1341215 | A | G | 0.0229 | 0.0285 | 0.0027 | 0.012882863 | 2.22E-17 | 0.02695 | 71.93552812 |
| rs1355603 | T | C | -0.0466 | -0.0195 | 0.0025 | 0.012280064 | 1.52E-77 | 0.1123 | 347.4496 |
| rs139921635 | T | G | 0.0385 | -0.0456 | 0.0062 | 0.03173915 | 5.31E-10 | 0.1508 | 38.56009365 |
| rs1405227 | A | G | 0.0129 | 0.0114 | 0.002 | 0.009816171 | 1.12E-10 | 0.2455 | 41.6025 |
| rs141277904 | T | C | 0.0384 | -0.0523 | 0.0069 | 0.035742445 | 2.62E-08 | 0.1434 | 30.97164461 |
| rs1430157 | T | C | 0.0182 | -0.0068 | 0.002 | 0.009791921 | 9.03E-20 | 0.4874 | 82.81 |
| rs143076454 | A | G | -0.0499 | -0.0604 | 0.007 | 0.034096837 | 1.01E-12 | 0.07649 | 50.81653061 |
| rs1436164 | T | C | -0.0139 | 0.0126 | 0.0019 | 0.008995421 | 2.56E-13 | 0.1613 | 53.52077562 |
| rs144109601 | A | C | -0.0278 | 0.0022 | 0.0048 | 0.023184064 | 6.97E-09 | 0.9244 | 33.54340278 |
| rs1444628 | T | C | 0.024 | 0.0033 | 0.002 | 0.009662228 | 3.55E-33 | 0.7327 | 144 |
| rs1447691 | A | G | 0.0181 | -0.0046 | 0.002 | 0.009562948 | 1.43E-19 | 0.6305 | 81.9025 |
| rs145147649 | A | G | -0.0362 | 0.0048 | 0.0045 | 0.022369976 | 8.66E-16 | 0.8301 | 64.71308642 |
| rs147110934 | T | G | -0.0722 | 0.0632 | 0.0062 | 0.033084536 | 2.43E-31 | 5.61E-02 | 135.6097815 |
| rs1473441 | A | G | 0.0198 | 0.0001 | 0.0021 | 0.008141474 | 4.16E-21 | 0.9902 | 88.89795918 |
| rs1487441 | A | G | 0.0136 | 0.0089 | 0.0019 | 0.008986665 | 8.19E-13 | 0.322 | 51.23545706 |
| rs149697773 | A | G | 0.0262 | -0.0199 | 0.0047 | 0.023821303 | 2.48E-08 | 0.4035 | 31.07469443 |
| rs1557341 | A | C | -0.0153 | -0.0135 | 0.002 | 0.009631051 | 2.01E-14 | 0.161 | 58.5225 |
| rs165849 | A | G | 0.0157 | 0.0106 | 0.0021 | 0.012956849 | 7.65E-14 | 0.4133 | 55.89342404 |
| rs1662842 | A | G | -0.0214 | -0.0494 | 0.002 | 0.009711852 | 1.02E-26 | 0.000000365 | 114.49 |
| rs16844417 | A | G | 0.0226 | 0.0347 | 0.0029 | 0.013862639 | 6.54E-15 | 0.01231 | 60.73246136 |
| rs17036160 | T | C | -0.0375 | 0.0096 | 0.0029 | 0.013481165 | 3.01E-38 | 0.4764 | 167.2116528 |
| rs17197114 | T | C | -0.0177 | -0.0064 | 0.0025 | 0.012728543 | 1.44E-12 | 0.6151 | 50.1264 |
| rs17205463 | T | C | -0.0263 | -0.0077 | 0.0019 | 0.009052595 | 1.42E-43 | 0.395 | 191.6038781 |
| rs17428810 | T | C | 0.0155 | 0.0311 | 0.002 | 0.010054748 | 9.19E-15 | 0.001981 | 60.0625 |
| rs17478946 | A | G | 0.0192 | 0.0216 | 0.0021 | 0.010015358 | 6.08E-20 | 0.03103 | 83.59183673 |
| rs17496249 | A | G | -0.0123 | -0.0037 | 0.0019 | 0.009235687 | 9.56E-11 | 0.6887 | 41.90858726 |
| rs17681189 | A | C | -0.0131 | 0.0071 | 0.0019 | 0.009126581 | 5.40E-12 | 0.4366 | 47.53739612 |
| rs17713523 | A | G | 0.0114 | -0.0004 | 0.0019 | 0.009439596 | 1.97E-09 | 0.9662 | 36 |
| rs17773965 | T | C | -0.0163 | -0.0056 | 0.0027 | 0.01269392 | 1.57E-09 | 0.6591 | 36.44581619 |
| rs1805165 | A | C | -0.0184 | -0.026 | 0.0021 | 0.010085419 | 1.92E-18 | 0.009938 | 76.77097506 |
| rs1809179 | T | C | -0.0155 | 0.0138 | 0.0026 | 0.012713028 | 2.50E-09 | 0.2777 | 35.53994083 |
| rs181766 | T | C | -0.0221 | -0.0346 | 0.002 | 0.009762601 | 2.19E-28 | 0.0003939 | 122.1025 |
| rs1899040 | T | C | 0.0152 | -0.0109 | 0.0023 | 0.011194285 | 3.88E-11 | 0.3302 | 43.67485822 |
| rs190801170 | A | G | 0.0286 | 0.024 | 0.0036 | 0.021454904 | 1.95E-15 | 0.2633 | 63.11419753 |
| rs1977337 | T | C | -0.0186 | -0.0102 | 0.0026 | 0.012542948 | 8.44E-13 | 0.4161 | 51.17751479 |
| rs200439 | A | C | 0.0128 | 0.0052 | 0.0023 | 0.010957498 | 2.62E-08 | 0.6351 | 30.97164461 |
| rs2007022 | A | C | 0.0179 | -0.0041 | 0.0022 | 0.011061719 | 4.07E-16 | 0.7109 | 66.20041322 |
| rs200739311 | T | C | 0.0128 | 0.0373 | 0.002 | 0.013635122 | 1.55E-10 | 0.006227 | 40.96 |
| rs200776140 | A | G | -0.015 | -0.0063 | 0.0027 | 0.018561557 | 2.77E-08 | 0.7343 | 30.86419753 |
| rs201570119 | T | C | -0.0194 | 0.0227 | 0.0023 | 0.014331058 | 3.32E-17 | 0.1132 | 71.14555766 |
| rs201764844 | T | C | -0.0196 | 0.0131 | 0.0021 | 0.014266624 | 1.03E-20 | 0.3585 | 87.11111111 |
| rs2019203 | A | C | 0.0189 | 0.0044 | 0.0019 | 0.009080707 | 2.59E-23 | 0.628 | 98.9501385 |
| rs2052478 | T | C | -0.0195 | -0.018 | 0.0022 | 0.010803559 | 7.74E-19 | 0.09569 | 78.56404959 |
| rs2070598 | A | G | 0.0204 | 0.0122 | 0.0019 | 0.009022097 | 6.83E-27 | 0.1763 | 115.2797784 |
| rs2071518 | T | C | -0.0236 | 0.0097 | 0.0021 | 0.010244172 | 2.65E-29 | 0.3437 | 126.2947846 |
| rs2098695 | A | G | 0.0255 | -0.0108 | 0.002 | 0.009752145 | 3.12E-37 | 0.2681 | 162.5625 |
| rs2112617 | A | G | -0.0167 | 0.0122 | 0.0019 | 0.00895746 | 1.50E-18 | 0.1732 | 77.25484765 |
| rs2125125 | T | C | -0.0158 | -0.0183 | 0.0023 | 0.01102989 | 6.44E-12 | 0.09709 | 47.19092628 |
| rs212526 | T | C | -0.0214 | -0.0065 | 0.0019 | 0.009167332 | 1.99E-29 | 0.4783 | 126.8587258 |
| rs2126942 | T | C | 0.0127 | -0.0136 | 0.0019 | 0.009159557 | 2.32E-11 | 0.1376 | 44.67867036 |
| rs2138374 | T | C | 0.0149 | 0.0442 | 0.002 | 0.009679333 | 9.33E-14 | 0.00000496 | 55.5025 |
| rs2140046 | T | C | 0.0192 | -0.0093 | 0.0019 | 0.009288275 | 5.24E-24 | 0.3167 | 102.1163435 |
| rs2142644 | A | C | -0.0181 | 0.022 | 0.002 | 0.012173481 | 1.43E-19 | 0.07073 | 81.9025 |
| rs2165772 | A | G | 0.016 | 0.0108 | 0.002 | 0.009440089 | 1.24E-15 | 0.2526 | 64 |
| rs2208404 | A | G | 0.0111 | -0.0043 | 0.0019 | 0.009674359 | 5.15E-09 | 0.6567 | 34.13019391 |
| rs2209098 | T | C | -0.024 | 0.0071 | 0.002 | 0.00963317 | 3.55E-33 | 0.4611 | 144 |
| rs2212926 | A | C | -0.022 | 0.0023 | 0.0023 | 0.011575431 | 1.12E-21 | 0.8425 | 91.49338374 |
| rs2252031 | T | C | 0.0176 | -0.0039 | 0.0026 | 0.012844037 | 1.29E-11 | 0.7614 | 45.82248521 |
| rs2280463 | A | G | 0.0147 | 0.011 | 0.0021 | 0.009947277 | 2.56E-12 | 0.2688 | 49 |
| rs2287821 | T | C | -0.0153 | 0.0031 | 0.0019 | 0.008930589 | 8.10E-16 | 0.7285 | 64.84487535 |
| rs2289976 | A | G | 0.0144 | 0.0078 | 0.002 | 0.009902844 | 6.02E-13 | 0.4309 | 51.84 |
| rs2296316 | T | C | 0.0192 | 0.0178 | 0.0019 | 0.009173324 | 5.24E-24 | 0.05233 | 102.1163435 |
| rs2298333 | T | C | -0.0267 | 0.0025 | 0.0019 | 0.009382959 | 7.42E-45 | 0.7899 | 197.4764543 |
| rs2303423 | T | C | -0.0168 | -0.0178 | 0.003 | 0.014534541 | 2.14E-08 | 0.2207 | 31.36 |
| rs2304655 | T | C | -0.0113 | 0.0127 | 0.0019 | 0.009194525 | 2.72E-09 | 0.1672 | 35.37119114 |
| rs2305141 | A | G | -0.0183 | 0.0031 | 0.0019 | 0.009112076 | 5.88E-22 | 0.7337 | 92.76731302 |
| rs2324154 | A | C | 0.015 | -0.0147 | 0.0019 | 0.008992162 | 2.91E-15 | 0.1021 | 62.32686981 |
| rs2347808 | A | G | -0.0125 | -0.0129 | 0.0019 | 0.009174434 | 4.74E-11 | 0.1597 | 43.28254848 |
| rs2348496 | A | G | 0.0136 | 0.0086 | 0.0019 | 0.01326575 | 8.19E-13 | 0.5168 | 51.23545706 |
| rs2436772 | A | G | 0.0222 | -0.0115 | 0.0023 | 0.011227328 | 4.81E-22 | 0.3057 | 93.16446125 |
| rs2487 | T | C | -0.014 | -0.0041 | 0.0019 | 0.009258959 | 1.73E-13 | 0.6579 | 54.29362881 |
| rs2506697 | A | G | 0.0141 | -0.0005 | 0.002 | 0.009969377 | 1.79E-12 | 0.96 | 49.7025 |
| rs2531991 | A | G | 0.0189 | 0.0064 | 0.0022 | 0.010589601 | 8.63E-18 | 0.5456 | 73.80371901 |
| rs2539251 | T | G | -0.0162 | -2.39E-02 | 0.0027 | 0.013295019 | 1.97E-09 | 0.07223 | 36 |
| rs2549677 | A | G | 0.0392 | 0.0214 | 0.0032 | 0.019239044 | 1.68E-34 | 0.266 | 150.0625 |
| rs2569888 | A | G | 0.0133 | 0.0073 | 0.0022 | 0.010628789 | 1.49E-09 | 0.4922 | 36.54752066 |
| rs2578565 | T | C | -0.0141 | -0.0054 | 0.002 | 0.009415717 | 1.79E-12 | 0.5663 | 49.7025 |
| rs2596144 | T | C | 0.0223 | -0.0039 | 0.0028 | 0.012883011 | 1.66E-15 | 0.7621 | 63.42984694 |
| rs2607234 | A | G | 0.0302 | -0.013 | 0.0043 | 0.020304077 | 2.17E-12 | 0.522 | 49.32612223 |
| rs2609334 | T | C | 0.0171 | 0.0106 | 0.0022 | 0.010584449 | 7.68E-15 | 0.3166 | 60.41528926 |
| rs261999 | T | C | 0.0175 | 0.0122 | 0.0019 | 0.009258471 | 3.25E-20 | 0.1876 | 84.83379501 |
| rs2627702 | T | C | 0.0191 | 0.0053 | 0.0019 | 0.009017437 | 8.94E-24 | 0.5567 | 101.0554017 |
| rs2651472 | T | G | 0.0108 | -0.0001 | 0.0019 | 0.006539783 | 1.31E-08 | 0.9878 | 32.31024931 |
| rs2663126 | A | G | -0.0139 | -0.0069 | 0.0021 | 0.009931357 | 3.62E-11 | 0.4872 | 43.81179138 |
| rs2678898 | T | C | 0.0129 | -0.022 | 0.0019 | 0.011769062 | 1.13E-11 | 0.06158 | 46.09695291 |
| rs2721940 | A | C | 0.0165 | 0.0098 | 0.0019 | 0.009286687 | 3.81E-18 | 0.2913 | 75.41551247 |
| rs2788213 | A | G | 0.0123 | 0.0086 | 0.0021 | 0.009862212 | 4.71E-09 | 0.3832 | 34.30612245 |
| rs2807339 | T | C | -0.0162 | 0.021 | 0.0022 | 0.010552719 | 1.79E-13 | 4.66E-02 | 54.2231405 |
| rs2823990 | A | G | -0.0127 | -0.0068 | 0.002 | 0.009805429 | 2.15E-10 | 0.488 | 40.3225 |
| rs28468602 | T | G | -0.0112 | -0.001 | 0.0019 | 0.008876253 | 3.75E-09 | 0.9103 | 34.74792244 |
| rs28529055 | T | G | -0.0147 | 0.0113 | 0.0019 | 0.011483372 | 1.02E-14 | 0.3251 | 59.85872576 |
| rs2854152 | A | G | -0.0482 | -0.009 | 0.002 | 0.009730419 | 2.50E-128 | 0.355 | 580.81 |
| rs28592876 | A | G | 0.03 | -0.0187 | 0.0023 | 0.011119802 | 6.92E-39 | 0.09263 | 170.1323251 |
| rs28701981 | T | C | -0.0395 | -0.0145 | 0.002 | 0.009419087 | 8.02E-87 | 0.1237 | 390.0625 |
| rs28736838 | T | C | -0.0117 | 0.0041 | 0.002 | 0.009714851 | 4.92E-09 | 0.673 | 34.2225 |
| rs28757154 | A | G | -0.0193 | -0.0053 | 0.0027 | 0.01525088 | 8.80E-13 | 0.7282 | 51.09602195 |
| rs28787734 | T | C | 0.0385 | 0.0247 | 0.0041 | 0.028558595 | 5.99E-21 | 0.3871 | 88.17668055 |
| rs28817902 | A | G | -0.023 | -0.0102 | 0.0029 | 0.014926959 | 2.17E-15 | 4.94E-01 | 62.90130797 |
| rs2900208 | A | C | 0.0255 | 0.0107 | 0.002 | 0.009366487 | 3.12E-37 | 0.2533 | 162.5625 |
| rs291970 | T | C | 0.024 | -0.0327 | 0.0022 | 0.011124547 | 1.04E-27 | 0.003288 | 119.0082645 |
| rs2925155 | T | C | -0.015 | -0.0132 | 0.0022 | 0.010615578 | 9.22E-12 | 0.2137 | 46.48760331 |
| rs2965074 | T | G | 0.0125 | 0.0252 | 0.0019 | 0.00917886 | 4.74E-11 | 0.006043 | 43.28254848 |
| rs2974337 | T | C | 0.0117 | -0.004 | 0.0019 | 0.009101272 | 7.37E-10 | 0.6603 | 37.91966759 |
| rs2993531 | A | C | -0.0176 | -0.0146 | 0.0019 | 0.009083851 | 1.98E-20 | 0.108 | 85.80609418 |
| rs2994329 | T | C | 0.0155 | -0.0047 | 0.0023 | 0.011154639 | 1.59E-11 | 0.6735 | 45.41587902 |
| rs3003137 | T | G | -0.0114 | 0.0101 | 0.0019 | 0.009724814 | 1.97E-09 | 0.299 | 36 |
| rs301805 | T | G | 0.0146 | 0.0027 | 0.0019 | 0.009124444 | 1.54E-14 | 0.7673 | 59.04709141 |
| rs3103223 | T | C | -0.0126 | -0.004 | 0.0022 | 0.010384607 | 1.02E-08 | 0.7001 | 32.80165289 |
| rs3103268 | T | C | 0.0291 | 0.0151 | 0.0031 | 0.015462854 | 6.17E-21 | 3.29E-01 | 88.11758585 |
| rs3115084 | T | C | 0.024 | 0.0117 | 0.0031 | 0.017729205 | 9.79E-15 | 0.5093 | 59.93756504 |
| rs31196 | A | C | -0.0107 | 0.0037 | 0.0019 | 0.009078517 | 1.79E-08 | 0.6836 | 31.71468144 |
| rs332116 | T | C | -0.0206 | -0.0066 | 0.0021 | 0.01171879 | 1.02E-22 | 0.5733 | 96.22675737 |
| rs33973388 | T | G | 0.0249 | 0.0083 | 0.0019 | 0.009243041 | 3.07E-39 | 0.3692 | 171.7479224 |
| rs34287 | A | G | 0.0187 | 0.0228 | 0.002 | 0.009404048 | 8.76E-21 | 0.01533 | 87.4225 |
| rs34345560 | A | G | 0.0219 | 0.0053 | 0.0024 | 0.011321925 | 7.17E-20 | 0.6397 | 83.265625 |
| rs34522021 | T | C | 0.0126 | 0.01 | 0.0019 | 0.009109283 | 3.32E-11 | 0.2723 | 43.97783934 |
| rs34786000 | T | G | -0.015 | -0.0257 | 0.0019 | 0.009286397 | 2.91E-15 | 0.005649 | 62.32686981 |
| rs35073631 | T | C | -0.0112 | -0.0035 | 0.0019 | 0.011536672 | 3.75E-09 | 7.62E-01 | 34.74792244 |
| rs35268848 | A | C | 0.0737 | -0.0698 | 0.0101 | 0.048404711 | 2.94E-13 | 0.1493 | 53.24664249 |
| rs35453327 | A | C | 0.0283 | 0.0685 | 0.0036 | 0.017620774 | 3.81E-15 | 0.0001013 | 61.7970679 |
| rs35464459 | T | C | 0.0342 | -0.0111 | 0.0029 | 0.013785268 | 4.24E-32 | 0.4207 | 139.0772889 |
| rs35624335 | T | C | -0.0134 | 0.0003 | 0.0021 | 0.008608523 | 1.76E-10 | 0.9722 | 40.71655329 |
| rs35696197 | T | G | 0.0127 | -0.0016 | 0.0021 | 0.010779177 | 1.47E-09 | 0.882 | 36.57369615 |
| rs35732917 | T | C | -0.0204 | 0.004 | 0.0021 | 0.009870657 | 2.62E-22 | 0.6853 | 94.36734694 |
| rs35748083 | T | C | -0.0197 | 0.01 | 0.0019 | 0.00905621 | 3.45E-25 | 0.2695 | 107.5041551 |
| rs35756741 | T | C | -0.0378 | 0.0151 | 0.0033 | 0.015228145 | 2.23E-30 | 0.3214 | 131.2066116 |
| rs35816944 | A | G | -0.1088 | -0.1541 | 0.0117 | 0.063176566 | 1.42E-20 | 0.01472 | 86.47410329 |
| rs35874463 | A | G | -0.0263 | -0.0004 | 0.0041 | 0.020858449 | 1.41E-10 | 0.9847 | 41.14753123 |
| rs35892992 | T | C | -0.0182 | -0.0188 | 0.0023 | 0.01126466 | 2.51E-15 | 0.09513 | 62.61625709 |
| rs35963161 | A | G | -0.0157 | 0.0016 | 0.0019 | 0.00964912 | 1.42E-16 | 0.8683 | 68.27977839 |
| rs36048468 | T | C | 0.0254 | -0.0024 | 0.0023 | 0.011265777 | 2.36E-28 | 0.8313 | 121.9584121 |
| rs36226649 | T | C | -0.0485 | -0.0074 | 0.0038 | 0.018328385 | 2.63E-37 | 6.86E-01 | 162.8981994 |
| rs3625 | A | G | 0.0148 | 0.0055 | 0.0019 | 0.008983871 | 6.73E-15 | 0.5404 | 60.67590028 |
| rs372532055 | A | C | 0.0175 | 0.0057 | 0.002 | 0.015789506 | 2.13E-18 | 0.7181 | 76.5625 |
| rs3764002 | T | C | 0.028 | -0.0278 | 0.0021 | 0.0103467 | 1.48E-40 | 0.007213 | 177.7777778 |
| rs3769885 | A | G | -0.0111 | -0.0208 | 0.0019 | 0.008988217 | 5.15E-09 | 0.02066 | 34.13019391 |
| rs377599 | T | C | 0.0217 | -0.0061 | 0.0019 | 0.009199941 | 3.28E-30 | 0.5073 | 130.4404432 |
| rs3792819 | A | G | -0.021 | -0.0014 | 0.0034 | 0.015982974 | 6.56E-10 | 0.9302 | 38.14878893 |
| rs3814333 | T | C | 0.0182 | -0.0678 | 0.002 | 0.009710344 | 9.03E-20 | 2.91E-12 | 82.81 |
| rs3830008 | A | C | 0.0154 | -0.0011 | 0.0023 | 0.010710667 | 2.15E-11 | 0.9182 | 44.83175803 |
| rs3844 | A | C | 0.0132 | -0.0193 | 0.0022 | 0.010371393 | 1.97E-09 | 0.06276 | 36 |
| rs3853252 | A | G | 0.0241 | -0.0071 | 0.0019 | 0.009104728 | 7.23E-37 | 0.4355 | 160.8891967 |
| rs395980 | T | G | 0.0184 | 0.0088 | 0.0021 | 0.010260904 | 1.92E-18 | 0.3911 | 76.77097506 |
| rs4121583 | T | C | 0.0118 | -0.0005 | 0.002 | 0.011077969 | 3.64E-09 | 0.964 | 34.81 |
| rs41298373 | A | G | 0.0185 | 0.0016 | 0.0031 | 0.014832874 | 2.41E-09 | 0.9141 | 35.61394381 |
| rs4274112 | A | G | 0.0217 | 0.0185 | 0.002 | 0.009450472 | 1.99E-27 | 5.03E-02 | 117.7225 |
| rs4287835 | T | C | -0.0147 | -0.0089 | 0.0019 | 0.009035083 | 1.02E-14 | 0.3246 | 59.85872576 |
| rs4383083 | A | G | 0.0111 | -0.0503 | 0.002 | 0.009724987 | 2.86E-08 | 0.000000231 | 30.8025 |
| rs4444637 | A | G | -0.0179 | -0.005 | 0.0027 | 0.012854536 | 3.37E-11 | 0.6973 | 43.95198903 |
| rs447352 | T | C | -0.0181 | -0.0023 | 0.0029 | 0.02003404 | 4.34E-10 | 0.9086 | 38.9548157 |
| rs4504126 | A | C | -0.046 | -0.0158 | 0.0058 | 0.028275007 | 2.17E-15 | 0.5763 | 62.90130797 |
| rs45474992 | T | C | -0.0617 | -0.0324 | 0.0051 | 0.024643806 | 1.08E-33 | 0.1886 | 146.3625529 |
| rs4554207 | T | C | 0.0114 | 0.0029 | 0.0019 | 0.009364334 | 1.97E-09 | 0.7568 | 36 |
| rs4615815 | A | G | 0.0246 | 0.0119 | 0.0019 | 0.009416009 | 2.43E-38 | 0.2063 | 167.634349 |
| rs4622329 | A | G | 0.0149 | 0.0087 | 0.002 | 0.009573947 | 9.33E-14 | 0.3635 | 55.5025 |
| rs4652902 | A | C | 0.0129 | 0.0262 | 0.0022 | 0.010551972 | 4.53E-09 | 0.01303 | 34.3822314 |
| rs465983 | A | G | 0.0153 | 0.0051 | 0.0022 | 0.010488738 | 3.54E-12 | 0.6268 | 48.36570248 |
| rs4682483 | A | G | -0.0165 | 0.0085 | 0.0026 | 0.012147656 | 2.21E-10 | 0.4841 | 40.27366864 |
| rs4733775 | A | G | 0.0143 | 0.0149 | 0.002 | 0.009351846 | 8.68E-13 | 0.1111 | 51.1225 |
| rs4752829 | A | G | 0.0262 | 0.044 | 0.0021 | 0.010210244 | 1.01E-35 | 0.0000164 | 155.6553288 |
| rs4754296 | T | G | 0.0167 | -0.0076 | 0.0027 | 0.012826493 | 6.20E-10 | 0.5535 | 38.25651578 |
| rs4763327 | T | C | 0.0123 | 0.0084 | 0.0021 | 0.009786234 | 4.71E-09 | 0.3907 | 34.30612245 |
| rs4776624 | T | C | -0.015 | 0.0074 | 0.0019 | 0.009223891 | 2.91E-15 | 0.4224 | 62.32686981 |
| rs4799799 | A | C | -0.0113 | -0.0024 | 0.002 | 0.00998228 | 1.60E-08 | 0.81 | 31.9225 |
| rs4815952 | T | C | 0.0161 | 0.0086 | 0.0019 | 0.009424548 | 2.38E-17 | 0.3615 | 71.8033241 |
| rs4847378 | T | G | 0.0136 | -0.0256 | 0.0019 | 0.009243306 | 8.19E-13 | 0.005613 | 51.23545706 |
| rs4852257 | T | G | 0.0231 | 0.0081 | 0.0019 | 0.009209194 | 5.21E-34 | 0.3791 | 147.8144044 |
| rs4909912 | T | C | -0.0277 | -0.0091 | 0.0019 | 0.009041697 | 3.82E-48 | 0.3142 | 212.5457064 |
| rs4940874 | A | G | -0.0148 | -0.009 | 0.0024 | 0.011627125 | 6.97E-10 | 0.4389 | 38.02777778 |
| rs496783 | A | G | 0.0124 | 0.0033 | 0.0019 | 0.009247441 | 6.74E-11 | 0.7212 | 42.59279778 |
| rs4976262 | T | C | 0.0245 | -0.0054 | 0.002 | 0.009580604 | 1.68E-34 | 0.573 | 150.0625 |
| rs4979576 | T | C | 0.0317 | -0.001 | 0.0029 | 0.020089551 | 8.19E-28 | 0.9603 | 119.4875149 |
| rs4985445 | A | G | 0.0175 | 0.0254 | 0.0019 | 0.009081168 | 3.25E-20 | 0.005158 | 84.83379501 |
| rs501811 | T | C | 0.0199 | 0.011 | 0.003 | 0.013596591 | 3.28E-11 | 0.4185 | 44.00111111 |
| rs548466113 | T | C | -0.036 | 0.0044 | 0.0021 | 0.015055907 | 7.11E-66 | 7.70E-01 | 293.877551 |
| rs551473284 | T | C | 0.017 | -0.0271 | 0.002 | 0.013651498 | 1.90E-17 | 0.04713 | 72.25 |
| rs55633823 | T | C | 0.0146 | 0.01 | 0.0022 | 0.011441209 | 3.22E-11 | 0.3821 | 44.04132231 |
| rs55745410 | A | G | 0.0157 | -0.003 | 0.002 | 0.009337718 | 4.16E-15 | 0.748 | 61.6225 |
| rs55758152 | A | G | 0.0145 | 0.0153 | 0.002 | 0.010615418 | 4.17E-13 | 0.1495 | 52.5625 |
| rs56034226 | T | C | 0.0226 | -0.0128 | 0.0027 | 0.015128333 | 5.74E-17 | 0.3975 | 70.06310014 |
| rs56207248 | T | C | -0.0273 | 0.0271 | 0.0038 | 0.018478812 | 6.76E-13 | 0.1425 | 51.61288089 |
| rs56207600 | A | G | 0.0192 | 0.0081 | 0.003 | 0.014499216 | 1.55E-10 | 0.5764 | 40.96 |
| rs56208656 | A | G | -0.0284 | -0.037 | 0.0034 | 0.016322281 | 6.66E-17 | 0.0234 | 69.7716263 |
| rs56309431 | T | G | 0.0167 | 0.0052 | 0.0029 | 0.013720908 | 8.48E-09 | 0.7047 | 33.16171225 |
| rs56363908 | A | G | 0.0382 | 0.0114 | 0.0047 | 0.022990808 | 4.38E-16 | 0.62 | 66.05885016 |
| rs563738408 | A | G | -0.0587 | -0.108 | 0.0053 | 0.037099835 | 1.65E-28 | 0.003602 | 122.6660733 |
| rs57287582 | T | G | 0.0159 | -0.0012 | 0.0025 | 0.01798405 | 2.02E-10 | 0.9468 | 40.4496 |
| rs57307236 | A | G | -0.0155 | -0.0031 | 0.0019 | 0.01330756 | 3.41E-16 | 0.8158 | 66.55124654 |
| rs5742915 | T | C | -0.0248 | -0.0131 | 0.0019 | 0.00898624 | 6.14E-39 | 0.1449 | 170.3711911 |
| rs5763821 | A | C | -0.0191 | -0.005 | 0.002 | 0.012134338 | 1.30E-21 | 0.6803 | 91.2025 |
| rs57904377 | T | C | 0.0177 | -0.0068 | 0.0024 | 0.01178088 | 1.64E-13 | 0.5638 | 54.390625 |
| rs58738817 | A | G | -0.0257 | 0.0194 | 0.0022 | 0.010510691 | 1.58E-31 | 0.06493 | 136.464876 |
| rs59000092 | A | G | 0.0196 | -9.00E-04 | 0.0021 | 0.010230684 | 1.03E-20 | 0.9299 | 87.11111111 |
| rs591668 | A | G | -0.0174 | -0.0065 | 0.0019 | 0.009276645 | 5.29E-20 | 0.4835 | 83.86703601 |
| rs59725651 | A | G | 0.0167 | 0.0083 | 0.002 | 0.010588113 | 6.83E-17 | 0.4331 | 69.7225 |
| rs59753424 | A | G | 0.02 | -0.0068 | 0.0022 | 0.013271952 | 9.82E-20 | 0.6084 | 82.6446281 |
| rs59950280 | A | G | -0.0254 | -0.0194 | 0.002 | 0.009884731 | 5.91E-37 | 0.04969 | 161.29 |
| rs59951000 | T | C | -0.04 | -0.046 | 0.0047 | 0.021927401 | 1.73E-17 | 0.03592 | 72.43096424 |
| rs6000890 | T | C | 0.0135 | -0.0501 | 0.002 | 0.009329821 | 1.48E-11 | 7.88E-08 | 45.5625 |
| rs6028716 | A | G | -0.021 | -0.0042 | 0.0022 | 0.010409608 | 1.36E-21 | 0.6866 | 91.11570248 |
| rs60328144 | A | C | 0.0144 | 0.0128 | 0.0019 | 0.00923855 | 3.48E-14 | 0.1659 | 57.44044321 |
| rs603486 | T | C | -0.0127 | -0.015 | 0.002 | 0.009335371 | 2.15E-10 | 0.1081 | 40.3225 |
| rs60408354 | A | G | 0.0259 | 0.0175 | 0.0036 | 0.018005957 | 6.27E-13 | 0.3311 | 51.76003086 |
| rs6054390 | A | G | -0.0188 | -0.0091 | 0.002 | 0.009425277 | 5.46E-21 | 0.3343 | 88.36 |
| rs60804050 | A | G | -0.0217 | 0.006 | 0.0021 | 0.010357679 | 4.98E-25 | 0.5624 | 106.7777778 |
| rs6082354 | A | C | 0.024 | -0.0059 | 0.002 | 0.011171214 | 3.55E-33 | 0.5974 | 144 |
| rs612577 | T | C | 0.0152 | 0.0146 | 0.0026 | 0.012707882 | 5.03E-09 | 0.2506 | 34.17751479 |
| rs61729527 | T | C | -0.0346 | 0.0341 | 0.0043 | 0.020562187 | 8.52E-16 | 0.09724 | 64.74634938 |
| rs61732778 | A | G | 0.023 | 0.0111 | 0.0037 | 0.016499198 | 5.09E-10 | 0.5011 | 38.64134405 |
| rs61838776 | A | C | 0.0311 | -0.023 | 0.0025 | 0.012471811 | 1.59E-35 | 0.06516 | 154.7536 |
| rs61940146 | A | G | -0.0112 | -0.0145 | 0.002 | 0.009341218 | 2.14E-08 | 0.1206 | 31.36 |
| rs61944841 | A | G | 0.0253 | 0.0115 | 0.002 | 0.011040771 | 1.12E-36 | 0.2976 | 160.0225 |
| rs62033029 | A | G | -0.0141 | -0.0131 | 0.0023 | 0.011428771 | 8.76E-10 | 0.2517 | 37.58223062 |
| rs62048221 | T | C | -0.0242 | -0.0093 | 0.0022 | 0.010622459 | 3.82E-28 | 0.3813 | 121 |
| rs62103240 | A | G | 0.0212 | 0.003 | 0.0037 | 0.021711178 | 1.01E-08 | 0.8901 | 32.82980278 |
| rs62143873 | A | G | -0.0115 | 0.0249 | 0.0019 | 0.009257758 | 1.42E-09 | 0.007153 | 36.63434903 |
| rs62177315 | T | C | 0.0177 | -0.0142 | 0.0032 | 0.015665583 | 3.18E-08 | 0.3647 | 30.59472656 |
| rs62305043 | T | C | -0.0269 | 0.0024 | 0.004 | 0.028085094 | 1.76E-11 | 0.9319 | 45.225625 |
| rs62372061 | T | C | 0.0394 | 0.001 | 0.0032 | 0.016141183 | 7.76E-35 | 0.9506 | 151.5976563 |
| rs62449290 | A | G | 0.0213 | -0.0121 | 0.0026 | 0.01660919 | 2.56E-16 | 0.4663 | 67.11390533 |
| rs62466110 | T | C | 0.0371 | 0.0322 | 0.0041 | 0.020351188 | 1.45E-19 | 0.1136 | 81.88042832 |
| rs62501195 | A | C | 0.0198 | 0.0236 | 0.0025 | 0.012068311 | 2.38E-15 | 0.05052 | 62.7264 |
| rs631312 | A | G | -0.0126 | 0.004 | 0.0021 | 0.010139002 | 1.97E-09 | 0.6932 | 36 |
| rs6450136 | A | C | -0.0201 | -0.0011 | 0.002 | 0.009852114 | 9.19E-24 | 0.9111 | 101.0025 |
| rs6450961 | T | C | 0.0118 | -0.0194 | 0.002 | 0.009630771 | 3.64E-09 | 0.04397 | 34.81 |
| rs6461948 | T | C | 0.0112 | -0.0006 | 0.002 | 0.010469777 | 2.14E-08 | 0.9543 | 31.36 |
| rs6469845 | T | C | 0.0135 | -0.0031 | 0.0022 | 0.010655252 | 8.44E-10 | 0.7711 | 37.65495868 |
| rs6501381 | T | C | 0.0341 | -0.0014 | 0.003 | 0.014832206 | 6.13E-30 | 0.9248 | 129.2011111 |
| rs6505216 | T | G | -0.0498 | -0.0312 | 0.0023 | 0.013647859 | 5.80E-104 | 0.02225 | 468.8166352 |
| rs6543146 | T | G | -0.0154 | -0.0104 | 0.0019 | 0.009069396 | 5.26E-16 | 0.2515 | 65.69529086 |
| rs6544743 | T | G | 0.0217 | -0.0079 | 0.0024 | 0.011220752 | 1.54E-19 | 0.4814 | 81.75173611 |
| rs6567160 | T | C | -0.0488 | -0.0263 | 0.0022 | 0.01052128 | 5.15E-109 | 0.01243 | 492.0330579 |
| rs66613683 | T | C | -0.015 | 0.0157 | 0.0025 | 0.012174601 | 1.97E-09 | 0.1972 | 36 |
| rs6693481 | T | C | 0.0143 | 0.0166 | 0.002 | 0.009681602 | 8.68E-13 | 0.08642 | 51.1225 |
| rs670129 | T | C | -0.0118 | -0.0023 | 0.0019 | 0.00944468 | 5.28E-10 | 0.8076 | 38.57063712 |
| rs670318 | T | C | -0.0413 | 0.0324 | 0.0044 | 0.02099633 | 6.21E-21 | 0.1228 | 88.10382231 |
| rs6738207 | A | G | 0.0127 | 0.006 | 0.0019 | 0.009083319 | 2.32E-11 | 0.5089 | 44.67867036 |
| rs6739394 | T | C | -0.0144 | -0.0077 | 0.0019 | 0.009264492 | 3.48E-14 | 0.4059 | 57.44044321 |
| rs6789000 | T | G | 0.0121 | -0.0036 | 0.002 | 0.013739 | 1.45E-09 | 0.7933 | 36.6025 |
| rs68083605 | A | G | 0.0189 | 0.0194 | 0.0019 | 0.009159326 | 2.59E-23 | 0.03417 | 98.9501385 |
| rs680882 | T | G | -0.0133 | 0.0185 | 0.0022 | 0.010491695 | 1.49E-09 | 0.07785 | 36.54752066 |
| rs6844176 | T | C | -0.0129 | -0.0198 | 0.0019 | 0.009279796 | 1.13E-11 | 0.03287 | 46.09695291 |
| rs6852065 | T | C | 0.0131 | -0.0021 | 0.0019 | 0.009569405 | 5.40E-12 | 0.8263 | 47.53739612 |
| rs6854705 | T | C | 0.0173 | 0.0147 | 0.0024 | 0.011626414 | 5.66E-13 | 0.2061 | 51.96006944 |
| rs6899155 | T | C | 0.0282 | 0.0022 | 0.0019 | 0.009096722 | 7.83E-50 | 0.8089 | 220.2880886 |
| rs6910414 | A | G | 0.0142 | -0.0162 | 0.0024 | 0.011590382 | 3.29E-09 | 0.1622 | 35.00694444 |
| rs6923230 | T | C | 0.0127 | 0.0009 | 0.0019 | 0.009323569 | 2.32E-11 | 0.9231 | 44.67867036 |
| rs6931421 | T | G | 0.0279 | -0.0089 | 0.002 | 0.009540706 | 3.15E-44 | 0.3509 | 194.6025 |
| rs6943386 | T | C | -0.0106 | -0.0036 | 0.0019 | 0.009242416 | 2.42E-08 | 0.6969 | 31.12465374 |
| rs6960741 | T | C | -0.0185 | 0.0091 | 0.0029 | 0.014357448 | 1.78E-10 | 0.5262 | 40.69560048 |
| rs6962887 | T | G | 0.0127 | -0.0144 | 0.0021 | 0.010037614 | 1.47E-09 | 0.1514 | 36.57369615 |
| rs6963134 | A | G | -0.0137 | -0.0191 | 0.002 | 0.009525605 | 7.38E-12 | 0.04495 | 46.9225 |
| rs6975015 | A | G | 0.0211 | -0.0013 | 0.0029 | 0.014228139 | 3.44E-13 | 0.9272 | 52.93816885 |
| rs6977416 | A | G | 0.0457 | -0.0151 | 0.002 | 0.009569983 | 1.46E-115 | 0.1146 | 522.1225 |
| rs700677 | A | C | 0.0173 | -0.017 | 0.002 | 0.009713697 | 5.15E-18 | 0.0801 | 74.8225 |
| rs7014590 | T | C | 0.0228 | -0.0158 | 0.0022 | 0.010339972 | 3.63E-25 | 0.1265 | 107.4049587 |
| rs704832 | A | C | -0.012 | -0.005 | 0.0022 | 0.01056415 | 4.91E-08 | 0.636 | 29.75206612 |
| rs705953 | A | G | 0.019 | 0.0032 | 0.002 | 0.009562641 | 2.10E-21 | 0.7379 | 90.25 |
| rs7078507 | A | G | 0.02 | 0 | 0.0019 | NA | 6.53E-26 | 0.9962 | 110.8033241 |
| rs7095087 | A | G | 0.0117 | -0.0058 | 0.002 | 0.009367278 | 4.92E-09 | 0.5358 | 34.2225 |
| rs7095472 | A | G | -0.0267 | -0.0217 | 0.0019 | 0.009004788 | 7.42E-45 | 0.01596 | 197.4764543 |
| rs7107356 | A | G | -0.0133 | -0.0071 | 0.0019 | 0.00918053 | 2.56E-12 | 0.4393 | 49 |
| rs71384617 | T | C | 0.0133 | 0.0285 | 0.0021 | 0.013820284 | 2.40E-10 | 0.03919 | 40.11111111 |
| rs71519447 | A | G | -0.0716 | -0.0054 | 0.003 | 0.014712874 | 6.80E-126 | 0.7136 | 569.6177778 |
| rs7164187 | A | G | 0.0151 | -0.0142 | 0.0019 | 0.0091775 | 1.91E-15 | 0.1218 | 63.16066482 |
| rs7171129 | T | C | 0.012 | 0.0133 | 0.002 | 0.009801459 | 1.97E-09 | 0.1748 | 36 |
| rs7185244 | T | C | 0.0148 | -0.0007 | 0.0023 | 0.011230538 | 1.24E-10 | 0.9503 | 41.40642722 |
| rs718603 | T | C | 0.0131 | 0.0088 | 0.0021 | 0.009936077 | 4.43E-10 | 0.3758 | 38.9138322 |
| rs7225068 | A | G | -0.0141 | 0.0204 | 0.0019 | 0.009708031 | 1.16E-13 | 0.03561 | 55.07202216 |
| rs7228151 | T | C | 0.0185 | -0.0091 | 0.0023 | 0.010951277 | 8.73E-16 | 0.406 | 64.69754253 |
| rs7229520 | A | G | -0.0224 | 0.0019 | 0.002 | 0.009482989 | 4.08E-29 | 0.8412 | 125.44 |
| rs723149 | A | G | 0.0276 | 0.0002 | 0.0019 | 0.009612373 | 8.25E-48 | 0.9834 | 211.0138504 |
| rs7259285 | A | G | -0.0132 | 0.0258 | 0.0019 | 0.009925271 | 3.72E-12 | 0.009338 | 48.26592798 |
| rs72801818 | T | C | 0.0313 | -0.0175 | 0.0021 | 0.009948585 | 3.07E-50 | 0.07857 | 222.1519274 |
| rs72809820 | T | C | -0.0111 | 0.0146 | 0.002 | 0.009521897 | 2.86E-08 | 0.1252 | 30.8025 |
| rs72829852 | T | C | 0.0309 | 0.0112 | 0.0039 | 0.018366937 | 2.32E-15 | 0.542 | 62.77514793 |
| rs72841270 | T | G | -0.0294 | -0.0067 | 0.0028 | 0.01307313 | 8.64E-26 | 0.6083 | 110.25 |
| rs7286917 | A | G | -0.0171 | -0.0003 | 0.0023 | 0.012531021 | 1.05E-13 | 0.9809 | 55.27599244 |
| rs72908840 | T | G | 0.037 | 0.0526 | 0.0043 | 0.020582121 | 7.65E-18 | 0.0106 | 74.04002163 |
| rs7301341 | T | C | 0.0255 | -0.0461 | 0.002 | 0.009673944 | 3.12E-37 | 0.00000189 | 162.5625 |
| rs73040028 | T | C | 0.0168 | -0.0068 | 0.0022 | 0.010744221 | 2.23E-14 | 0.5268 | 58.31404959 |
| rs73125634 | T | G | -0.0195 | -0.0141 | 0.0021 | 0.010441647 | 1.61E-20 | 0.1769 | 86.2244898 |
| rs73158215 | A | G | 0.0163 | 0.0068 | 0.0023 | 0.011134813 | 1.37E-12 | 0.5414 | 50.22495274 |
| rs73186333 | A | C | -0.0373 | -0.0213 | 0.0063 | 0.027201461 | 3.21E-09 | 0.4336 | 35.05391786 |
| rs7320878 | A | G | -0.015 | -0.017 | 0.0019 | 0.010702716 | 2.91E-15 | 0.1122 | 62.32686981 |
| rs7328187 | T | G | -0.0116 | -0.0138 | 0.0019 | 0.009009681 | 1.03E-09 | 0.1256 | 37.27423823 |
| rs73382475 | T | C | 0.0254 | -0.05 | 0.0033 | 0.01595543 | 1.39E-14 | 0.001726 | 59.24334252 |
| rs73384223 | T | C | 0.0205 | -0.0027 | 0.0024 | 0.011603284 | 1.32E-17 | 0.816 | 72.96006944 |
| rs73490624 | T | C | -0.0184 | 0.0045 | 0.0024 | 0.011282294 | 1.77E-14 | 0.69 | 58.77777778 |
| rs7359097 | T | C | 0.0121 | 0.0024 | 0.0019 | 0.009297334 | 1.91E-10 | 0.7963 | 40.5567867 |
| rs7367519 | T | C | -0.0164 | -0.0036 | 0.002 | 0.009946515 | 2.40E-16 | 0.7174 | 67.24 |
| rs74048171 | A | C | -0.0121 | -0.005 | 0.0022 | 0.010446425 | 3.80E-08 | 0.6322 | 30.25 |
| rs7418410 | T | C | 0.0155 | -0.0066 | 0.0019 | 0.009174426 | 3.41E-16 | 0.4719 | 66.55124654 |
| rs7428883 | A | G | -0.0277 | 0.0152 | 0.0023 | 0.01108226 | 2.10E-33 | 0.1702 | 145.0453686 |
| rs7448554 | A | C | -0.0132 | -0.0117 | 0.002 | 0.011791978 | 4.11E-11 | 0.3211 | 43.56 |
| rs7485647 | A | G | -0.0261 | -0.0154 | 0.0026 | 0.012276876 | 1.03E-23 | 0.2097 | 100.7707101 |
| rs75022676 | A | G | -0.0163 | 0.0082 | 0.0023 | 0.011311565 | 1.37E-12 | 0.4685 | 50.22495274 |
| rs75100513 | T | C | -0.0199 | 0.0112 | 0.0034 | 0.015442997 | 4.83E-09 | 0.4683 | 34.25692042 |
| rs75172776 | A | G | -0.0232 | -0.064 | 0.0038 | 0.019797318 | 1.03E-09 | 0.001226 | 37.27423823 |
| rs7543136 | T | C | -0.021 | 0.0107 | 0.0021 | 0.011558775 | 1.52E-23 | 0.3546 | 100 |
| rs75702986 | A | G | -0.0163 | 0.0101 | 0.0025 | 0.011714776 | 7.03E-11 | 0.3886 | 42.5104 |
| rs757042 | T | C | 0.014 | -0.011 | 0.0021 | 0.010011854 | 2.62E-11 | 0.2719 | 44.44444444 |
| rs7574162 | T | C | 0.0153 | -0.0001 | 0.0022 | 0.0115634 | 3.54E-12 | 0.9931 | 48.36570248 |
| rs7582516 | T | C | 0.0254 | 0.0066 | 0.002 | 0.009798885 | 5.91E-37 | 0.5006 | 161.29 |
| rs7598430 | T | C | -0.016 | -0.0077 | 0.0019 | 0.00899347 | 3.73E-17 | 0.3919 | 70.91412742 |
| rs7633464 | A | G | 0.0175 | 0.0095 | 0.0019 | 0.00892804 | 3.25E-20 | 0.2873 | 84.83379501 |
| rs76364830 | A | G | -0.0471 | -0.0215 | 0.0039 | 0.020138588 | 1.40E-33 | 0.2857 | 145.852071 |
| rs7646501 | A | G | 0.017 | 0.0128 | 0.0021 | 0.0102288 | 5.72E-16 | 0.2108 | 65.53287982 |
| rs76488803 | A | G | -0.024 | -0.0216 | 0.0034 | 0.016787172 | 1.68E-12 | 0.1982 | 49.82698962 |
| rs76520574 | T | C | -0.0449 | -0.0077 | 0.0049 | 0.025759278 | 5.04E-20 | 0.765 | 83.96543107 |
| rs7666804 | T | C | 0.0167 | -0.0223 | 0.0019 | 0.012035338 | 1.50E-18 | 0.0639 | 77.25484765 |
| rs76693355 | T | C | 0.0268 | -0.0182 | 0.003 | 0.013833731 | 4.13E-19 | 0.1883 | 79.80444444 |
| rs7692387 | A | G | 0.0169 | -0.0088 | 0.0024 | 0.011458655 | 1.90E-12 | 0.4425 | 49.58506944 |
| rs7701233 | T | C | 0.0179 | 0.0081 | 0.0019 | 0.00980621 | 4.47E-21 | 0.4088 | 88.75623269 |
| rs7730092 | T | G | 0.0131 | 0.0127 | 0.0024 | 0.011405627 | 4.81E-08 | 0.2655 | 29.79340278 |
| rs7731023 | A | G | -0.0166 | 0.0054 | 0.0019 | 0.009286245 | 2.40E-18 | 0.5609 | 76.33240997 |
| rs77542162 | A | G | -0.0576 | -0.0335 | 0.0064 | 0.033059144 | 2.26E-19 | 0.3109 | 81 |
| rs7761910 | T | C | -0.016 | -0.0267 | 0.002 | 0.009839391 | 1.24E-15 | 0.006656 | 64 |
| rs7768382 | T | C | 0.0201 | -0.0072 | 0.0019 | 0.009123259 | 3.73E-26 | 0.43 | 111.9141274 |
| rs7781964 | A | G | 0.0261 | -0.0013 | 0.0024 | 0.011249412 | 1.52E-27 | 0.908 | 118.265625 |
| rs778384 | A | G | 0.0283 | -0.0127 | 0.0022 | 0.010974984 | 7.21E-38 | 0.2472 | 165.4731405 |
| rs78030362 | A | G | -0.0216 | 0.0077 | 0.0036 | 0.019941443 | 1.97E-09 | 0.6994 | 36 |
| rs7816345 | T | C | 0.0255 | 0.0105 | 0.0025 | 0.011975484 | 1.98E-24 | 0.3806 | 104.04 |
| rs78444298 | A | G | -0.0465 | 0.0227 | 0.0068 | 0.034700272 | 8.02E-12 | 0.513 | 46.76146194 |
| rs78457529 | T | C | -0.0904 | -0.097 | 0.0088 | 0.038787006 | 9.35E-25 | 0.01239 | 105.5289256 |
| rs7858712 | A | G | -0.0347 | -0.015 | 0.0034 | 0.016063161 | 1.87E-24 | 0.3504 | 104.1600346 |
| rs7893378 | A | G | 0.0175 | -0.0203 | 0.0031 | 0.015304332 | 1.65E-08 | 0.1847 | 31.86784599 |
| rs7910211 | T | C | -0.0175 | -0.0067 | 0.0026 | 0.012304951 | 1.69E-11 | 0.5861 | 45.30325444 |
| rs79680939 | A | G | 0.029 | 0.0184 | 0.0046 | 0.025144008 | 2.89E-10 | 0.4643 | 39.74480151 |
| rs798528 | A | C | 0.0357 | -0.0044 | 0.002 | 0.009762418 | 2.89E-71 | 0.6522 | 318.6225 |
| rs8017006 | A | G | -0.0122 | -0.0092 | 0.002 | 0.012800145 | 1.06E-09 | 0.4723 | 37.21 |
| rs8018486 | A | G | 0.0138 | 0.0025 | 0.0024 | 0.011260357 | 8.92E-09 | 0.8243 | 33.0625 |
| rs8019890 | A | C | 0.025 | 0.009 | 0.0019 | 0.009339076 | 1.53E-39 | 0.3352 | 173.1301939 |
| rs80280630 | T | C | -0.0168 | 0.0427 | 0.003 | 0.01476739 | 2.14E-08 | 0.003834 | 31.36 |
| rs8042545 | A | G | 0.0287 | 0.0168 | 0.0022 | 0.010366428 | 6.75E-39 | 0.1051 | 170.1838843 |
| rs8054549 | A | C | -0.0251 | -0.0124 | 0.0019 | 0.009110658 | 7.63E-40 | 0.1735 | 174.5180055 |
| rs8077636 | T | G | -0.0192 | 0.0063 | 0.0019 | 0.010157364 | 5.24E-24 | 0.5351 | 102.1163435 |
| rs8084413 | A | G | -0.0127 | 0.0117 | 0.0019 | 0.009341569 | 2.32E-11 | 0.2104 | 44.67867036 |
| rs8099461 | A | G | 0.0246 | -0.0116 | 0.0041 | 0.020373128 | 1.97E-09 | 0.5691 | 36 |
| rs8107967 | A | G | 0.0174 | 0.0107 | 0.0019 | 0.007629853 | 5.29E-20 | 0.1608 | 83.86703601 |
| rs8176632 | T | C | -0.0158 | -0.0029 | 0.0028 | 0.01286102 | 1.67E-08 | 0.8216 | 31.84183673 |
| rs8180765 | A | G | 0.0151 | -0.0101 | 0.0023 | 0.01104764 | 5.20E-11 | 0.3606 | 43.1020794 |
| rs876122 | A | G | -0.0162 | -0.0448 | 0.0029 | 0.014396442 | 2.32E-08 | 0.001859 | 31.20570749 |
| rs8904 | A | G | -0.0157 | -0.0001 | 0.002 | 0.016283256 | 4.16E-15 | 0.9951 | 61.6225 |
| rs894736 | A | G | 0.0174 | 0.0038 | 0.002 | 0.009373977 | 3.32E-18 | 0.6852 | 75.69 |
| rs921142 | T | C | 0.0111 | 0.0004 | 0.0019 | 0.009552712 | 5.15E-09 | 0.9666 | 34.13019391 |
| rs9344126 | T | C | 0.0185 | -0.0059 | 0.0019 | 0.008965757 | 2.10E-22 | 0.5105 | 94.80609418 |
| rs9376478 | A | G | -0.0186 | 0.0091 | 0.0022 | 0.010881583 | 2.80E-17 | 0.403 | 71.47933884 |
| rs945508 | T | C | 0.0128 | -0.0088 | 0.0019 | 0.009020785 | 1.62E-11 | 0.3293 | 45.38504155 |
| rs9479012 | A | G | -0.0263 | -0.0089 | 0.0032 | 0.015906204 | 2.06E-16 | 0.5758 | 67.54785156 |
| rs9492799 | A | G | 0.0182 | -0.0095 | 0.0026 | 0.012134746 | 2.56E-12 | 0.4337 | 49 |
| rs9579402 | T | C | -0.0203 | -0.0036 | 0.0035 | 0.016252526 | 6.63E-09 | 0.8247 | 33.64 |
| rs9590328 | A | G | -0.0153 | -0.0101 | 0.0027 | 0.013059627 | 1.46E-08 | 0.4393 | 32.11111111 |
| rs9610447 | T | C | 0.0152 | -0.0138 | 0.0022 | 0.010446599 | 4.88E-12 | 0.1865 | 47.73553719 |
| rs963317 | A | G | 0.0136 | 0.02 | 0.002 | 0.009462034 | 1.05E-11 | 0.03454 | 46.24 |
| rs9636364 | A | G | 0.011 | 0.0113 | 0.0019 | 0.009061798 | 7.06E-09 | 0.2124 | 33.51800554 |
| rs9659061 | A | G | -0.0198 | 0 | 0.0019 | NA | 1.99E-25 | 0.9983 | 108.598338 |
| rs9696116 | T | C | 0.0188 | -0.0019 | 0.002 | 0.009519439 | 5.46E-21 | 0.8418 | 88.36 |
| rs9784904 | T | C | 0.0217 | -0.0194 | 0.0026 | 0.012528187 | 7.05E-17 | 0.1215 | 69.65828402 |
| rs9807032 | T | C | 0.024 | 0.027 | 0.0024 | 0.011460795 | 1.52E-23 | 0.01848 | 100 |
| rs9809116 | A | G | 0.016 | 0.0071 | 0.0019 | 0.009202638 | 3.73E-17 | 0.4404 | 70.91412742 |
| rs9817452 | T | G | 0.0165 | -0.0038 | 0.0019 | 0.009355134 | 3.81E-18 | 0.6846 | 75.41551247 |
| rs9828525 | T | C | 0.0121 | 0.0071 | 0.0019 | 0.009061241 | 1.91E-10 | 0.4333 | 40.5567867 |
| rs9832919 | A | G | 0.0179 | -0.0055 | 0.002 | 0.00940535 | 3.55E-19 | 0.5587 | 80.1025 |
| rs9838614 | T | G | 0.0185 | 0.0033 | 0.0019 | 0.009317156 | 2.10E-22 | 0.7232 | 94.80609418 |
| rs9853018 | T | C | 0.0469 | 0.0021 | 0.0019 | 0.009161611 | 1.58E-134 | 0.8187 | 609.3102493 |
| rs9894577 | A | G | -0.031 | 0.0191 | 0.002 | 0.009681311 | 3.47E-54 | 0.04851 | 240.25 |
| rs9905385 | A | G | 0.0339 | -0.02 | 0.002 | 0.009599451 | 1.92E-64 | 0.03721 | 287.3025 |
| rs9910161 | A | G | -0.0162 | 0.0087 | 0.0021 | 0.010016835 | 1.22E-14 | 0.3851 | 59.51020408 |
| rs9957318 | A | G | -0.0187 | -0.0078 | 0.002 | 0.00943496 | 8.76E-21 | 0.4084 | 87.4225 |

**Supplementary Table 5** Genome-wide significant SNPs for the Association between Low hand grip as exposure and Hip osteoarthritis as outcome

| SNP | alt | ref | beta. exposure | beta. outcome | se. exposure | se. outcome | pval. exposure | pval. outcome | F |
| --- | --- | --- | --- | --- | --- | --- | --- | --- | --- |
| rs10952289 | T | C | 0.0435 | 0.0145 | 0.0078 | 0.009476706 | 2.45E-08 | 0.126 | 31.10207101 |
| rs11236213 | A | G | -0.0504 | -0.0098 | 0.008 | 0.009846473 | 2.98E-10 | 0.3196 | 39.69 |
| rs12140813 | T | C | 0.0511 | -0.0017 | 0.0094 | 0.011651783 | 5.44E-08 | 0.884 | 29.55194658 |
| rs143384 | A | G | 0.0545 | 0.024 | 0.0075 | 0.009198698 | 3.68E-13 | 0.009079 | 52.80444444 |
| rs143459567 | T | C | 0.1185 | -0.001 | 0.0189 | 0.024695559 | 3.61E-10 | 0.9677 | 39.31090955 |
| rs2899611 | T | G | -0.0431 | -0.0148 | 0.0074 | 0.009016311 | 5.73E-09 | 0.1007 | 33.92275383 |
| rs3118903 | A | G | 0.0575 | -0.0266 | 0.0088 | 0.010875441 | 6.40E-11 | 0.01445 | 42.69434401 |
| rs62102286 | T | G | 0.0487 | -0.0079 | 0.0074 | 0.009238768 | 4.67E-11 | 0.3925 | 43.3106282 |
| rs7624084 | T | C | 0.0428 | -0.0017 | 0.0074 | 0.008989076 | 7.30E-09 | 0.85 | 33.45215486 |
| rs79723785 | T | C | -0.1674 | -0.1971 | 0.0293 | 0.032648078 | 1.11E-08 | 1.57E-09 | 32.64191779 |

**Supplementary Table 6** Genome-wide significant SNPs for the Association between Usual walking pace as exposure and Hip osteoarthritis as outcome

| SNP | alt | ref | beta. exposure | beta. outcome | se. exposure | se. outcome | pval. exposure | pval. outcome | F |
| --- | --- | --- | --- | --- | --- | --- | --- | --- | --- |
| rs10750025 | T | C | -0.0083554 | 0.0225 | 0.00136528 | 0.009830407 | 9.36E-10 | 0.02209 | 37.45338156 |
| rs10862220 | G | T | 0.00840767 | -0.0069 | 0.00135051 | 0.009539705 | 4.80E-10 | 0.4695 | 38.75749557 |
| rs11077815 | C | T | -0.00713336 | -0.0084 | 0.00130691 | 0.009389653 | 4.81E-08 | 0.371 | 29.79181224 |
| rs11152989 | T | C | -0.00751226 | 0.0067 | 0.00136699 | 0.009681249 | 3.90E-08 | 0.4889 | 30.20025054 |
| rs11682482 | G | T | 0.00801894 | -0.001 | 0.00135595 | 0.009248928 | 3.34E-09 | 0.9139 | 34.97410239 |
| rs11848096 | C | T | -0.00750831 | 0.0088 | 0.00130696 | 0.00932262 | 9.20E-09 | 0.3452 | 33.00348477 |
| rs144333966 | G | A | 0.0304614 | -0.0673 | 0.00544615 | 0.034383771 | 2.23E-08 | 0.05031 | 31.28387355 |
| rs2037735 | T | C | -0.0112677 | 0.0402 | 0.00194308 | 0.013833524 | 6.68E-09 | 0.003661 | 33.62708237 |
| rs2170670 | A | G | -0.00708769 | 0.0003 | 0.0012972 | 0.008577655 | 4.66E-08 | 0.9721 | 29.85352018 |
| rs2602731 | G | A | -0.00766675 | 0.0151 | 0.00136907 | 0.009764405 | 2.14E-08 | 0.122 | 31.35965641 |
| rs2645979 | A | G | 0.00861798 | 0.0119 | 0.00132024 | 0.009304205 | 6.68E-11 | 0.2009 | 42.6093776 |
| rs4109292 | A | G | 0.00735336 | -0.002 | 0.00126787 | 0.009055434 | 6.64E-09 | 0.8252 | 33.63738338 |
| rs4839898 | A | G | 0.0130564 | 0.0065 | 0.00206037 | 0.01425917 | 2.34E-10 | 0.6485 | 40.15655595 |
| rs6763292 | G | A | 0.00958833 | -0.0122 | 0.00153076 | 0.010833324 | 3.76E-10 | 0.2601 | 39.23482636 |
| rs7789719 | C | T | 0.00854199 | -0.0045 | 0.00153719 | 0.010816952 | 2.75E-08 | 0.6774 | 30.87898498 |
| rs8011870 | A | G | -0.00782739 | 0.009 | 0.00140635 | 0.010060342 | 2.61E-08 | 0.371 | 30.97755318 |
