# Supplementary material for: Causal relationship between sarcopenia with osteoarthritis and the mediating role of obesity: a univariate, multivariate, two-step Mendelian randomization study
Source: BMC Geriatr. 2024 May 29;24:469. doi: 10.1186/s12877-024-05098-8 (PMC11138082; doi:10.1186/s12877-024-05098-8)
Supplement: Supplementary file 2 — Supplementary Material 2. [file 12877_2024_5098_MOESM2_ESM.docx]

**Supplementary Material 2**

1. **Supplementary Figure 1** Scatter plots in the univariate Mendelian Randomization study of Sarcopenia phenotypes and Knee osteoarthritis 2
2. **Supplementary Figure 2** Scatter plots in the univariate Mendelian Randomization study of Sarcopenia phenotypes and Hip osteoarthritis 3
3. **Supplementary Figure 3** Funnel plots in the univariate Mendelian Randomization study of Sarcopenia phenotypes and Knee osteoarthritis 4
4. **Supplementary Figure 4** Funnel plots in the univariate Mendelian Randomization study of Sarcopenia phenotypes and Hip osteoarthritis 5
5. **Supplementary Figure 5** Leave-one-out sensitivity analysis in the univariate Mendelian Randomization study of Sarcopenia phenotypes and Knee osteoarthritis 6
6. **Supplementary Figure 6** Leave-one-out sensitivity analysis in the univariate Mendelian Randomization study of Sarcopenia phenotypes and Hip osteoarthritis 7

**Supplementary Figure 1** Scatter plots in the univariate Mendelian Randomization study of Sarcopenia phenotypes and Knee osteoarthritis

| A | B | C |
| --- | --- | --- |
| 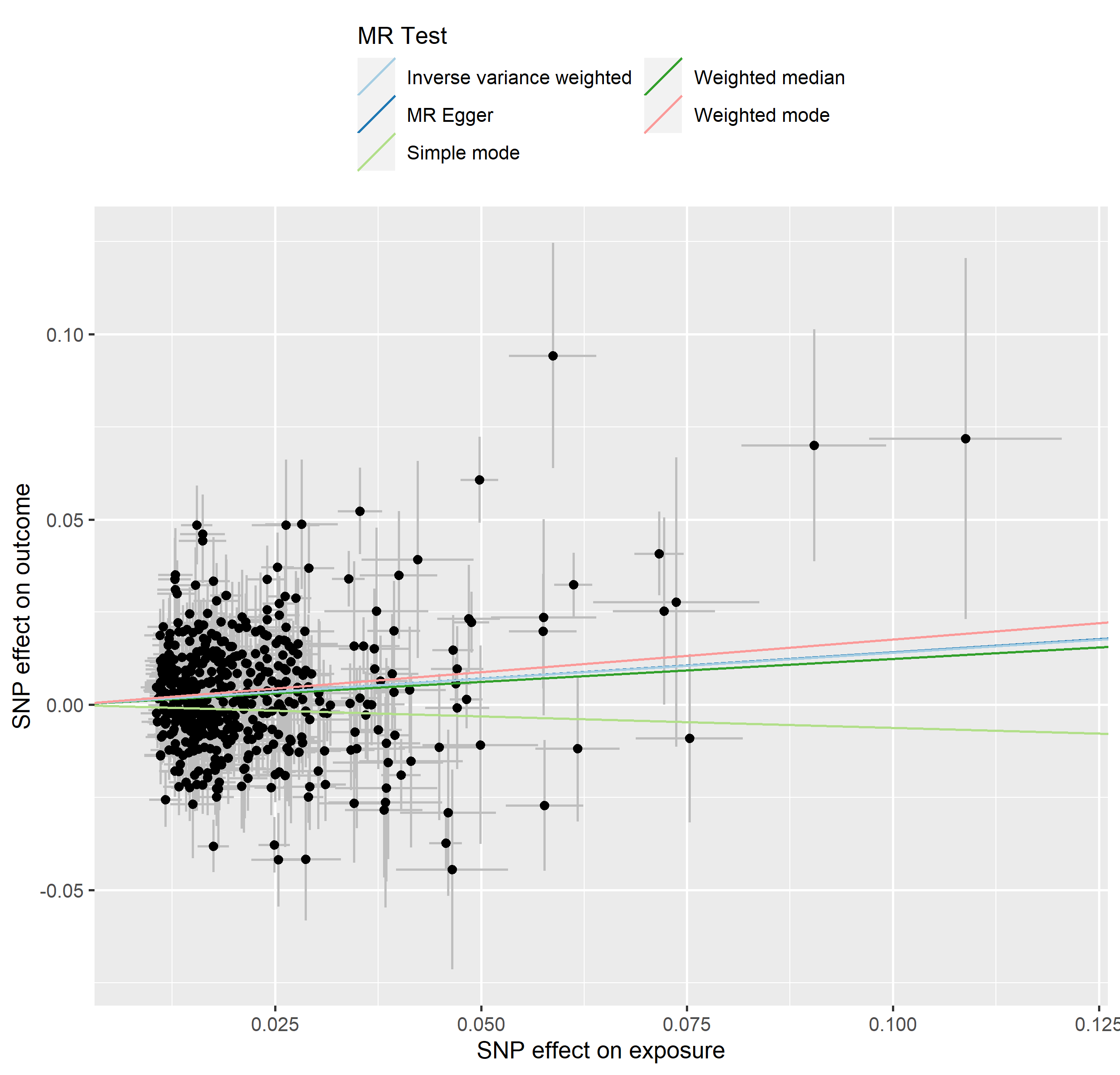 | 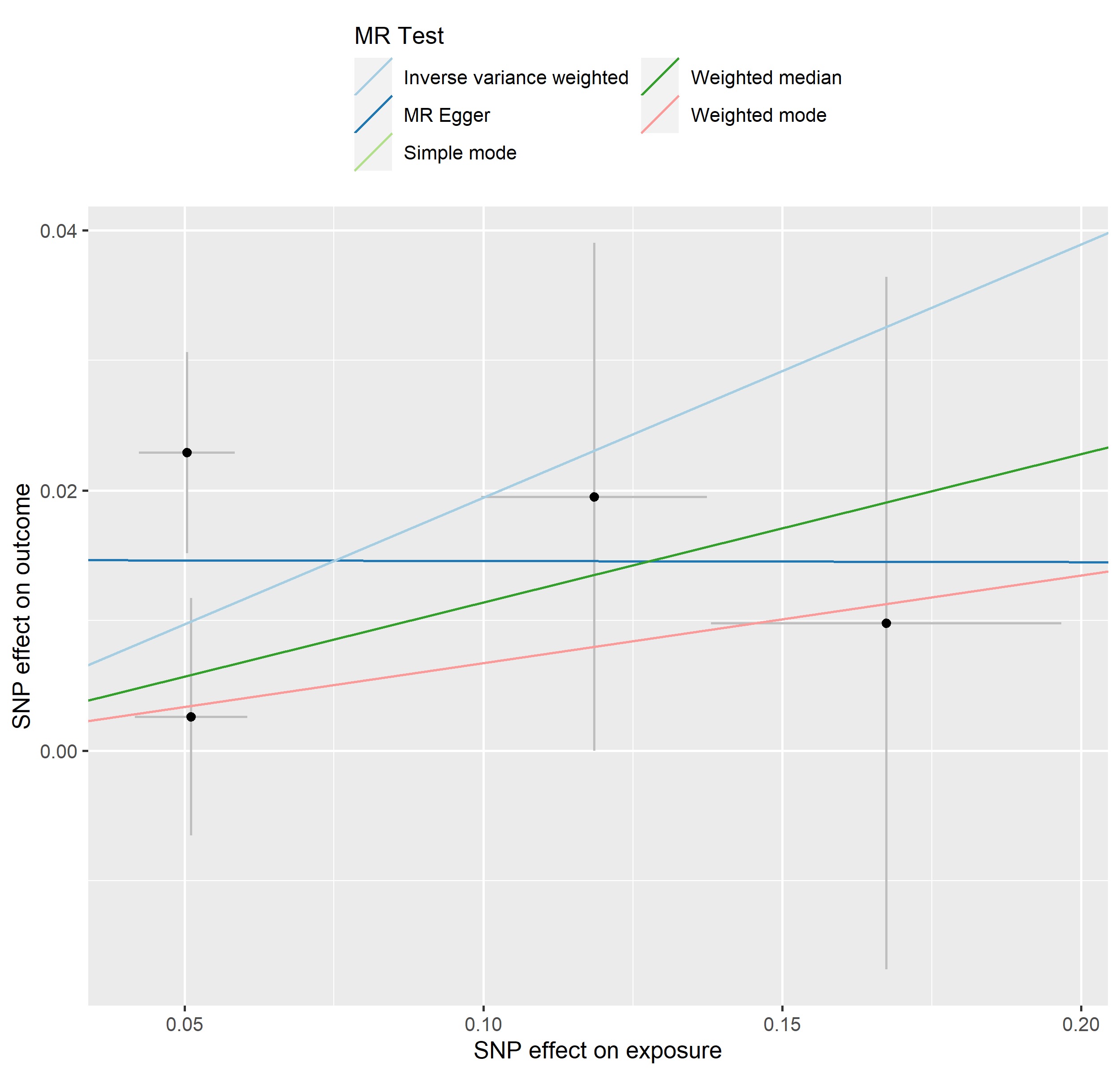 | 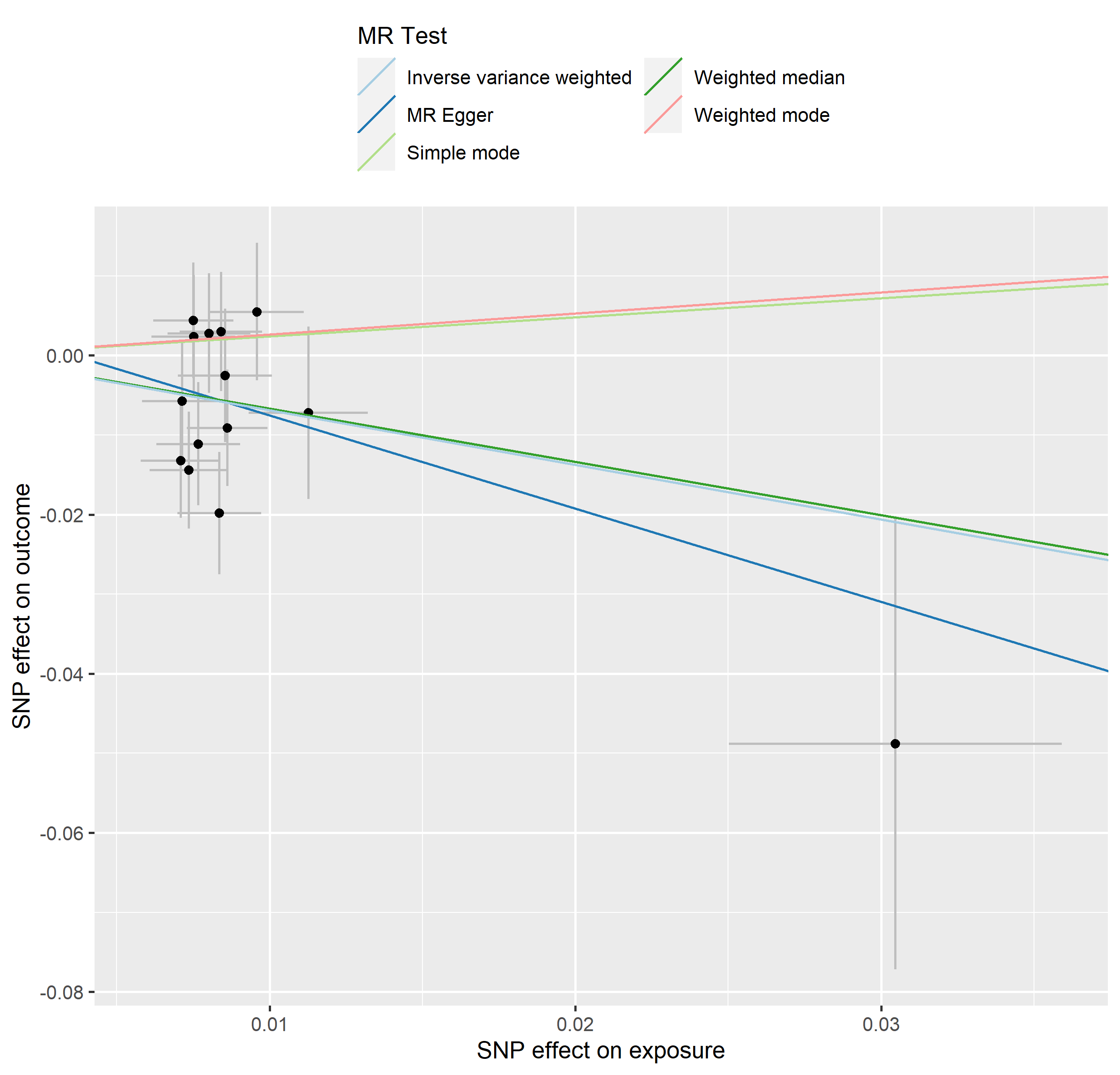 |

Figure note: A: Scatter plot of the causal effects of SNP associated with Appendicular lean mass on Knee osteoarthritis. B: Scatter plot of the causal effects of SNP associated with Low hand grip on Knee osteoarthritis. C: Scatter plot of the causal effects of SNP associated with Usual walking pace on Knee osteoarthritis.

**Supplementary Figure 2** Scatter plots in the univariate Mendelian Randomization study of Sarcopenia phenotypes and Hip osteoarthritis

| A | B | C |
| --- | --- | --- |
| 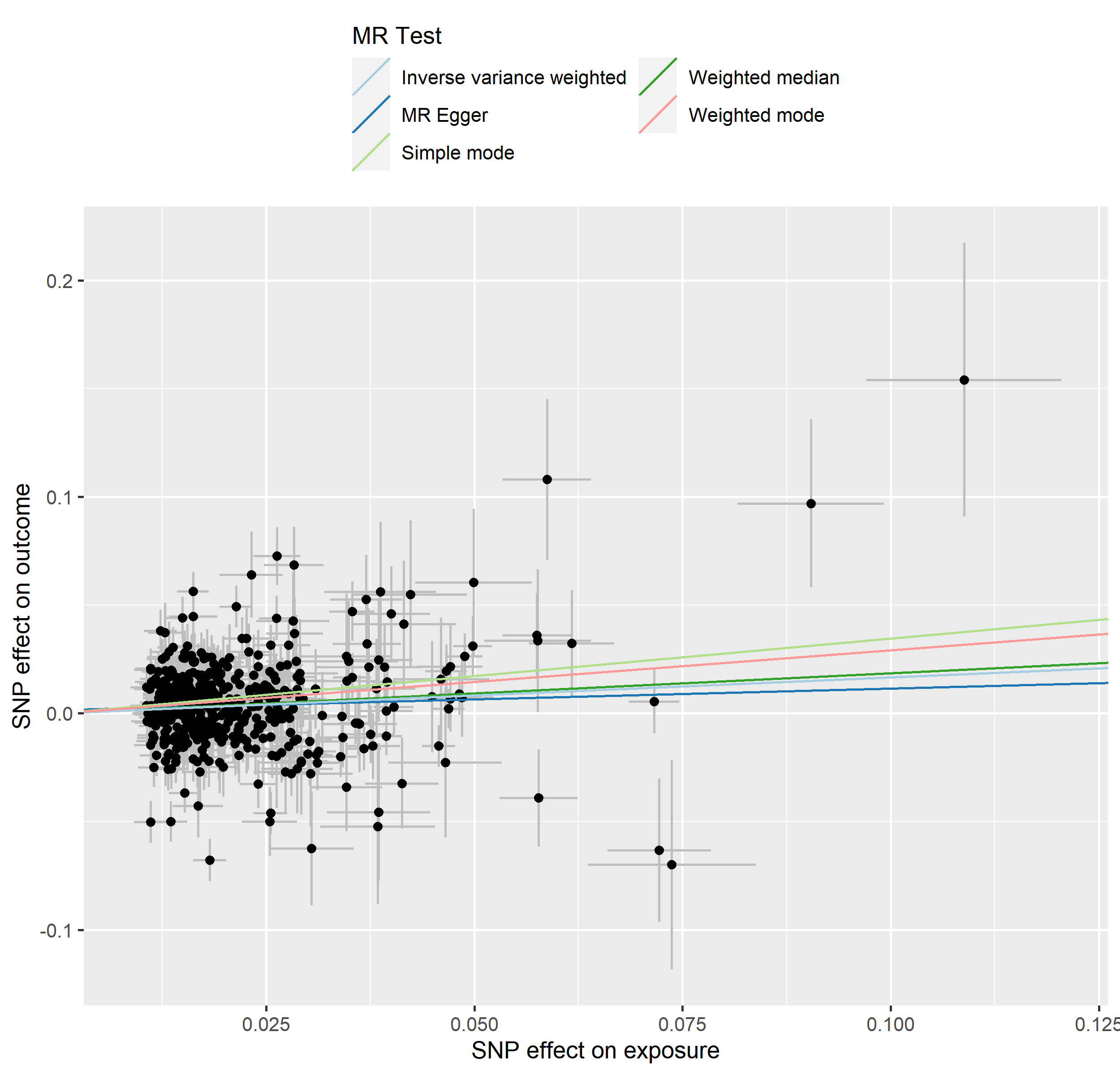 | 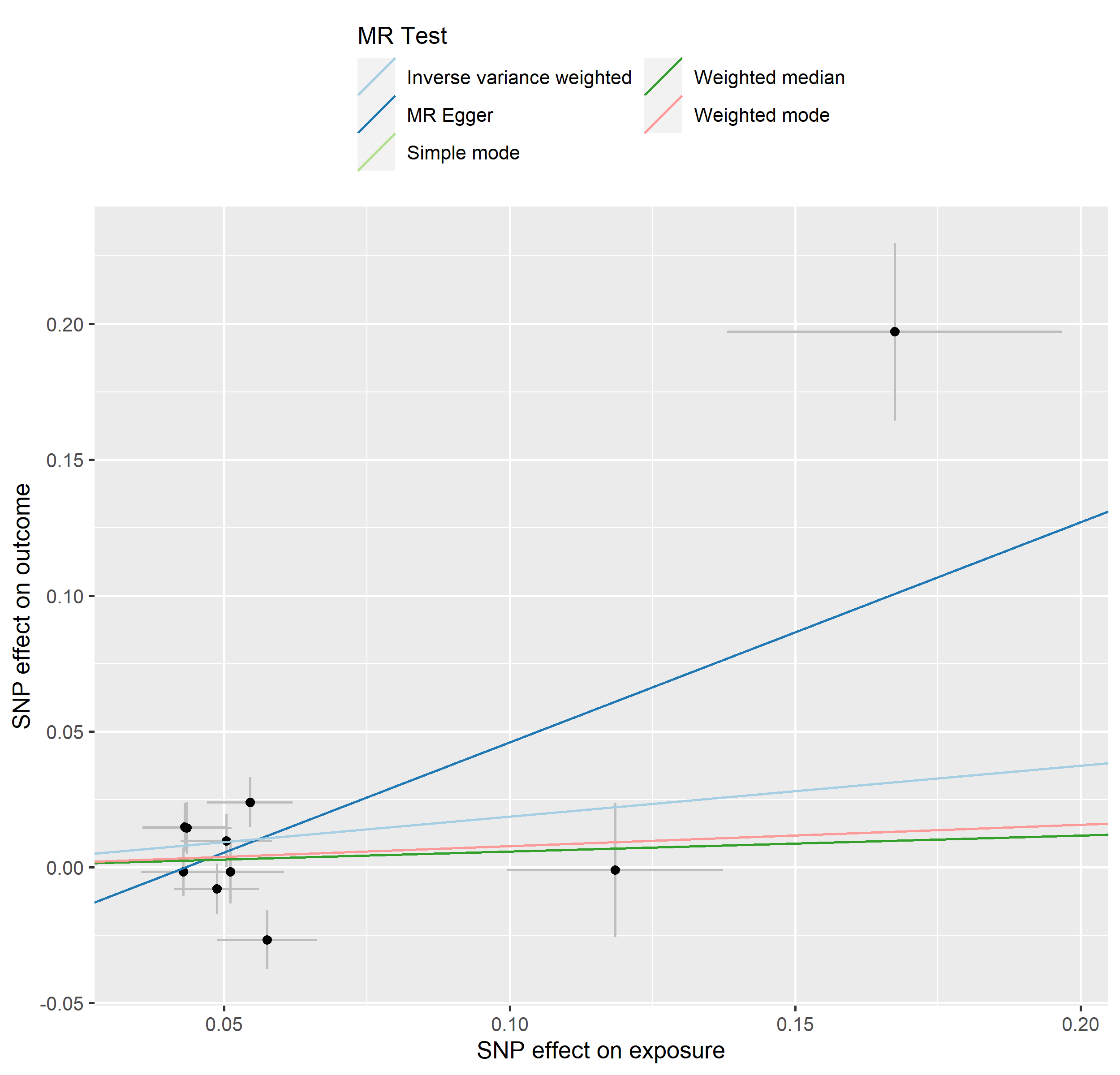 | 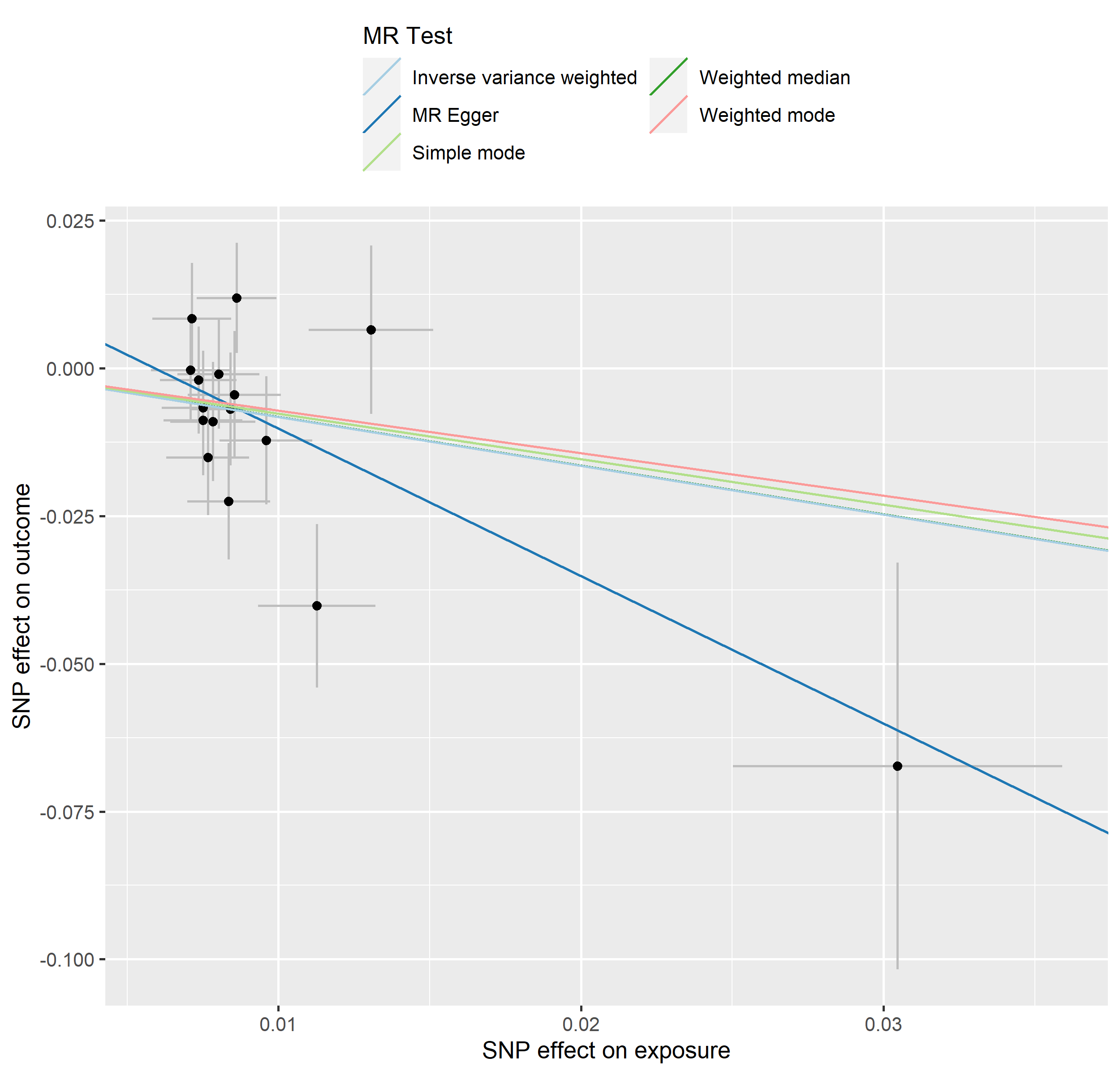 |

Figure note: A: Scatter plot of the causal effects of SNP associated with Appendicular lean mass on Hip osteoarthritis. B: Scatter plot of the causal effects of SNP associated with Low hand grip on Hip osteoarthritis. C: Scatter plot of the causal effects of SNP associated with Usual walking pace on Hip osteoarthritis.

**Supplementary Figure 3** Funnel plots in the univariate Mendelian Randomization study of Sarcopenia phenotypes and Knee osteoarthritis

| A | B | C |
| --- | --- | --- |
| 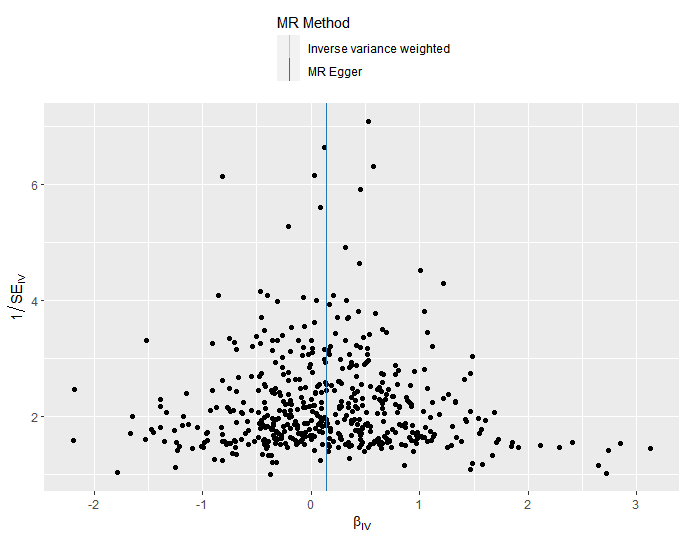 | 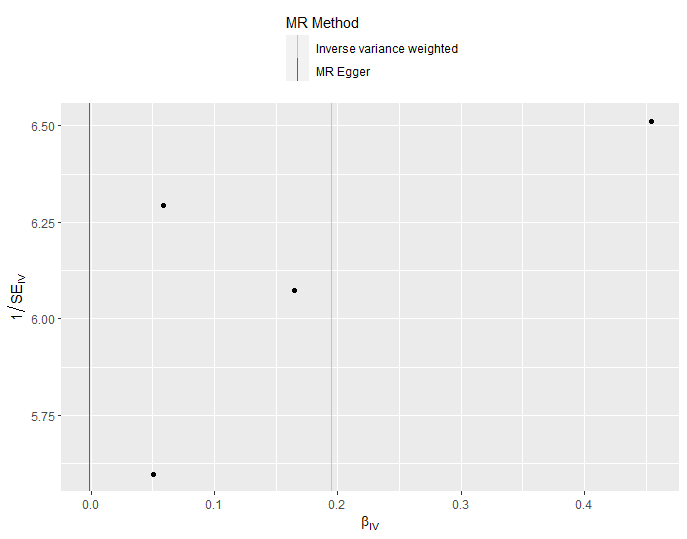 | 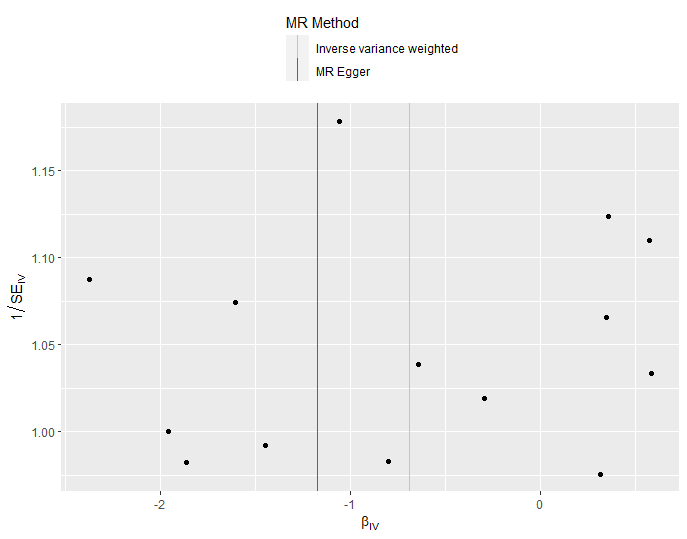 |

Figure note: A: Funnel plot of the causal effects of SNP associated with Appendicular lean mass on Knee osteoarthritis. B: Funnel plot of the causal effects of SNP associated with Low hand grip on Knee osteoarthritis. C: Funnel plot of the causal effects of SNP associated with Usual walking pace on Knee osteoarthritis.

**Supplementary Figure 4** Funnel plots in the univariate Mendelian Randomization study of Sarcopenia phenotypes and Hip osteoarthritis

| A | B | C |
| --- | --- | --- |
| 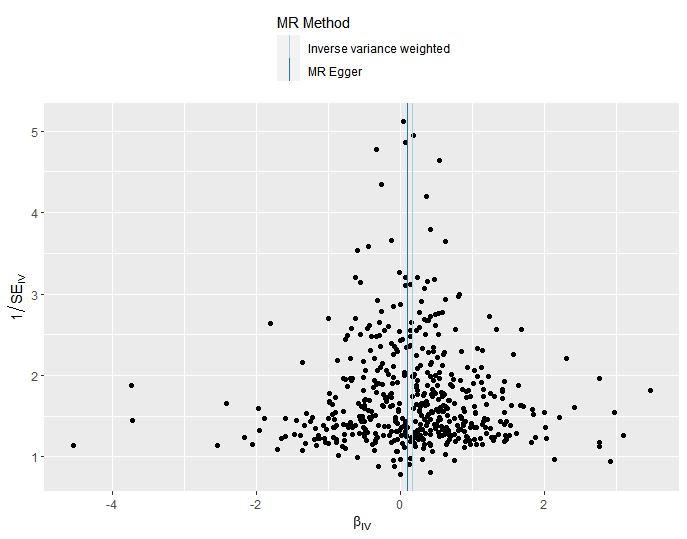 | 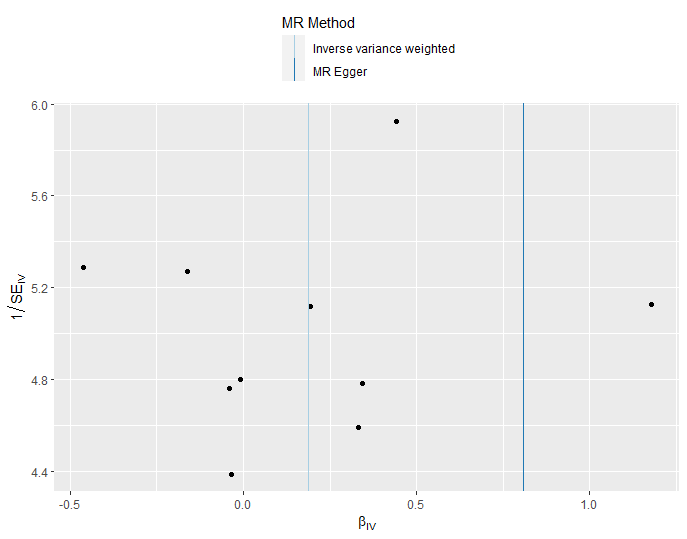 | 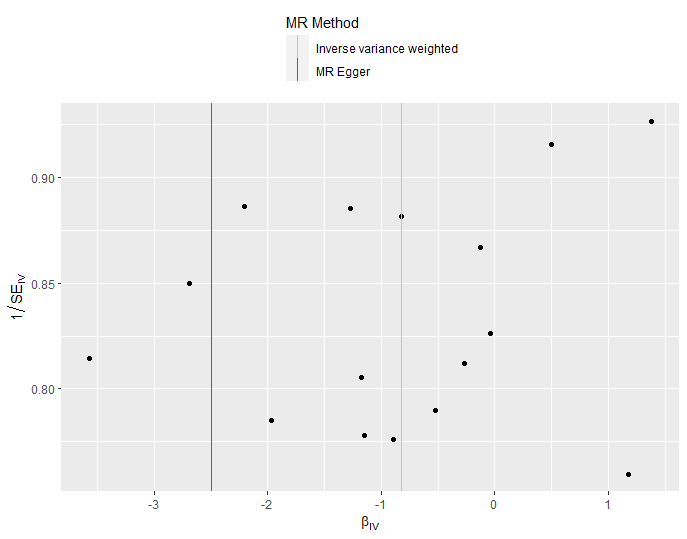 |

Figure note: A: Funnel plot of the causal effects of SNP associated with Appendicular lean mass on Hip osteoarthritis. B: Funnel plot of the causal effects of SNP associated with Low hand grip on Hip osteoarthritis. C: Funnel plot of the causal effects of SNP associated with Usual walking pace on Hip osteoarthritis.

**Supplementary Figure 5** Leave-one-out sensitivity analysis in the univariate Mendelian Randomization study of Sarcopenia phenotypes and Knee osteoarthritis

| A | B | C |
| --- | --- | --- |
| 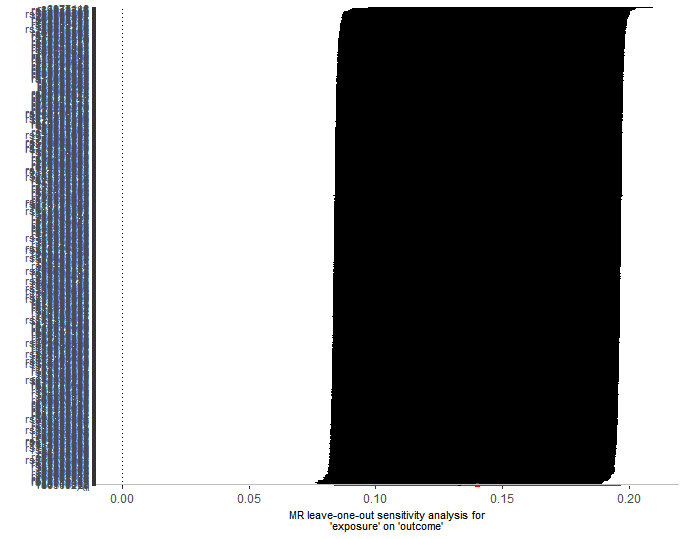 | 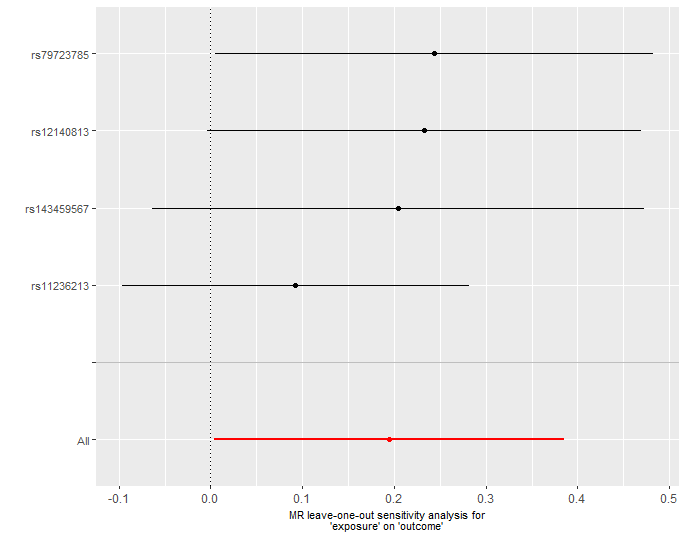 | 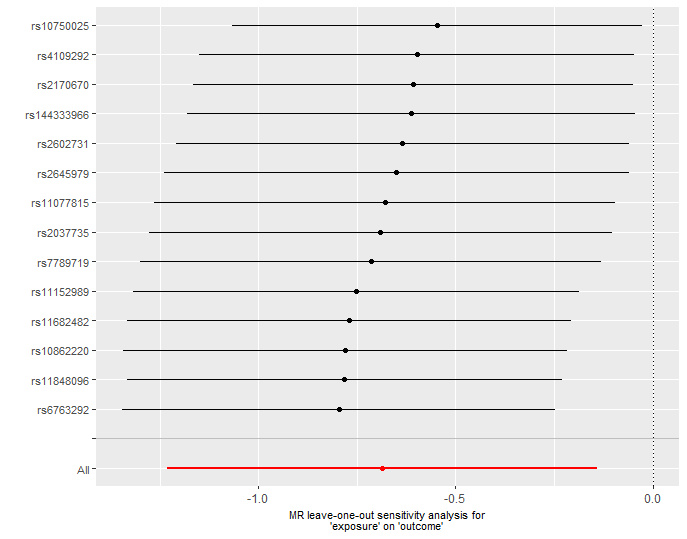 |

Figure note: A: Leave-one-out sensitivity analysis of the causal effects of Appendicular lean mass associated SNPs on Knee osteoarthritis. B: Leave-one-out sensitivity analysis of the causal effects of Low hand grip associated SNPs on Knee osteoarthritis. C: Leave-one-out sensitivity analysis of the causal effects of Usual walking pace associated SNPs on Knee osteoarthritis.

**Supplementary Figure 6** Leave-one-out sensitivity analysis in the univariate Mendelian Randomization study of Sarcopenia phenotypes and Hip osteoarthritis

| A | B | C |
| --- | --- | --- |
| 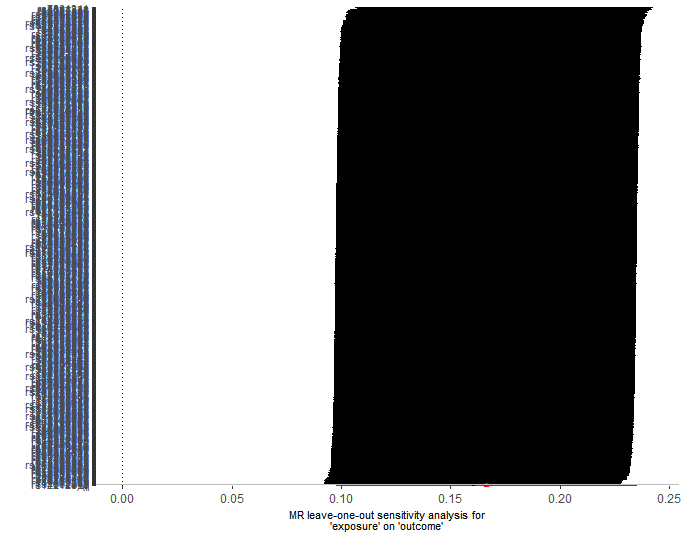 | 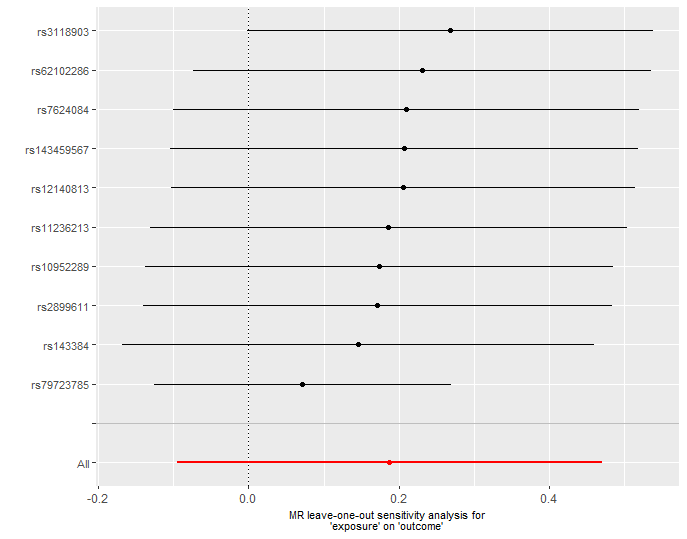 | 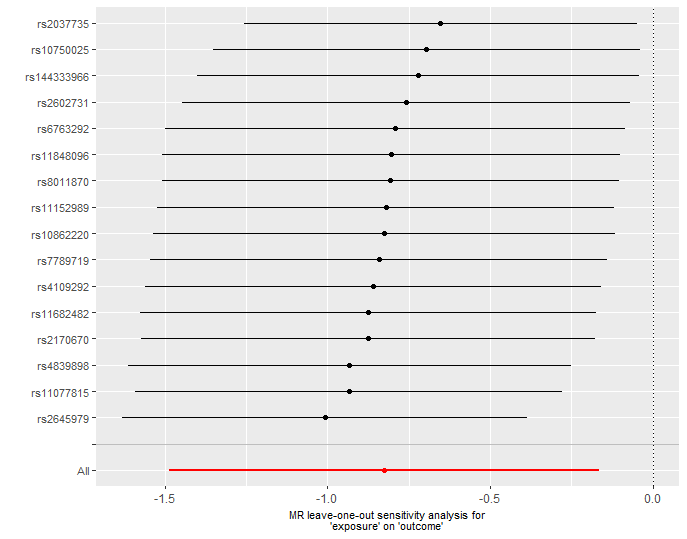 |

Figure note: A: Leave-one-out sensitivity analysis of the causal effects of Appendicular lean mass associated SNPs on Hip osteoarthritis. B: Leave-one-out sensitivity analysis of the causal effects of Low hand grip associated SNPs on Hip osteoarthritis. C: Leave-one-out sensitivity analysis of the causal effects of Usual walking pace associated SNPs on Hip osteoarthritis.
