# Supplementary material for: Causal relationship between sarcopenia with osteoarthritis and the mediating role of obesity: a univariate, multivariate, two-step Mendelian randomization study
Source: BMC Geriatr. 2024 May 29;24:469. doi: 10.1186/s12877-024-05098-8 (PMC11138082; doi:10.1186/s12877-024-05098-8)
Supplement: Supplementary file 3 — Supplementary Material 3. [file 12877_2024_5098_MOESM3_ESM.docx]

**Supplementary Material 3**

1. **Table 1** Results of a Univariate Mendelian Randomization Analysis of 91 Circulating Inflammatory Proteins and Knee Osteoarthritis 2
2. **Table 2** MR-Egger pleiotropy test results of univariate Mendelian randomization analysis of 91 Circulating Inflammatory Proteins and Knee osteoarthritis 9
3. **Table 3** Cochran Q heterogeneity test results of univariate Mendelian randomization analysis of 91 Circulating Inflammatory Proteins and Knee osteoarthritis 11

Table 1 Results of a Univariate Mendelian Randomization Analysis of 91 Circulating Inflammatory Proteins and Knee Osteoarthritis

| Exposure | Outcome | Method | nsnp | β | SE | 1. value | OR | OR_lci95 | OR_uci95 |
| --- | --- | --- | --- | --- | --- | --- | --- | --- | --- |
| Adenosine Deaminase levels | Knee osteoarthritis | Inverse variance weighted | 2 | -0.003997206 | 0.03536703 | 0.91001416 | 0.996010772 | 0.929306579 | 1.06750289 |
| beta-nerve growth factor levels | Knee osteoarthritis | Inverse variance weighted | 2 | -0.020094267 | 0.065107007 | 0.757599597 | 0.980106278 | 0.862686432 | 1.113508084 |
| Eotaxin levels | Knee osteoarthritis | Inverse variance weighted | 6 | -0.007888115 | 0.028055505 | 0.778586886 | 0.992142915 | 0.939059061 | 1.048227534 |
| Eotaxin levels | Knee osteoarthritis | MR Egger | 6 | 0.047182501 | 0.053861811 | 0.430488821 | 1.04831331 | 0.943285157 | 1.165035607 |
| Eotaxin levels | Knee osteoarthritis | Weighted median | 6 | -0.013777726 | 0.035022032 | 0.69402284 | 0.986316753 | 0.92088427 | 1.056398474 |
| Eotaxin levels | Knee osteoarthritis | Weighted mode | 6 | 0.035619843 | 0.040925192 | 0.423909091 | 1.036261829 | 0.956386142 | 1.122808592 |
| C-C motif chemokine 19 levels | Knee osteoarthritis | Inverse variance weighted | 2 | -0.059932191 | 0.076453591 | 0.433097096 | 0.941828396 | 0.810761598 | 1.094083304 |
| C-C motif chemokine 20 levels | Knee osteoarthritis | Inverse variance weighted | 2 | -0.038645239 | 0.054549956 | 0.478673555 | 0.962091961 | 0.864535291 | 1.070657209 |
| C-C motif chemokine 23 levels | Knee osteoarthritis | Inverse variance weighted | 4 | -0.042148125 | 0.019121118 | 0.027505293 | 0.958727759 | 0.923462104 | 0.995340157 |
| C-C motif chemokine 23 levels | Knee osteoarthritis | MR Egger | 4 | -0.061868495 | 0.074097365 | 0.491590935 | 0.940006495 | 0.812938896 | 1.086935579 |
| C-C motif chemokine 23 levels | Knee osteoarthritis | Weighted median | 4 | -0.0434855 | 0.019193454 | 0.023473301 | 0.957446437 | 0.92209717 | 0.994150844 |
| C-C motif chemokine 23 levels | Knee osteoarthritis | Weighted mode | 4 | -0.044530251 | 0.01953057 | 0.106932494 | 0.956446666 | 0.920525875 | 0.99376916 |
| C-C motif chemokine 25 levels | Knee osteoarthritis | Inverse variance weighted | 5 | 0.00797329 | 0.018335514 | 0.663667677 | 1.008005161 | 0.972423065 | 1.044889248 |
| C-C motif chemokine 25 levels | Knee osteoarthritis | MR Egger | 5 | 0.028968695 | 0.035689527 | 0.476393653 | 1.029392369 | 0.95984566 | 1.103978163 |
| C-C motif chemokine 25 levels | Knee osteoarthritis | Weighted median | 5 | 0.010298412 | 0.014541968 | 0.478830027 | 1.010351623 | 0.981960843 | 1.039563247 |
| C-C motif chemokine 25 levels | Knee osteoarthritis | Weighted mode | 5 | 0.017769639 | 0.017432285 | 0.365672734 | 1.017928458 | 0.98373607 | 1.053309295 |
| C-C motif chemokine 28 levels | Knee osteoarthritis | Inverse variance weighted | 4 | 0.001090583 | 0.085863878 | 0.989866097 | 1.001091178 | 0.846028174 | 1.184574672 |
| C-C motif chemokine 28 levels | Knee osteoarthritis | MR Egger | 4 | 0.076240466 | 0.260178844 | 0.797105518 | 1.079222059 | 0.648100133 | 1.797130094 |
| C-C motif chemokine 28 levels | Knee osteoarthritis | Weighted median | 4 | -0.000235876 | 0.071738348 | 0.997376559 | 0.999764152 | 0.868625642 | 1.150700959 |
| C-C motif chemokine 28 levels | Knee osteoarthritis | Weighted mode | 4 | -0.002588664 | 0.125345461 | 0.984819872 | 0.997414683 | 0.780152576 | 1.275181396 |
| C-C motif chemokine 4 levels | Knee osteoarthritis | Inverse variance weighted | 4 | 0.005449786 | 0.022042343 | 0.804721187 | 1.005464663 | 0.962950562 | 1.049855755 |
| C-C motif chemokine 4 levels | Knee osteoarthritis | MR Egger | 4 | 0.071601067 | 0.076142747 | 0.44630136 | 1.074226715 | 0.925298661 | 1.247124936 |
| C-C motif chemokine 4 levels | Knee osteoarthritis | Weighted median | 4 | -0.003819039 | 0.019849081 | 0.847425891 | 0.996188244 | 0.95817654 | 1.035707906 |
| C-C motif chemokine 4 levels | Knee osteoarthritis | Weighted mode | 4 | 0.032423982 | 0.020254521 | 0.207727888 | 1.032955367 | 0.992751518 | 1.074787367 |
| Natural killer cell receptor 2B4 levels | Knee osteoarthritis | Inverse variance weighted | 6 | -0.034957326 | 0.034599571 | 0.312332476 | 0.965646623 | 0.902332244 | 1.033403612 |
| Natural killer cell receptor 2B4 levels | Knee osteoarthritis | MR Egger | 6 | -0.06289881 | 0.08503152 | 0.500536152 | 0.93903849 | 0.794882806 | 1.109337476 |
| Natural killer cell receptor 2B4 levels | Knee osteoarthritis | Weighted median | 6 | -0.023878658 | 0.032706264 | 0.465332538 | 0.976404182 | 0.915776503 | 1.041045629 |
| Natural killer cell receptor 2B4 levels | Knee osteoarthritis | Weighted mode | 6 | -0.016193804 | 0.035148187 | 0.664338117 | 0.98393661 | 0.918434902 | 1.054109825 |
| CD40L receptor levels | Knee osteoarthritis | Inverse variance weighted | 3 | 0.023930756 | 0.017432179 | 0.169817009 | 1.024219394 | 0.989815899 | 1.059818668 |
| CD40L receptor levels | Knee osteoarthritis | MR Egger | 3 | 0.051881199 | 0.028390759 | 0.318762672 | 1.053250608 | 0.996242389 | 1.113521022 |
| CD40L receptor levels | Knee osteoarthritis | Weighted median | 3 | 0.026204382 | 0.016597937 | 0.114388027 | 1.026550735 | 0.993692403 | 1.060495591 |
| CD40L receptor levels | Knee osteoarthritis | Weighted mode | 3 | 0.0271577 | 0.018548255 | 0.280730442 | 1.027529831 | 0.990845283 | 1.06557257 |
| T-cell surface glycoprotein CD5 levels | Knee osteoarthritis | Inverse variance weighted | 3 | -0.071011931 | 0.070565455 | 0.314258242 | 0.931450779 | 0.81113545 | 1.069612423 |
| T-cell surface glycoprotein CD5 levels | Knee osteoarthritis | MR Egger | 3 | -0.180145925 | 0.820121582 | 0.862347735 | 0.835148334 | 0.167363995 | 4.167400147 |
| T-cell surface glycoprotein CD5 levels | Knee osteoarthritis | Weighted median | 3 | -0.061356748 | 0.045495283 | 0.177452693 | 0.940487662 | 0.860254071 | 1.028204426 |
| T-cell surface glycoprotein CD5 levels | Knee osteoarthritis | Weighted mode | 3 | -0.143095946 | 0.050314491 | 0.104592463 | 0.866670911 | 0.785282033 | 0.956495166 |
| T-cell surface glycoprotein CD6 isoform levels | Knee osteoarthritis | Inverse variance weighted | 2 | -0.099723594 | 0.079879767 | 0.211876852 | 0.905087555 | 0.773919097 | 1.058487232 |
| CUB domain-containing protein 1 levels | Knee osteoarthritis | Inverse variance weighted | 2 | -0.051054732 | 0.040574007 | 0.208279192 | 0.950226661 | 0.877586472 | 1.028879475 |
| Cystatin D levels | Knee osteoarthritis | Inverse variance weighted | 5 | 0.027329008 | 0.038474665 | 0.477510825 | 1.02770587 | 0.953056265 | 1.108202521 |
| Cystatin D levels | Knee osteoarthritis | MR Egger | 5 | 0.060931988 | 0.064901387 | 0.417072735 | 1.062826627 | 0.935873716 | 1.207000922 |
| Cystatin D levels | Knee osteoarthritis | Weighted median | 5 | 0.022847875 | 0.047274795 | 0.628883239 | 1.023110887 | 0.932570317 | 1.12244178 |
| Cystatin D levels | Knee osteoarthritis | Weighted mode | 5 | 0.022948496 | 0.05333226 | 0.689152231 | 1.023213838 | 0.921656454 | 1.135961837 |
| Fractalkine levels | Knee osteoarthritis | Inverse variance weighted | 3 | -0.026320341 | 0.109444294 | 0.809949829 | 0.97402302 | 0.785974321 | 1.207063409 |
| Fractalkine levels | Knee osteoarthritis | MR Egger | 3 | 0.427603286 | 0.843592725 | 0.701336657 | 1.533577568 | 0.293511501 | 8.012838178 |
| Fractalkine levels | Knee osteoarthritis | Weighted median | 3 | -0.048364809 | 0.07102362 | 0.49589177 | 0.952786139 | 0.828970174 | 1.095095403 |
| Fractalkine levels | Knee osteoarthritis | Weighted mode | 3 | -0.097532885 | 0.069231795 | 0.294253654 | 0.907072512 | 0.791973613 | 1.038898932 |
| C-X-C motif chemokine 1 levels | Knee osteoarthritis | Inverse variance weighted | 2 | 0.043829081 | 0.026263721 | 0.095155639 | 1.044803763 | 0.992381358 | 1.099995375 |
| C-X-C motif chemokine 10 levels | Knee osteoarthritis | Inverse variance weighted | 4 | -0.035835364 | 0.047765736 | 0.453115235 | 0.964799121 | 0.878573062 | 1.059487689 |
| C-X-C motif chemokine 10 levels | Knee osteoarthritis | MR Egger | 4 | 0.05422022 | 0.044188684 | 0.344653477 | 1.055717067 | 0.968129324 | 1.15122897 |
| C-X-C motif chemokine 10 levels | Knee osteoarthritis | Weighted median | 4 | 0.004060118 | 0.038371476 | 0.915732441 | 1.004068371 | 0.931324069 | 1.08249462 |
| C-X-C motif chemokine 10 levels | Knee osteoarthritis | Weighted mode | 4 | 0.013063178 | 0.036583667 | 0.744674986 | 1.013148874 | 0.943045441 | 1.088463605 |
| C-X-C motif chemokine 11 levels | Knee osteoarthritis | Inverse variance weighted | 4 | -0.000447057 | 0.081739838 | 0.995636178 | 0.999553043 | 0.851583995 | 1.173232812 |
| C-X-C motif chemokine 11 levels | Knee osteoarthritis | MR Egger | 4 | 0.19629774 | 0.199628364 | 0.42912522 | 1.216889168 | 0.822856172 | 1.799608848 |
| C-X-C motif chemokine 11 levels | Knee osteoarthritis | Weighted median | 4 | -0.007936305 | 0.044472463 | 0.858366158 | 0.992095104 | 0.90927988 | 1.082452958 |
| C-X-C motif chemokine 11 levels | Knee osteoarthritis | Weighted mode | 4 | -0.024040918 | 0.053877179 | 0.685681649 | 0.976245763 | 0.878411436 | 1.084976527 |
| C-X-C motif chemokine 5 levels | Knee osteoarthritis | Inverse variance weighted | 4 | 0.001652004 | 0.019974096 | 0.934084266 | 1.001653369 | 0.963197091 | 1.04164504 |
| C-X-C motif chemokine 5 levels | Knee osteoarthritis | MR Egger | 4 | -0.022133874 | 0.030012502 | 0.537612447 | 0.978109283 | 0.922232076 | 1.037372039 |
| C-X-C motif chemokine 5 levels | Knee osteoarthritis | Weighted median | 4 | -0.007061287 | 0.02065368 | 0.732433658 | 0.992963585 | 0.953569945 | 1.033984645 |
| C-X-C motif chemokine 5 levels | Knee osteoarthritis | Weighted mode | 4 | -0.006582025 | 0.02137044 | 0.778230979 | 0.993439589 | 0.952687741 | 1.035934624 |
| C-X-C motif chemokine 6 levels | Knee osteoarthritis | Inverse variance weighted | 3 | -0.006408008 | 0.016893253 | 0.704447633 | 0.993612479 | 0.961251898 | 1.027062481 |
| C-X-C motif chemokine 6 levels | Knee osteoarthritis | MR Egger | 3 | 0.004675065 | 0.03071813 | 0.903849163 | 1.00468601 | 0.945981307 | 1.067033748 |
| C-X-C motif chemokine 6 levels | Knee osteoarthritis | Weighted median | 3 | -0.005873052 | 0.017232556 | 0.733245553 | 0.994144161 | 0.961126868 | 1.028295687 |
| C-X-C motif chemokine 6 levels | Knee osteoarthritis | Weighted mode | 3 | -0.004043603 | 0.016905268 | 0.833234242 | 0.995964561 | 0.963504685 | 1.02951799 |
| C-X-C motif chemokine 9 levels | Knee osteoarthritis | Inverse variance weighted | 4 | -0.019888454 | 0.076379765 | 0.794563691 | 0.980308016 | 0.844008432 | 1.138618728 |
| C-X-C motif chemokine 9 levels | Knee osteoarthritis | MR Egger | 4 | 0.441942197 | 0.262321031 | 0.23407882 | 1.555725812 | 0.930338273 | 2.601508367 |
| C-X-C motif chemokine 9 levels | Knee osteoarthritis | Weighted median | 4 | -0.000802566 | 0.0512105 | 0.987496139 | 0.999197756 | 0.903774727 | 1.104695811 |
| C-X-C motif chemokine 9 levels | Knee osteoarthritis | Weighted mode | 4 | 0.04845001 | 0.066863185 | 0.521089398 | 1.049642899 | 0.920717674 | 1.196621121 |
| Delta and Notch-like epidermal growth factor-related receptor levels | Knee osteoarthritis | Inverse variance weighted | 2 | -0.008374372 | 0.050780482 | 0.869012295 | 0.991660595 | 0.897713668 | 1.095439193 |
| Protein S100-A12 levels | Knee osteoarthritis | Inverse variance weighted | 2 | -0.001558986 | 0.040098604 | 0.968987003 | 0.998442228 | 0.922975813 | 1.080079097 |
| Fibroblast growth factor 19 levels | Knee osteoarthritis | Inverse variance weighted | 3 | 0.076020715 | 0.033925448 | 0.025037943 | 1.078984925 | 1.009572361 | 1.15316991 |
| Fibroblast growth factor 19 levels | Knee osteoarthritis | MR Egger | 3 | 0.131934734 | 0.120426418 | 0.470988604 | 1.141033846 | 0.901134238 | 1.444799435 |
| Fibroblast growth factor 19 levels | Knee osteoarthritis | Weighted median | 3 | 0.074456753 | 0.03705613 | 0.044505699 | 1.077298752 | 1.001828407 | 1.158454475 |
| Fibroblast growth factor 19 levels | Knee osteoarthritis | Weighted mode | 3 | 0.089045724 | 0.042952547 | 0.173906884 | 1.093130638 | 1.004870556 | 1.189142805 |
| Fibroblast growth factor 21 levels | Knee osteoarthritis | Inverse variance weighted | 4 | -0.035732265 | 0.033841674 | 0.29102955 | 0.964898596 | 0.902973614 | 1.031070328 |
| Fibroblast growth factor 21 levels | Knee osteoarthritis | MR Egger | 4 | -0.037399132 | 0.14453638 | 0.820022145 | 0.963291578 | 0.725647851 | 1.27876168 |
| Fibroblast growth factor 21 levels | Knee osteoarthritis | Weighted median | 4 | -0.037841465 | 0.037752369 | 0.316169742 | 0.962865577 | 0.894190796 | 1.036814651 |
| Fibroblast growth factor 21 levels | Knee osteoarthritis | Weighted mode | 4 | -0.039914838 | 0.043566299 | 0.42713027 | 0.960871266 | 0.882227973 | 1.046524954 |
| Fibroblast growth factor 5 levels | Knee osteoarthritis | Inverse variance weighted | 2 | -0.024778269 | 0.015750166 | 0.115670926 | 0.975526192 | 0.945871462 | 1.00611065 |
| Fms-related tyrosine kinase 3 ligand levels | Knee osteoarthritis | Inverse variance weighted | 6 | -0.006179432 | 0.03952591 | 0.875765998 | 0.993839621 | 0.919752915 | 1.073894061 |
| Fms-related tyrosine kinase 3 ligand levels | Knee osteoarthritis | MR Egger | 6 | -0.033059169 | 0.058751508 | 0.603684109 | 0.967481313 | 0.862248191 | 1.085557617 |
| Fms-related tyrosine kinase 3 ligand levels | Knee osteoarthritis | Weighted median | 6 | -0.013863342 | 0.028152029 | 0.622404388 | 0.986232311 | 0.933288117 | 1.04217996 |
| Fms-related tyrosine kinase 3 ligand levels | Knee osteoarthritis | Weighted mode | 6 | -0.014536797 | 0.030499274 | 0.653728691 | 0.985568352 | 0.928378862 | 1.046280799 |
| Glial cell line-derived neurotrophic factor levels | Knee osteoarthritis | Inverse variance weighted | 3 | -0.037378778 | 0.041673312 | 0.369746868 | 0.963311185 | 0.887755891 | 1.045296852 |
| Glial cell line-derived neurotrophic factor levels | Knee osteoarthritis | MR Egger | 3 | -0.11803534 | 0.049826284 | 0.254289752 | 0.888664647 | 0.805981205 | 0.979830361 |
| Glial cell line-derived neurotrophic factor levels | Knee osteoarthritis | Weighted median | 3 | -0.040617464 | 0.030063635 | 0.176679517 | 0.960196369 | 0.905251758 | 1.018475865 |
| Glial cell line-derived neurotrophic factor levels | Knee osteoarthritis | Weighted mode | 3 | -0.056203186 | 0.030126266 | 0.203090817 | 0.945347035 | 0.891142735 | 1.002848345 |
| Hepatocyte growth factor levels | Knee osteoarthritis | Inverse variance weighted | 2 | -0.031417522 | 0.06302444 | 0.618133475 | 0.969070881 | 0.856461919 | 1.096485846 |
| Interleukin-10 levels | Knee osteoarthritis | Inverse variance weighted | 3 | -0.072225659 | 0.061499657 | 0.24023184 | 0.930320937 | 0.82467576 | 1.0494998 |
| Interleukin-10 levels | Knee osteoarthritis | MR Egger | 3 | 0.223756626 | 0.296098998 | 0.5880258 | 1.250766579 | 0.700054276 | 2.234708205 |
| Interleukin-10 levels | Knee osteoarthritis | Weighted median | 3 | -0.056174115 | 0.054601063 | 0.303569009 | 0.945374518 | 0.849427913 | 1.052158711 |
| Interleukin-10 levels | Knee osteoarthritis | Weighted mode | 3 | -0.044110803 | 0.064036534 | 0.562101356 | 0.95684793 | 0.843983438 | 1.084805603 |
| Interleukin-10 receptor subunit beta levels | Knee osteoarthritis | Inverse variance weighted | 3 | 0.011772777 | 0.017071973 | 0.490447821 | 1.011842349 | 0.978545209 | 1.046272497 |
| Interleukin-10 receptor subunit beta levels | Knee osteoarthritis | MR Egger | 3 | -0.05467221 | 0.058789138 | 0.523089526 | 0.946795448 | 0.843750098 | 1.062425499 |
| Interleukin-10 receptor subunit beta levels | Knee osteoarthritis | Weighted median | 3 | 0.00867708 | 0.017813262 | 0.62617791 | 1.008714835 | 0.974104283 | 1.044555123 |
| Interleukin-10 receptor subunit beta levels | Knee osteoarthritis | Weighted mode | 3 | 0.008000636 | 0.015710191 | 0.66119382 | 1.008032727 | 0.977466423 | 1.039554868 |
| Interleukin-12 subunit beta levels | Knee osteoarthritis | Inverse variance weighted | 11 | -0.021624897 | 0.020311962 | 0.287039551 | 0.978607245 | 0.940412809 | 1.018352931 |
| Interleukin-12 subunit beta levels | Knee osteoarthritis | MR Egger | 11 | 0.003318869 | 0.031329263 | 0.917957427 | 1.003324383 | 0.943568338 | 1.066864769 |
| Interleukin-12 subunit beta levels | Knee osteoarthritis | Weighted median | 11 | -0.004525082 | 0.018009505 | 0.80161249 | 0.99548514 | 0.960958829 | 1.031251948 |
| Interleukin-12 subunit beta levels | Knee osteoarthritis | Weighted mode | 11 | -0.005503366 | 0.017083826 | 0.753980771 | 0.994511749 | 0.961762572 | 1.028376076 |
| Interleukin-15 receptor subunit alpha levels | Knee osteoarthritis | Inverse variance weighted | 3 | 0.014239552 | 0.038239623 | 0.709612262 | 1.014341417 | 0.941096015 | 1.093287501 |
| Interleukin-15 receptor subunit alpha levels | Knee osteoarthritis | MR Egger | 3 | 0.080450645 | 0.077579283 | 0.48843406 | 1.083775356 | 0.930898761 | 1.261758067 |
| Interleukin-15 receptor subunit alpha levels | Knee osteoarthritis | Weighted median | 3 | 0.022329395 | 0.016721631 | 0.1817586 | 1.022580562 | 0.989609357 | 1.056650281 |
| Interleukin-15 receptor subunit alpha levels | Knee osteoarthritis | Weighted mode | 3 | 0.02133871 | 0.017928402 | 0.356085377 | 1.021568008 | 0.986293839 | 1.058103735 |
| Interleukin-18 levels | Knee osteoarthritis | Inverse variance weighted | 3 | -0.038448836 | 0.028275203 | 0.173890814 | 0.962280938 | 0.910402715 | 1.01711538 |
| Interleukin-18 levels | Knee osteoarthritis | MR Egger | 3 | -0.010282116 | 0.418336458 | 0.984355939 | 0.989770564 | 0.43595268 | 2.247137855 |
| Interleukin-18 levels | Knee osteoarthritis | Weighted median | 3 | -0.038894073 | 0.030899143 | 0.208123247 | 0.961852589 | 0.905329427 | 1.021904708 |
| Interleukin-18 levels | Knee osteoarthritis | Weighted mode | 3 | -0.038967149 | 0.033858528 | 0.368802076 | 0.961782304 | 0.900027586 | 1.027774276 |
| interleukin-18 receptor 1 levels | Knee osteoarthritis | Inverse variance weighted | 3 | -0.001050448 | 0.037552058 | 0.977683602 | 0.998950104 | 0.928065951 | 1.075248272 |
| interleukin-18 receptor 1 levels | Knee osteoarthritis | MR Egger | 3 | -0.168982447 | 0.525127716 | 0.801802064 | 0.844523727 | 0.301726962 | 2.363793812 |
| interleukin-18 receptor 1 levels | Knee osteoarthritis | Weighted median | 3 | 0.012119399 | 0.022649752 | 0.592595471 | 1.012193136 | 0.968241136 | 1.058140278 |
| interleukin-18 receptor 1 levels | Knee osteoarthritis | Weighted mode | 3 | 0.012518724 | 0.024523373 | 0.660477263 | 1.012597412 | 0.965077291 | 1.062457409 |
| Interleukin-8 levels | Knee osteoarthritis | Inverse variance weighted | 2 | 0.042868503 | 0.068492727 | 0.531391019 | 1.043800629 | 0.912673347 | 1.193767472 |
| Latency-associated peptide transforming growth factor beta 1 levels | Knee osteoarthritis | Inverse variance weighted | 2 | -0.175807169 | 0.049562771 | 0.000389405 | 0.838779711 | 0.761130691 | 0.924350326 |
| Leukemia inhibitory factor receptor levels | Knee osteoarthritis | Inverse variance weighted | 3 | -0.069462539 | 0.027881031 | 0.012724416 | 0.93289508 | 0.883283239 | 0.985293495 |
| Leukemia inhibitory factor receptor levels | Knee osteoarthritis | MR Egger | 3 | -0.023345859 | 0.054212458 | 0.741128095 | 0.976924548 | 0.878444739 | 1.086444632 |
| Leukemia inhibitory factor receptor levels | Knee osteoarthritis | Weighted median | 3 | -0.065320602 | 0.029120521 | 0.024889569 | 0.936767086 | 0.884797191 | 0.991789512 |
| Leukemia inhibitory factor receptor levels | Knee osteoarthritis | Weighted mode | 3 | -0.064094318 | 0.031703508 | 0.180584981 | 0.937916533 | 0.881409285 | 0.998046468 |
| Monocyte chemoattractant protein-1 levels | Knee osteoarthritis | Inverse variance weighted | 3 | -0.015013143 | 0.028174471 | 0.594128161 | 0.985098992 | 0.932174635 | 1.041028138 |
| Monocyte chemoattractant protein-1 levels | Knee osteoarthritis | MR Egger | 3 | -0.013734055 | 0.064268212 | 0.865970942 | 0.986359826 | 0.869619299 | 1.118771982 |
| Monocyte chemoattractant protein-1 levels | Knee osteoarthritis | Weighted median | 3 | -0.014828065 | 0.029840273 | 0.619249337 | 0.98528133 | 0.929308053 | 1.044625941 |
| Monocyte chemoattractant protein-1 levels | Knee osteoarthritis | Weighted mode | 3 | -0.011862545 | 0.034447854 | 0.763412003 | 0.988207537 | 0.923688542 | 1.057233139 |
| Monocyte chemoattractant protein 2 levels | Knee osteoarthritis | Inverse variance weighted | 3 | 0.00201641 | 0.01938693 | 0.917162438 | 1.002018444 | 0.964657683 | 1.04082617 |
| Monocyte chemoattractant protein 2 levels | Knee osteoarthritis | MR Egger | 3 | 0.015076944 | 0.029000636 | 0.694787053 | 1.015191175 | 0.959095811 | 1.074567431 |
| Monocyte chemoattractant protein 2 levels | Knee osteoarthritis | Weighted median | 3 | 0.004224867 | 0.019491881 | 0.828403118 | 1.004233804 | 0.96659159 | 1.043341928 |
| Monocyte chemoattractant protein 2 levels | Knee osteoarthritis | Weighted mode | 3 | 0.005993111 | 0.021157835 | 0.803607476 | 1.006011106 | 0.96514566 | 1.048606845 |
| Monocyte chemoattractant protein-3 levels | Knee osteoarthritis | Inverse variance weighted | 5 | 0.032714234 | 0.030864524 | 0.289176451 | 1.033255228 | 0.97260209 | 1.097690801 |
| Monocyte chemoattractant protein-3 levels | Knee osteoarthritis | MR Egger | 5 | 0.047027292 | 0.066808783 | 0.532207141 | 1.048150615 | 0.919506724 | 1.194792472 |
| Monocyte chemoattractant protein-3 levels | Knee osteoarthritis | Weighted median | 5 | 0.03853445 | 0.038232934 | 0.313509052 | 1.039286532 | 0.964252489 | 1.120159406 |
| Monocyte chemoattractant protein-3 levels | Knee osteoarthritis | Weighted mode | 5 | 0.039842362 | 0.042104126 | 0.397587219 | 1.040646716 | 0.958216317 | 1.130168176 |
| Monocyte chemoattractant protein-4 levels | Knee osteoarthritis | Inverse variance weighted | 6 | -0.013604641 | 0.019911769 | 0.494451252 | 0.986487483 | 0.948729357 | 1.025748331 |
| Monocyte chemoattractant protein-4 levels | Knee osteoarthritis | MR Egger | 6 | 0.053299284 | 0.058079852 | 0.410707669 | 1.054745266 | 0.941258729 | 1.181914752 |
| Monocyte chemoattractant protein-4 levels | Knee osteoarthritis | Weighted median | 6 | -0.017013309 | 0.02396363 | 0.477726621 | 0.9831306 | 0.938021861 | 1.030408584 |
| Monocyte chemoattractant protein-4 levels | Knee osteoarthritis | Weighted mode | 6 | -0.02103842 | 0.034829693 | 0.572175788 | 0.979181343 | 0.914566937 | 1.048360776 |
| Macrophage inflammatory protein 1a levels | Knee osteoarthritis | Inverse variance weighted | 6 | -0.020135893 | 0.036059317 | 0.576564268 | 0.980065481 | 0.913189234 | 1.051839324 |
| Macrophage inflammatory protein 1a levels | Knee osteoarthritis | MR Egger | 6 | 0.011261843 | 0.062523208 | 0.865813449 | 1.011325496 | 0.894684941 | 1.143172544 |
| Macrophage inflammatory protein 1a levels | Knee osteoarthritis | Weighted median | 6 | -0.015562399 | 0.019157634 | 0.416599954 | 0.984558069 | 0.948274405 | 1.022230049 |
| Macrophage inflammatory protein 1a levels | Knee osteoarthritis | Weighted mode | 6 | -0.012399021 | 0.019476923 | 0.55238375 | 0.987677531 | 0.950683776 | 1.026110816 |
| Matrix metalloproteinase-1 levels | Knee osteoarthritis | Inverse variance weighted | 7 | -0.004979275 | 0.028928339 | 0.863339688 | 0.995033102 | 0.940184808 | 1.053081123 |
| Matrix metalloproteinase-1 levels | Knee osteoarthritis | MR Egger | 7 | -0.064691006 | 0.055936781 | 0.299725937 | 0.937357057 | 0.840022085 | 1.045970419 |
| Matrix metalloproteinase-1 levels | Knee osteoarthritis | Weighted median | 7 | -0.024314337 | 0.036571133 | 0.50614611 | 0.975978875 | 0.908469686 | 1.048504732 |
| Matrix metalloproteinase-1 levels | Knee osteoarthritis | Weighted mode | 7 | -0.028207079 | 0.040435563 | 0.511551492 | 0.972187026 | 0.898111744 | 1.052371958 |
| Matrix metalloproteinase-10 levels | Knee osteoarthritis | Inverse variance weighted | 4 | -0.020110905 | 0.019978432 | 0.314112229 | 0.98008997 | 0.942453562 | 1.01922937 |
| Matrix metalloproteinase-10 levels | Knee osteoarthritis | MR Egger | 4 | -0.042699921 | 0.036977593 | 0.367527812 | 0.958198883 | 0.891209275 | 1.030223904 |
| Matrix metalloproteinase-10 levels | Knee osteoarthritis | Weighted median | 4 | -0.017657446 | 0.020090294 | 0.379453168 | 0.982497533 | 0.944561555 | 1.021957117 |
| Matrix metalloproteinase-10 levels | Knee osteoarthritis | Weighted mode | 4 | -0.01916417 | 0.022148537 | 0.450546629 | 0.981018295 | 0.939342324 | 1.02454331 |
| Osteoprotegerin levels | Knee osteoarthritis | Inverse variance weighted | 3 | -0.066392405 | 0.061389871 | 0.279480125 | 0.935763594 | 0.829678874 | 1.05541256 |
| Osteoprotegerin levels | Knee osteoarthritis | MR Egger | 3 | -0.423999613 | 0.435637748 | 0.508618302 | 0.654424135 | 0.278635835 | 1.537027528 |
| Osteoprotegerin levels | Knee osteoarthritis | Weighted median | 3 | -0.042166057 | 0.032648338 | 0.196522619 | 0.958710567 | 0.89928363 | 1.022064585 |
| Osteoprotegerin levels | Knee osteoarthritis | Weighted mode | 3 | -0.038554717 | 0.032493657 | 0.357255469 | 0.962179056 | 0.902810789 | 1.025451343 |
| Oncostatin-M levels | Knee osteoarthritis | Inverse variance weighted | 3 | -0.066533951 | 0.086321919 | 0.440845982 | 0.93563115 | 0.789997962 | 1.108111274 |
| Oncostatin-M levels | Knee osteoarthritis | MR Egger | 3 | 0.55556046 | 0.259110142 | 0.277822998 | 1.742917546 | 1.048860757 | 2.89624867 |
| Oncostatin-M levels | Knee osteoarthritis | Weighted median | 3 | -0.000180917 | 0.05945257 | 0.997572003 | 0.999819099 | 0.889845031 | 1.123384631 |
| Oncostatin-M levels | Knee osteoarthritis | Weighted mode | 3 | 0.00676953 | 0.06540297 | 0.927006195 | 1.006792495 | 0.885661647 | 1.144490261 |
| Stem cell factor levels | Knee osteoarthritis | Inverse variance weighted | 9 | -0.000945689 | 0.025305681 | 0.970189498 | 0.999054758 | 0.950711313 | 1.049856456 |
| Stem cell factor levels | Knee osteoarthritis | MR Egger | 9 | -0.005458328 | 0.061766018 | 0.932056844 | 0.994556541 | 0.881156769 | 1.122550207 |
| Stem cell factor levels | Knee osteoarthritis | Weighted median | 9 | 0.001825322 | 0.030926718 | 0.952935473 | 1.001826989 | 0.942903773 | 1.06443239 |
| Stem cell factor levels | Knee osteoarthritis | Weighted mode | 9 | 0.001962297 | 0.037613239 | 0.9596721 | 1.001964224 | 0.930754575 | 1.078621941 |
| Signaling lymphocytic activation molecule levels | Knee osteoarthritis | Inverse variance weighted | 6 | -0.017523631 | 0.046187699 | 0.704390553 | 0.982629014 | 0.897581351 | 1.075735118 |
| Signaling lymphocytic activation molecule levels | Knee osteoarthritis | MR Egger | 6 | 0.152609101 | 0.109444641 | 0.235656851 | 1.164869543 | 0.939974628 | 1.443572 |
| Signaling lymphocytic activation molecule levels | Knee osteoarthritis | Weighted median | 6 | 0.01793582 | 0.040764002 | 0.659943168 | 1.018097633 | 0.939918956 | 1.102778897 |
| Signaling lymphocytic activation molecule levels | Knee osteoarthritis | Weighted mode | 6 | 0.038282436 | 0.045638963 | 0.43982372 | 1.039024649 | 0.950117203 | 1.136251632 |
| Sulfotransferase 1A1 levels | Knee osteoarthritis | Inverse variance weighted | 3 | 0.018145477 | 0.027637298 | 0.511465435 | 1.018311107 | 0.964617503 | 1.074993463 |
| Sulfotransferase 1A1 levels | Knee osteoarthritis | MR Egger | 3 | 0.074723895 | 0.098552255 | 0.587000158 | 1.077586583 | 0.888306422 | 1.307198523 |
| Sulfotransferase 1A1 levels | Knee osteoarthritis | Weighted median | 3 | 0.018955516 | 0.031026292 | 0.541232619 | 1.019136312 | 0.959007852 | 1.083034743 |
| Sulfotransferase 1A1 levels | Knee osteoarthritis | Weighted mode | 3 | 0.021813669 | 0.033713616 | 0.583957934 | 1.022053327 | 0.95670038 | 1.091870583 |
| TNF-beta levels | Knee osteoarthritis | Inverse variance weighted | 2 | -0.039564104 | 0.138789164 | 0.775593398 | 0.961208334 | 0.732281068 | 1.261703329 |
| Tumor necrosis factor ligand superfamily member 14 levels | Knee osteoarthritis | Inverse variance weighted | 4 | -0.017295515 | 0.024913579 | 0.487544006 | 0.982853194 | 0.936012797 | 1.0320376 |
| Tumor necrosis factor ligand superfamily member 14 levels | Knee osteoarthritis | MR Egger | 4 | -0.043679329 | 0.036367448 | 0.352672637 | 0.957260874 | 0.89140222 | 1.027985303 |
| Tumor necrosis factor ligand superfamily member 14 levels | Knee osteoarthritis | Weighted median | 4 | -0.022247174 | 0.027618506 | 0.420521451 | 0.97799847 | 0.926464597 | 1.032398874 |
| Tumor necrosis factor ligand superfamily member 14 levels | Knee osteoarthritis | Weighted mode | 4 | -0.022748338 | 0.028234792 | 0.479367524 | 0.977508454 | 0.924882543 | 1.033128786 |
| TNF-related apoptosis-inducing ligand levels | Knee osteoarthritis | Inverse variance weighted | 9 | -0.037380555 | 0.022165004 | 0.091705826 | 0.963309473 | 0.922356045 | 1.00608127 |
| TNF-related apoptosis-inducing ligand levels | Knee osteoarthritis | MR Egger | 9 | -0.059010448 | 0.03648933 | 0.149868962 | 0.94269692 | 0.877630573 | 1.012587199 |
| TNF-related apoptosis-inducing ligand levels | Knee osteoarthritis | Weighted median | 9 | -0.013514551 | 0.025217718 | 0.592017358 | 0.986576361 | 0.938998613 | 1.036564807 |
| TNF-related apoptosis-inducing ligand levels | Knee osteoarthritis | Weighted mode | 9 | -0.007503882 | 0.026009803 | 0.780296411 | 0.992524202 | 0.943194183 | 1.04443423 |
| TNF-related activation-induced cytokine levels | Knee osteoarthritis | Inverse variance weighted | 6 | 0.00200593 | 0.030658117 | 0.947832432 | 1.002007943 | 0.943570704 | 1.064064319 |
| TNF-related activation-induced cytokine levels | Knee osteoarthritis | MR Egger | 6 | -0.035359867 | 0.078207575 | 0.674610266 | 0.965257989 | 0.828079004 | 1.125161948 |
| TNF-related activation-induced cytokine levels | Knee osteoarthritis | Weighted median | 6 | -0.003124684 | 0.029148335 | 0.914630791 | 0.996880193 | 0.941524019 | 1.05549099 |
| TNF-related activation-induced cytokine levels | Knee osteoarthritis | Weighted mode | 6 | -0.014789322 | 0.030627809 | 0.649586402 | 0.985319503 | 0.927910656 | 1.046280175 |
| Tumor necrosis factor ligand superfamily member 12 levels | Knee osteoarthritis | Inverse variance weighted | 5 | 0.00092024 | 0.038033315 | 0.980696557 | 1.000920664 | 0.929019958 | 1.078386064 |
| Tumor necrosis factor ligand superfamily member 12 levels | Knee osteoarthritis | MR Egger | 5 | 0.109360786 | 0.085499043 | 0.290830802 | 1.115564758 | 0.943444879 | 1.319085786 |
| Tumor necrosis factor ligand superfamily member 12 levels | Knee osteoarthritis | Weighted median | 5 | 0.036229989 | 0.034626245 | 0.295415234 | 1.036894293 | 0.968857776 | 1.109708567 |
| Tumor necrosis factor ligand superfamily member 12 levels | Knee osteoarthritis | Weighted mode | 5 | 0.039130113 | 0.039417787 | 0.377045653 | 1.039905781 | 0.962589 | 1.123432776 |
| Urokinase-type plasminogen activator levels | Knee osteoarthritis | Inverse variance weighted | 8 | -0.045536623 | 0.030393736 | 0.134075077 | 0.955484609 | 0.900226981 | 1.014134054 |
| Urokinase-type plasminogen activator levels | Knee osteoarthritis | MR Egger | 8 | -0.045732591 | 0.072490632 | 0.551380255 | 0.955297383 | 0.828768654 | 1.101143348 |
| Urokinase-type plasminogen activator levels | Knee osteoarthritis | Weighted median | 8 | -0.051916819 | 0.034657867 | 0.134138243 | 0.949407836 | 0.88705682 | 1.016141489 |
| Urokinase-type plasminogen activator levels | Knee osteoarthritis | Weighted mode | 8 | -0.065960169 | 0.04505282 | 0.186587469 | 0.936168152 | 0.85704599 | 1.022594841 |
| Vascular endothelial growth factor A levels | Knee osteoarthritis | Inverse variance weighted | 4 | 0.011416324 | 0.015498401 | 0.46135771 | 1.011481739 | 0.981218078 | 1.042678821 |
| Vascular endothelial growth factor A levels | Knee osteoarthritis | MR Egger | 4 | 0.021973907 | 0.028677248 | 0.523612807 | 1.022217111 | 0.966345837 | 1.081318698 |
| Vascular endothelial growth factor A levels | Knee osteoarthritis | Weighted median | 4 | 0.014231775 | 0.01573354 | 0.365703803 | 1.014333529 | 0.983531155 | 1.046100576 |
| Vascular endothelial growth factor A levels | Knee osteoarthritis | Weighted mode | 4 | 0.014298279 | 0.016299433 | 0.444942808 | 1.014400988 | 0.982506214 | 1.047331152 |

**Table 2** MR-Egger pleiotropy test results of univariate Mendelian randomization analysis of 91 Circulating Inflammatory Proteins and Knee osteoarthritis

| Exposure | Outcome | SE | egger_ intercept | P-value |
| --- | --- | --- | --- | --- |
| Eotaxin levels | Knee osteoarthritis | 0.007012532 | -0.008399317 | 0.297131847 |
| C-C motif chemokine 23 levels | Knee osteoarthritis | 0.033720451 | 0.009289019 | 0.808805717 |
| C-C motif chemokine 25 levels | Knee osteoarthritis | 0.012411836 | -0.008739647 | 0.532083001 |
| C-C motif chemokine 28 levels | Knee osteoarthritis | 0.02087526 | -0.006561966 | 0.783022137 |
| C-C motif chemokine 4 levels | Knee osteoarthritis | 0.033778629 | -0.030744367 | 0.458807094 |
| Natural killer cell receptor 2B4 levels | Knee osteoarthritis | 0.009832612 | 0.003612843 | 0.731909892 |
| CD40L receptor levels | Knee osteoarthritis | 0.008808405 | -0.01091594 | 0.432234642 |
| T-cell surface glycoprotein CD5 levels | Knee osteoarthritis | 0.116131902 | 0.015567362 | 0.915167485 |
| Cystatin D levels | Knee osteoarthritis | 0.007587239 | -0.005102142 | 0.549449015 |
| Fractalkine levels | Knee osteoarthritis | 0.095259743 | -0.051936014 | 0.682228837 |
| C-X-C motif chemokine 10 levels | Knee osteoarthritis | 0.008134467 | -0.021662843 | 0.116808077 |
| C-X-C motif chemokine 11 levels | Knee osteoarthritis | 0.027864855 | -0.029953111 | 0.394866785 |
| C-X-C motif chemokine 5 levels | Knee osteoarthritis | 0.007242474 | 0.007690366 | 0.399572281 |
| C-X-C motif chemokine 6 levels | Knee osteoarthritis | 0.00896671 | -0.003873533 | 0.74040129 |
| C-X-C motif chemokine 9 levels | Knee osteoarthritis | 0.030462605 | -0.054977478 | 0.212875778 |
| Fibroblast growth factor 19 levels | Knee osteoarthritis | 0.015068345 | -0.007291549 | 0.713085886 |
| Fibroblast growth factor 21 levels | Knee osteoarthritis | 0.019288953 | 0.00022881 | 0.991612423 |
| Fms-related tyrosine kinase 3 ligand levels | Knee osteoarthritis | 0.008608168 | 0.005632374 | 0.548626605 |
| Glial cell line-derived neurotrophic factor levels | Knee osteoarthritis | 0.011428385 | 0.022903153 | 0.294651328 |
| Interleukin-10 levels | Knee osteoarthritis | 0.038797044 | -0.039627423 | 0.493259553 |
| Interleukin-10 receptor subunit beta levels | Knee osteoarthritis | 0.02147985 | 0.025370351 | 0.447255297 |
| Interleukin-12 subunit beta levels | Knee osteoarthritis | 0.005628611 | -0.005867816 | 0.324373691 |
| Interleukin-15 receptor subunit alpha levels | Knee osteoarthritis | 0.019271003 | -0.018953552 | 0.505286946 |
| Interleukin-18 levels | Knee osteoarthritis | 0.08016696 | -0.005410037 | 0.957102995 |
| interleukin-18 receptor 1 levels | Knee osteoarthritis | 0.136916396 | 0.043989237 | 0.802094967 |
| Leukemia inhibitory factor receptor levels | Knee osteoarthritis | 0.010471172 | -0.010386322 | 0.502589815 |
| Monocyte chemoattractant protein-1 levels | Knee osteoarthritis | 0.011184276 | -0.00024766 | 0.98590525 |
| Monocyte chemoattractant protein 2 levels | Knee osteoarthritis | 0.010431889 | -0.006317011 | 0.653367529 |
| Monocyte chemoattractant protein-3 levels | Knee osteoarthritis | 0.00822877 | -0.001987763 | 0.824689132 |
| Monocyte chemoattractant protein-4 levels | Knee osteoarthritis | 0.010222884 | -0.012535767 | 0.287356871 |
| Macrophage inflammatory protein 1a levels | Knee osteoarthritis | 0.012898808 | -0.008210147 | 0.559048732 |
| Matrix metalloproteinase-1 levels | Knee osteoarthritis | 0.007669459 | 0.009565551 | 0.267549056 |
| Matrix metalloproteinase-10 levels | Knee osteoarthritis | 0.015467769 | 0.011641669 | 0.530192805 |
| Osteoprotegerin levels | Knee osteoarthritis | 0.069906488 | 0.058071379 | 0.55870621 |
| Oncostatin-M levels | Knee osteoarthritis | 0.024628705 | -0.06025782 | 0.247010236 |
| Stem cell factor levels | Knee osteoarthritis | 0.008466288 | 0.00068798 | 0.937508952 |
| Signaling lymphocytic activation molecule levels | Knee osteoarthritis | 0.013399535 | -0.022348592 | 0.170668788 |
| Sulfotransferase 1A1 levels | Knee osteoarthritis | 0.015389237 | -0.009204226 | 0.656850745 |
| Tumor necrosis factor ligand superfamily member 14 levels | Knee osteoarthritis | 0.008707646 | 0.0086716 | 0.424246377 |
| TNF-related apoptosis-inducing ligand levels | Knee osteoarthritis | 0.007171206 | 0.005441142 | 0.47277231 |
| TNF-related activation-induced cytokine levels | Knee osteoarthritis | 0.012190186 | 0.006430163 | 0.625758725 |
| Tumor necrosis factor ligand superfamily member 12 levels | Knee osteoarthritis | 0.011780723 | -0.016312231 | 0.260170029 |
| Urokinase-type plasminogen activator levels | Knee osteoarthritis | 0.008869523 | 2.69E-05 | 0.997679035 |
| Vascular endothelial growth factor A levels | Knee osteoarthritis | 0.008513103 | -0.003768793 | 0.70125584 |
| Body mass index | Knee osteoarthritis | 0.001714087 | -0.002318357 | 0.177142861 |

**Table 3** Cochran Q heterogeneity test results of univariate Mendelian randomization analysis of 91 Circulating Inflammatory Proteins and Knee osteoarthritis

| Exposure | Outcome | Method | Q_pval |
| --- | --- | --- | --- |
| Adenosine Deaminase levels | Knee osteoarthritis | Inverse variance weighted | 0.190967499 |
| beta-nerve growth factor levels | Knee osteoarthritis | Inverse variance weighted | 0.941215091 |
| Eotaxin levels | Knee osteoarthritis | MR Egger | 0.932878726 |
| Eotaxin levels | Knee osteoarthritis | Inverse variance weighted | 0.809851091 |
| C-C motif chemokine 19 levels | Knee osteoarthritis | Inverse variance weighted | 0.053490952 |
| C-C motif chemokine 20 levels | Knee osteoarthritis | Inverse variance weighted | 0.441335892 |
| C-C motif chemokine 23 levels | Knee osteoarthritis | MR Egger | 0.920620791 |
| C-C motif chemokine 23 levels | Knee osteoarthritis | Inverse variance weighted | 0.970662024 |
| C-C motif chemokine 25 levels | Knee osteoarthritis | MR Egger | 0.036540258 |
| C-C motif chemokine 25 levels | Knee osteoarthritis | Inverse variance weighted | 0.041825292 |
| C-C motif chemokine 28 levels | Knee osteoarthritis | MR Egger | 0.031514679 |
| C-C motif chemokine 28 levels | Knee osteoarthritis | Inverse variance weighted | 0.064164341 |
| C-C motif chemokine 4 levels | Knee osteoarthritis | MR Egger | 0.111667829 |
| C-C motif chemokine 4 levels | Knee osteoarthritis | Inverse variance weighted | 0.102251627 |
| Natural killer cell receptor 2B4 levels | Knee osteoarthritis | MR Egger | 0.067888729 |
| Natural killer cell receptor 2B4 levels | Knee osteoarthritis | Inverse variance weighted | 0.107605107 |
| CD40L receptor levels | Knee osteoarthritis | MR Egger | 0.475938247 |
| CD40L receptor levels | Knee osteoarthritis | Inverse variance weighted | 0.359886808 |
| T-cell surface glycoprotein CD5 levels | Knee osteoarthritis | MR Egger | 0.014317505 |
| T-cell surface glycoprotein CD5 levels | Knee osteoarthritis | Inverse variance weighted | 0.047208661 |
| T-cell surface glycoprotein CD6 isoform levels | Knee osteoarthritis | Inverse variance weighted | 0.079539167 |
| CUB domain-containing protein 1 levels | Knee osteoarthritis | Inverse variance weighted | 0.977091186 |
| Cystatin D levels | Knee osteoarthritis | MR Egger | 0.313247961 |
| Cystatin D levels | Knee osteoarthritis | Inverse variance weighted | 0.393308839 |
| Fractalkine levels | Knee osteoarthritis | MR Egger | 0.012232674 |
| Fractalkine levels | Knee osteoarthritis | Inverse variance weighted | 0.01705518 |
| C-X-C motif chemokine 1 levels | Knee osteoarthritis | Inverse variance weighted | 0.360984505 |
| C-X-C motif chemokine 10 levels | Knee osteoarthritis | MR Egger | 0.504720048 |
| C-X-C motif chemokine 10 levels | Knee osteoarthritis | Inverse variance weighted | 0.037409935 |
| C-X-C motif chemokine 11 levels | Knee osteoarthritis | MR Egger | 0.00155434 |
| C-X-C motif chemokine 11 levels | Knee osteoarthritis | Inverse variance weighted | 0.000139855 |
| C-X-C motif chemokine 5 levels | Knee osteoarthritis | MR Egger | 0.413259603 |
| C-X-C motif chemokine 5 levels | Knee osteoarthritis | Inverse variance weighted | 0.408120146 |
| C-X-C motif chemokine 6 levels | Knee osteoarthritis | MR Egger | 0.797776304 |
| C-X-C motif chemokine 6 levels | Knee osteoarthritis | Inverse variance weighted | 0.88149692 |
| C-X-C motif chemokine 9 levels | Knee osteoarthritis | MR Egger | 0.095850765 |
| C-X-C motif chemokine 9 levels | Knee osteoarthritis | Inverse variance weighted | 0.006340652 |
| Delta and Notch-like epidermal growth factor-related receptor levels | Knee osteoarthritis | Inverse variance weighted | 0.567134119 |
| Protein S100-A12 levels | Knee osteoarthritis | Inverse variance weighted | 0.670368996 |
| Fibroblast growth factor 19 levels | Knee osteoarthritis | MR Egger | 0.900811487 |
| Fibroblast growth factor 19 levels | Knee osteoarthritis | Inverse variance weighted | 0.882632832 |
| Fibroblast growth factor 21 levels | Knee osteoarthritis | MR Egger | 0.901048253 |
| Fibroblast growth factor 21 levels | Knee osteoarthritis | Inverse variance weighted | 0.976200059 |
| Fibroblast growth factor 5 levels | Knee osteoarthritis | Inverse variance weighted | 0.611496124 |
| Fms-related tyrosine kinase 3 ligand levels | Knee osteoarthritis | MR Egger | 0.013219191 |
| Fms-related tyrosine kinase 3 ligand levels | Knee osteoarthritis | Inverse variance weighted | 0.015708983 |
| Glial cell line-derived neurotrophic factor levels | Knee osteoarthritis | MR Egger | 0.924098228 |
| Glial cell line-derived neurotrophic factor levels | Knee osteoarthritis | Inverse variance weighted | 0.133632245 |
| Hepatocyte growth factor levels | Knee osteoarthritis | Inverse variance weighted | 0.195629375 |
| Interleukin-10 levels | Knee osteoarthritis | MR Egger | 0.176323228 |
| Interleukin-10 levels | Knee osteoarthritis | Inverse variance weighted | 0.154448362 |
| Interleukin-10 receptor subunit beta levels | Knee osteoarthritis | MR Egger | 0.6952967 |
| Interleukin-10 receptor subunit beta levels | Knee osteoarthritis | Inverse variance weighted | 0.461057912 |
| Interleukin-12 subunit beta levels | Knee osteoarthritis | MR Egger | 0.028930949 |
| Interleukin-12 subunit beta levels | Knee osteoarthritis | Inverse variance weighted | 0.022289706 |
| Interleukin-15 receptor subunit alpha levels | Knee osteoarthritis | MR Egger | 0.022017176 |
| Interleukin-15 receptor subunit alpha levels | Knee osteoarthritis | Inverse variance weighted | 0.005748897 |
| Interleukin-18 levels | Knee osteoarthritis | MR Egger | 0.59507189 |
| Interleukin-18 levels | Knee osteoarthritis | Inverse variance weighted | 0.866300391 |
| interleukin-18 receptor 1 levels | Knee osteoarthritis | MR Egger | 0.023381161 |
| interleukin-18 receptor 1 levels | Knee osteoarthritis | Inverse variance weighted | 0.058702941 |
| Interleukin-8 levels | Knee osteoarthritis | Inverse variance weighted | 0.285298543 |
| Latency-associated peptide transforming growth factor beta 1 levels | Knee osteoarthritis | Inverse variance weighted | 0.257486185 |
| Leukemia inhibitory factor receptor levels | Knee osteoarthritis | MR Egger | 0.457809904 |
| Leukemia inhibitory factor receptor levels | Knee osteoarthritis | Inverse variance weighted | 0.46414813 |
| Monocyte chemoattractant protein-1 levels | Knee osteoarthritis | MR Egger | 0.901763498 |
| Monocyte chemoattractant protein-1 levels | Knee osteoarthritis | Inverse variance weighted | 0.992167698 |
| Monocyte chemoattractant protein 2 levels | Knee osteoarthritis | MR Egger | 0.618971826 |
| Monocyte chemoattractant protein 2 levels | Knee osteoarthritis | Inverse variance weighted | 0.735649192 |
| Monocyte chemoattractant protein-3 levels | Knee osteoarthritis | MR Egger | 0.416309499 |
| Monocyte chemoattractant protein-3 levels | Knee osteoarthritis | Inverse variance weighted | 0.574298923 |
| Monocyte chemoattractant protein-4 levels | Knee osteoarthritis | MR Egger | 0.877515135 |
| Monocyte chemoattractant protein-4 levels | Knee osteoarthritis | Inverse variance weighted | 0.745013948 |
| Macrophage inflammatory protein 1a levels | Knee osteoarthritis | MR Egger | 0.00115601 |
| Macrophage inflammatory protein 1a levels | Knee osteoarthritis | Inverse variance weighted | 0.001258929 |
| Matrix metalloproteinase-1 levels | Knee osteoarthritis | MR Egger | 0.53554852 |
| Matrix metalloproteinase-1 levels | Knee osteoarthritis | Inverse variance weighted | 0.463221107 |
| Matrix metalloproteinase-10 levels | Knee osteoarthritis | MR Egger | 0.261825879 |
| Matrix metalloproteinase-10 levels | Knee osteoarthritis | Inverse variance weighted | 0.328725674 |
| Osteoprotegerin levels | Knee osteoarthritis | MR Egger | 0.03264075 |
| Osteoprotegerin levels | Knee osteoarthritis | Inverse variance weighted | 0.021127742 |
| Oncostatin-M levels | Knee osteoarthritis | MR Egger | 0.950750835 |
| Oncostatin-M levels | Knee osteoarthritis | Inverse variance weighted | 0.050038857 |
| Stem cell factor levels | Knee osteoarthritis | MR Egger | 0.228046797 |
| Stem cell factor levels | Knee osteoarthritis | Inverse variance weighted | 0.312400231 |
| Signaling lymphocytic activation molecule levels | Knee osteoarthritis | MR Egger | 0.175311867 |
| Signaling lymphocytic activation molecule levels | Knee osteoarthritis | Inverse variance weighted | 0.056674609 |
| Sulfotransferase 1A1 levels | Knee osteoarthritis | MR Egger | 0.593568661 |
| Sulfotransferase 1A1 levels | Knee osteoarthritis | Inverse variance weighted | 0.725233518 |
| TNF-beta levels | Knee osteoarthritis | Inverse variance weighted | 0.000804643 |
| Tumor necrosis factor ligand superfamily member 14 levels | Knee osteoarthritis | MR Egger | 0.961763787 |
| Tumor necrosis factor ligand superfamily member 14 levels | Knee osteoarthritis | Inverse variance weighted | 0.784390614 |
| TNF-related apoptosis-inducing ligand levels | Knee osteoarthritis | MR Egger | 0.133573587 |
| TNF-related apoptosis-inducing ligand levels | Knee osteoarthritis | Inverse variance weighted | 0.149787328 |
| TNF-related activation-induced cytokine levels | Knee osteoarthritis | MR Egger | 0.096472525 |
| TNF-related activation-induced cytokine levels | Knee osteoarthritis | Inverse variance weighted | 0.134700288 |
| Tumor necrosis factor ligand superfamily member 12 levels | Knee osteoarthritis | MR Egger | 0.267897364 |
| Tumor necrosis factor ligand superfamily member 12 levels | Knee osteoarthritis | Inverse variance weighted | 0.16734237 |
| Urokinase-type plasminogen activator levels | Knee osteoarthritis | MR Egger | 0.158410996 |
| Urokinase-type plasminogen activator levels | Knee osteoarthritis | Inverse variance weighted | 0.233144488 |
| Vascular endothelial growth factor A levels | Knee osteoarthritis | MR Egger | 0.34782904 |
| Vascular endothelial growth factor A levels | Knee osteoarthritis | Inverse variance weighted | 0.508879353 |
| Body mass index | Knee osteoarthritis | MR Egger | 1.46×10^-26^ |
| Body mass index | Knee osteoarthritis | Inverse variance weighted | 7.81×10^-27^ |
